# ATR senses stiff extracellular matrix to promote epithelial-to-mesenchymal transition and immune suppression

**DOI:** 10.1172/JCI192285

**Published:** 2026-07-07

**Authors:** Xinyi Tu, Xiangyu Zeng, Yaoliang Sun, Yaobin Ouyang, Lingling Zhu, Ping Yin, Kevin Pavelko, Roberto Leon-Ferre, Yanxia Jiang, Haidong Dong, Jodi Carter, Shouhai Zhu, Jann N. Sarkaria, Liewei Wang, Jinzhou Huang, Kuntian Luo, Yiqun Han, Zheming Wu, Zhenkun Lou, Robert W. Mutter

**Affiliations:** 1 Department of Radiation Oncology,; 2 Molecular Pharmacology and Experimental Therapeutics,; 3 Department of Immunology,; 4 Department of Oncology,; 5 Department of Medical Oncology, and; 6 Department of Urology, Mayo Clinic, Rochester, Minnesota, USA.; 7 Department of Laboratory Medicine and Pathology, University of Alberta, Edmonton, Alberta, Canada.

**Keywords:** Cell biology, Immunology, Oncology, Breast cancer, Cellular immune response, Extracellular matrix

## Abstract

Our research uncovers a role for ATR in responding to ECM stiffness and promoting epithelial-to-mesenchymal transition (EMT) and metastasis. ATR, when deubiquitinated and upregulated by USP21 under enhanced ECM stiffness conditions, phosphorylates the nuclear protein SUN2, which promotes β-catenin nuclear translocation and EMT. ATM-mediated EMT promotes polymorphonuclear myeloid-derived suppressor cell recruitment and inhibits CD103^+^ dendritic cells, fostering an immunosuppressive tumor milieu. ATR inhibition disrupts this malignant cascade by promoting mesenchymal-to-epithelial transition to enhance antitumor immunity and mitigate metastases. Consistently, circulating HLA-DR^+^ dendritic cells were also enhanced following treatment with the ATR inhibitor berzosertib in patients with therapeutically resistant early-stage breast cancer. Our data suggest that ATR-targeted therapy may be optimized by considering both DNA damage–dependent and EMT-inducing effects of ATR

## Abstract 2

ATR promotes metastasis and immunosuppression through EMT process

## Graphical abstract

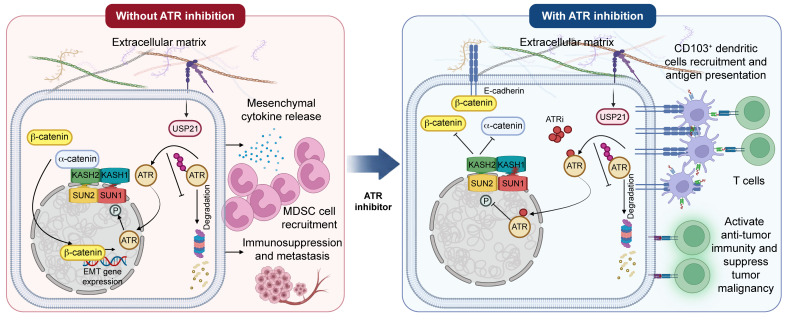

## Introduction

CD103^+^ dendritic cells and tissue-resident memory T cells contribute to antitumor immunity and immunotherapy responsiveness (1–5). Conversely, epithelial-to-mesenchymal transition (EMT) reduces E-cadherin expression and promotes immune evasion through impaired CD103-mediated interactions and recruitment of immunosuppressive cell populations (6–8).

In many solid tumors, increased ECM stiffness promotes tumor progression through activation of mechanotransduction pathways that drive migration, invasion, and metastasis (9, 10). The LINC complex, composed of SUN- and KASH-domain proteins, mechanically couples the cytoskeleton and nucleus and plays a critical role in sensing and responding to ECM stiffness (10–15).

Although ECM stiffness promotes migration, invasion, immunosuppression, and metastasis, the pathways by which cancer cells sense and respond to mechanical cues remain incompletely defined (16–18). ATR is best known for its role in DNA damage and replication stress responses (19), but recent studies have implicated ATR in cellular responses to mechanical stress (20, 21). Here, we show that ECM stiffness upregulates ATR through USP21, leading to SUN2 phosphorylation, β-catenin nuclear translocation, EMT, and immune evasion. ATR inhibition reverses these stiffness-induced effects and promotes E-cadherin expression and CD103^+^ dendritic cell infiltration, supporting a broader role for ATR inhibitors in cancer therapy.

## Results

### ECM stiffness induces ATR expression through USP21.

**Figure 1:**
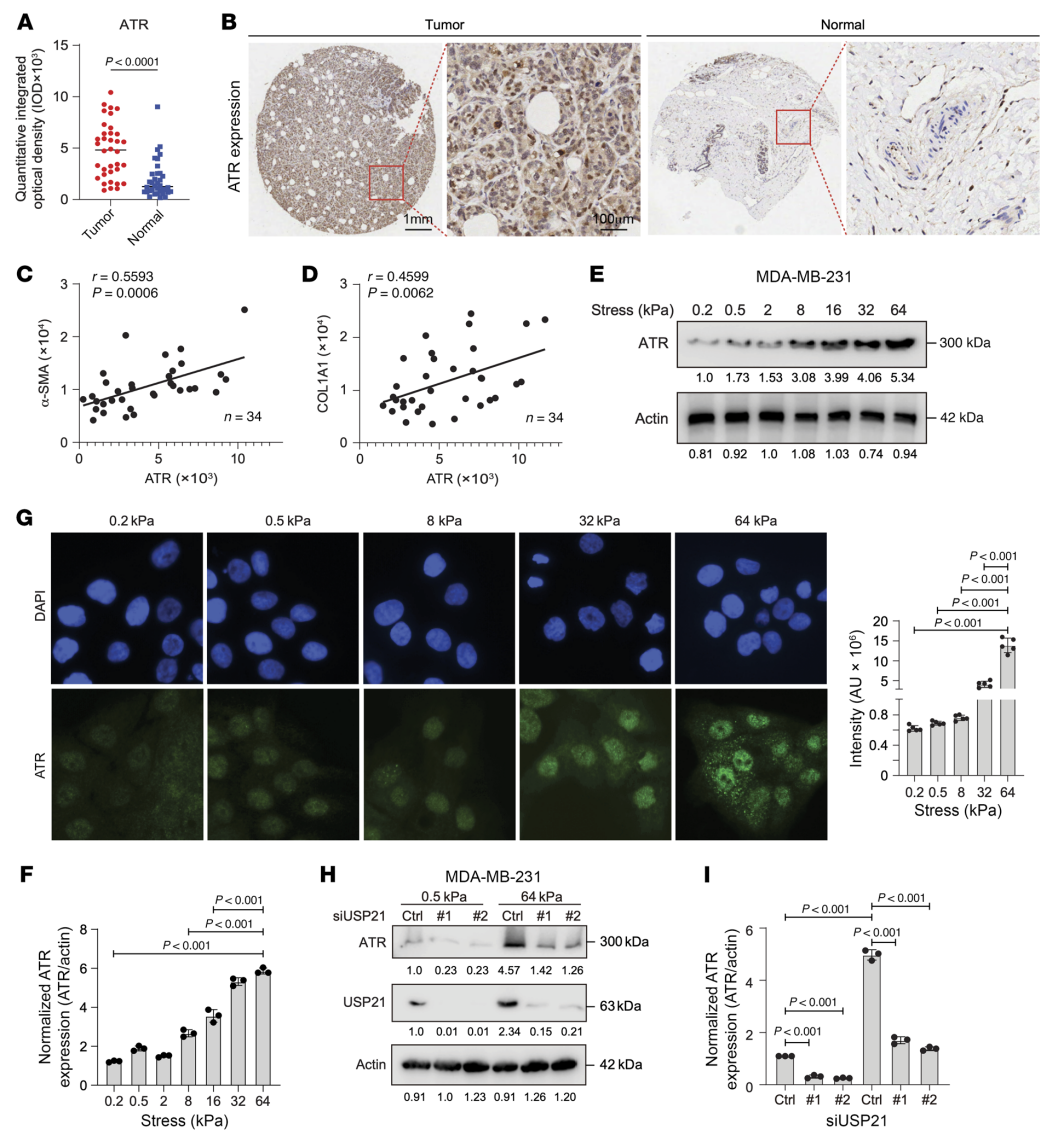
ECM stiffness induces ATR expression through USP21. (**A** and **B**) Tumors were harvested from 36 TNBC patients and processed to create FFPE blocks. These blocks were then sectioned and subjected to IHC analysis. ATR IHC analysis of 36 TNBC primary tumor samples and their adjacent normal tissues are shown. Statistical significance was determined using 2-tailed Student’s *t* test (*P* < 0.0001). Scale bars: 1 mm (left), 100 μm (right). (**C** and **D**) IHC analysis of 34 TNBC primary tumor samples staining with ATR and 2 fibrosis markers, α-SMA and COL1A1. The results, quantified as integrated optical density, revealed a positive correlation between the level of ATR and the 2 fibrosis markers. Pearson’s correlation analysis showed that ATR expression was positively correlated with α-SMA expression (*r* = 0.5593, *P* = 0.0006) and COL1A1 expression (*r* = 0.4599, *P* = 0.0062). (**E**) The expression of the indicated proteins in MDA-MB-231 cells cultured on surfaces with varying stiffness was analyzed by Western blotting. Actin served as a loading control. (**F**) Statistical analysis of the Western blot results in **E** by 1-way ANOVA (*n* = 3). (**G**) MCF-10A cells were cultured on substrates with different stiffnesses for 48 hours, after which ATR expression was assessed by immunofluorescence. Statistical significance was determined using 1-way ANOVA (*n* = 3). Original magnification, ×20. (**H**) MDA-MB-231 cells transfected with control or USP21-targeted siRNA were cultured on surfaces with indicated stiffness for 48 hours. USP21 and ATR expression levels were then determined. (**I**) Statistical analysis of the Western blot results in **H** by 2-way ANOVA (*n* = 3). Data are presented as mean ± SD.

**Figure 2:**
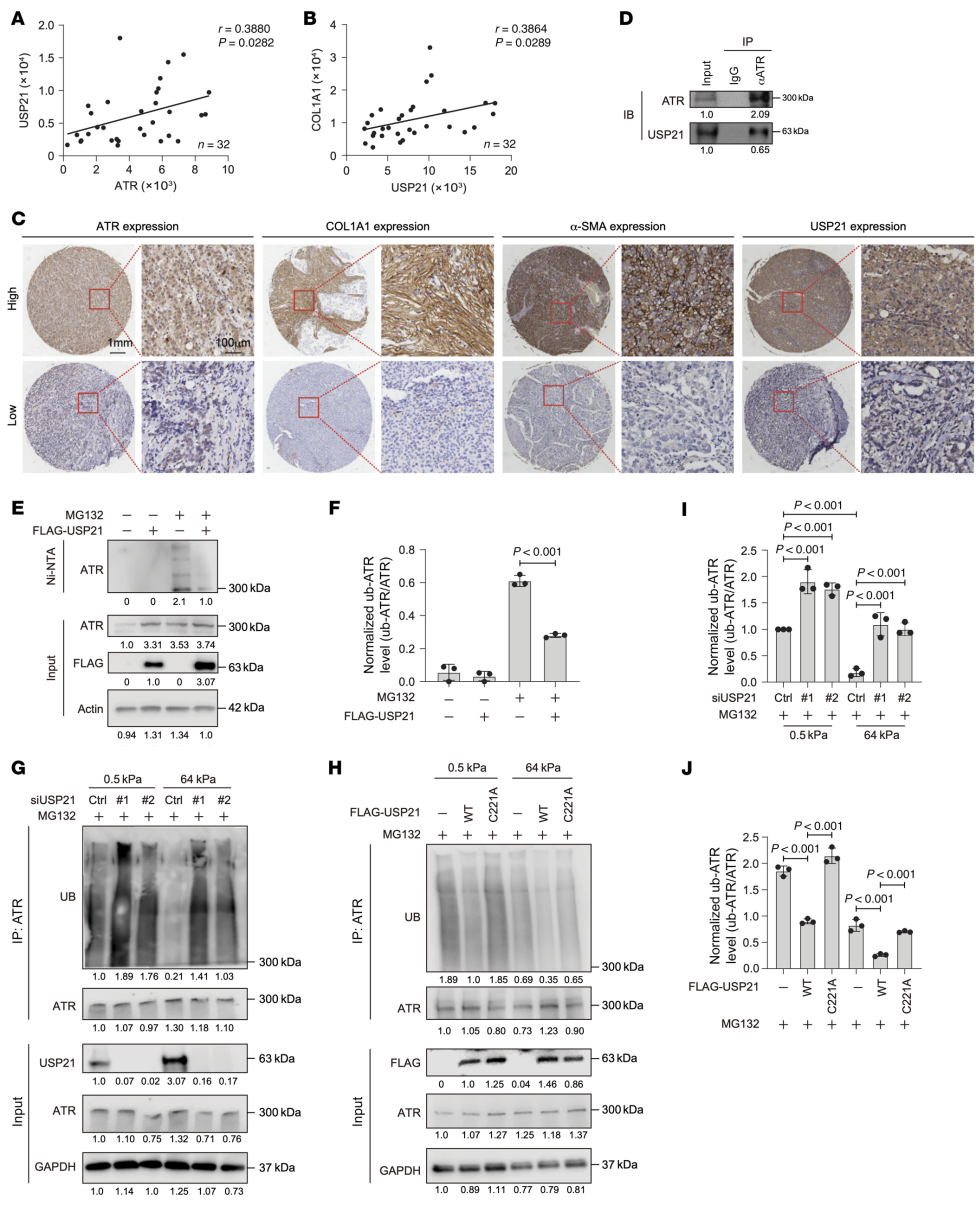
ECM stiffness regulates ATR levels through USP21. (**A** and **B**) USP21 expression was highly correlated with ATR and COL1A1 expression. The COL1A1, ATR, and USP21 levels were determined by IHC. (**C**) Representative IHC images for Figure 1, C and D, and for this figure, **A** and **B**. Scale bars: 1 mm (left), 100 μm (right). (**D**) The interaction between USP21 and ATR in MDA-MB-231 cells was assessed by IP using ATR antibody. (**E**) MDA-MB-231 cells were infected with lentivirus encoding either vector or USP21, treated with MG132 for 6 hours, and harvested for ubiquitination analysis. (**F**) Statistical analysis of the Western blot results in **E** by 1-way ANOVA (*n* = 3). (**G**) MDA-MB-231 cells were transfected with either control or USP21-specific siRNA, cultured on surfaces with different stiffnesses, and harvested for IP using ATR antibody to assess ubiquitination. (**H**) MDA-MB-231 cells were infected with lentivirus encoding either vector, WT USP21, or C221A USP21 mutant and seeded on surfaces with different stiffnesses. After 48 hours, the cells were harvested and IP was performed using ATR antibody to assess ubiquitination. (**I** and **J**) Statistical analyses of the Western blot results in **G** and **H** by 2-way ANOVA (*n* = 3). Data are presented as mean ± SD. Western blot and co-IP experiments were performed using at least 3 independent biological replicates, yielding similar results.

Surprisingly, despite ATR’s tumor-suppressive function in the cellular response to DNA damage and replication stress, we observed that ATR protein levels were higher in triple-negative breast cancer (TNBC) compared with adjacent normal breast tissue (Figure 1, A and B). Collagen type Iα1 (COL1A1), upregulated in breast cancer and other cancers (Supplemental Figure 1A; supplemental material available online with this article; https://doi.org/10.1172/JCI192285DS1), is the major component of collagen type I (22–24). ATR protein was positively correlated with COL1A1 expression as well as α-SMA, an actin isoform that generates mechanical tension and a marker of activated fibrogenic cells (*r* = 0.5593 and *P* = 0.0006; *r* = 0.4599 and *P* = 0.0062, respectively; Figure 1, C and D, and Figure 2C). We examined ATR expression in MDA-MB-231 TNBC cells grown on collagen-coated acrylamide hydrogels of varying stiffness (Figure 1, E and F) matching physiological elasticities of normal mammary tissue (~0.2–0.4 kPa) as well as primary and metastatic breast cancers (~1.0–64 kPa) (25–31). ATR was upregulated in MDA-MB-231 cells by increasing the stiffness of the surface (Figure 1, E and F). Similar findings were observed in the noncancerous breast cell line MCF-10A, and this effect was confirmed by both Western blot and immunofluorescence analyses (Figure 1G and Supplemental Figure 1, B and C). In contrast, ATR RNA expression was not impacted by the stiffness of the culture surface (Supplemental Figure 1, D and E). These results suggested the possibility that ECM stiffness may induce ATR protein expression at the posttranslational level. We also found that high stiffness does not affect cell apoptosis but increases the proportion of cells in the S phase, which is consistent with the enhanced cell growth observed under high-stiffness stimulation (Supplemental Figure 1, F–I) (32).

Protein ubiquitination and deubiquitylation can regulate protein expression, with ubiquitinated proteins being degraded by the proteasome or lysosome (33). In a screen of known deubiquitinating enzymes that remove ubiquitin from proteins, we identified USP21 as a potential regulator of ATR levels. We observed an increase in USP21 levels when MDA-MB-231 and MDA-MB-436 cells were cultured on high-stiffness ECM (Figure 1, H and I, and Supplemental Figure 1, J and K). Furthermore, the upregulation of ATR under high-stiffness conditions was found to be dependent on USP21 (Figure 1, H and I, and Supplemental Figure 1, J and K). Moreover, the USP21 level was positively correlated with both ATR and COL1A protein levels in TNBC specimens (Figure 2, A–C).

Based on these observations, we hypothesized that USP21 might enhance ATR expression in response to stiffness by deubiquitinating ATR. To test this hypothesis, we first examined whether USP21 could bind with ATR. Co-IP experiments confirmed an interaction between USP21 and ATR in MDA-MB-231 and MDA-MB-436 cells (Figure 2D and Supplemental Figure 1L). We next generated several USP21 truncations to identify the USP21 binding motif responsible for interacting with ATR. Only the C-terminus of USP21, which possesses deubiquitinating enzyme activity, was able to interact with ATR, while the USP21 N-terminus could not (Supplemental Figure 1, M and N).

We then overexpressed either WT USP21 or control vector in MDA-MB-231 cells. Overexpression of USP21 led to a notable decrease in ATR ubiquitination and an increase in ATR protein levels (Figure 2, E and F).

We further asked whether stiff ECM could impact USP21-mediated ATR deubiquitylation. Indeed, when MDA-MB-231 cells were cultured on surfaces with high stiffness, there was a reduction in ATR ubiquitination. However, this effect was negated by USP21 knockdown (Figure 2, G and I). Similarly, overexpression of WT USP21, but not the enzyme-inactive C221A mutant (34), reduced ATR ubiquitination under both low- and high-stiffness conditions (Figure 2, H and J, and Supplemental Figure 1, O and P). Collectively, these results indicated that USP21 modulates ATR levels in response to ECM stiffness through its deubiquitinating enzyme activity.

### ATR promotes ECM stiffness-induced invasion and migration.

**Figure 3:**
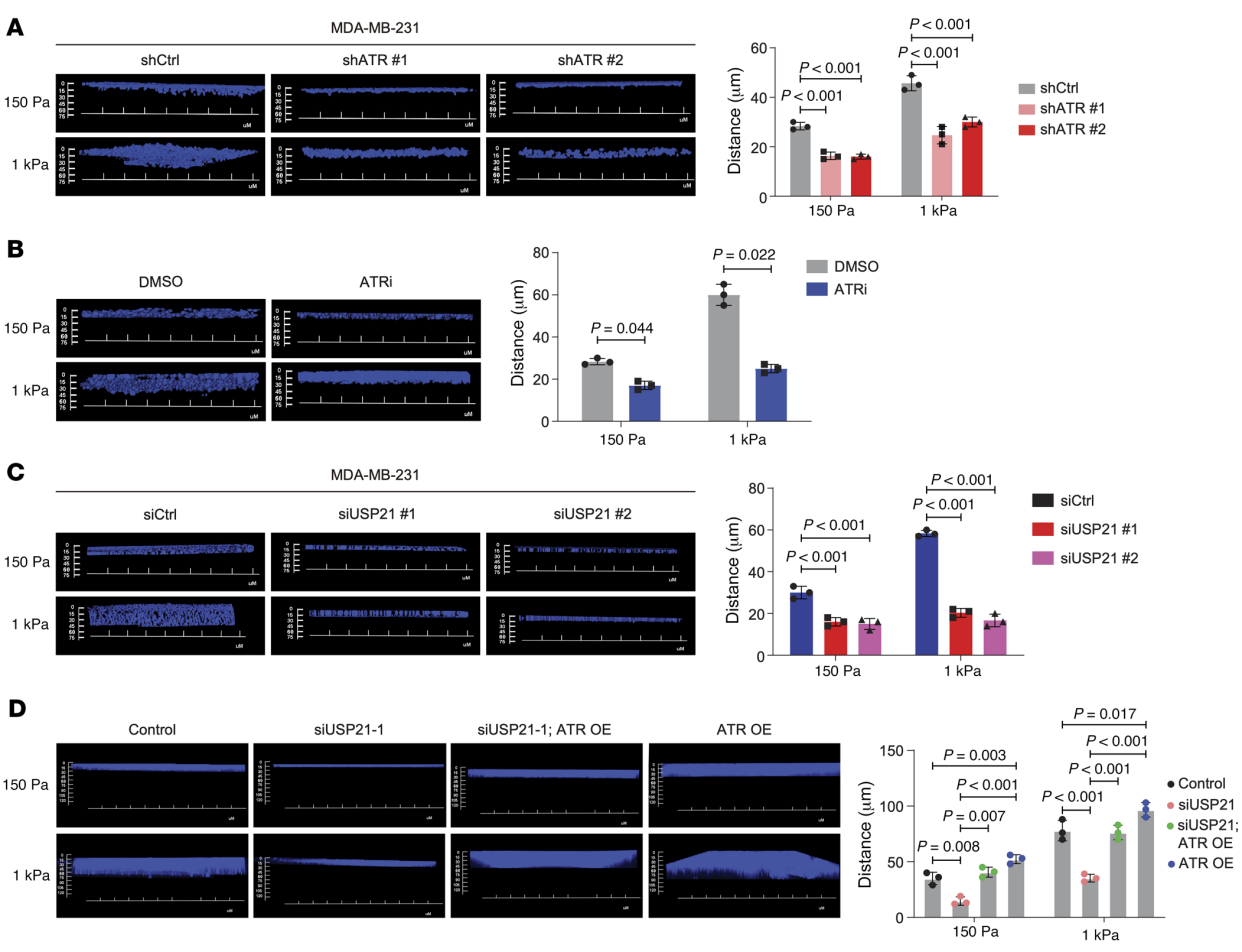
ATR promotes ECM stiffness–induced invasion. (**A**) Control or ATR shRNA-depleted MDA-MB-231 cells were seeded on collagen type I gels in serum-free condition. After 3 days, cells were stained with DAPI and the invasion depth was measured (*n* = 3). (**B**) ATR inhibition reduced the invasion of MDA-MB-231. MDA-MB-231 cells treated with either DMSO or VX970 (80 nM) were seeded on gel surfaces with different stiffnesses and incubated for 3 days in serum-free condition. The invasion depth was measured by DAPI staining (*n* = 3). Statistical analysis was performed using 2-way ANOVA. (**C**) Either control or USP21-depleted MDA-MB-231 cells were seeded on collagen gel surfaces with different stiffnesses, and the invasion depth was measured by DAPI staining after 3 days under serum-free conditions (*n* = 3). (**D**) Control, USP21-knockdown, USP21-knockdown plus ATR overexpression, or ATR-overexpressing MDA-MB-231 cells were seeded onto gel substrates of varying stiffness and cultured under serum-free conditions for 3 days. Invasion depth was quantified by DAPI staining (*n* = 3). Statistical analysis was performed using 2-way ANOVA. Data are presented as mean ± SD.

We next evaluated whether ATR might play a role in cancer cell migration and invasion, since ATR has been reported to respond to mechanical signals and mechanical stress induced by stiff tumor stroma is known to promote these malignant features (35–37). The stiffness of collagen gels that support cell invasion typically ranges from 0.1 to 10 kPa. This range provides an optimal environment for cells to migrate and invade, mimicking the mechanical properties of natural tissues (38). Therefore, we planted MDA-MB-231 cells on collagen type I cell surfaces with different stiffness levels (150 Pa or 1 kPa) in a serum-free medium to examine the effect of ATR on migration and invasion without significant replication stress and cell proliferation. Increased ECM stiffness enhanced TNBC cell invasion under these conditions (Figure 3A), aligning with previous studies (39). In contrast, ATR knockdown or pharmacological inhibition using the selective ATR inhibitor (ATRi) berzosertib (VX-970) reversed this stiffness-induced invasive phenotype in MDA-MB-231 cells cultured on collagen gels of varying stiffness (Figure 3, A and B).

Consistent with our findings that USP21 regulates ATR levels in an ECM stiffness-dependent manner (Figure 1H and Supplemental Figure 1J), USP21 knockdown markedly reduced the invasiveness of both MDA-MB-231 and MDA-MB-436 cells (Figure 3C and Supplemental Figure 2C).

For cells to invade, they must traverse several tissue boundaries and navigate through narrow confinements in the ECM before they can intravasate into circulatory or lymphatic vessels (40). To simulate this process, we measured the ability of cells to invade collagen gels through a microchannel in a serum-free medium and found that ATR knockdown hindered MDA-MB-231 cell invasion in this model (Supplemental Figure 2D). Furthermore, we found that ATR overexpression alone is sufficient to promote invasion in USP21-knockdown cells. Notably, ATR overexpression also drives invasion under conditions of low substrate stiffness (Figure 3D). Therefore, we concluded that ATR acts downstream of USP21 to promote the EMT process and enhance invasion. We further evaluated the role of ATR in migration. We cultured the highly malignant TNBC cell lines MDA-MB-231 and MDA-MB-436 in a serum-free medium and found that ATR inhibition and ATR knockdown both significantly reduced the migratory potential of MDA-MB-231 and MDA-MB-436 (Supplemental Figure 2, E–H). Furthermore, the effect of ATR on migration was noticeable even when proliferation was further inhibited by mitomycin C (Supplemental Figure 2, I and J). In our previously published study, ATRi monotherapy had minimal impact on TNBC cell clonogenic survival or patient-derived tumor xenograft growth (41), also suggesting that the reduction in migration and invasion following ATR knockdown or inhibition was not primarily due to suppressed proliferation, increased replication stress, or cell death. Taken together, these findings suggested that ATR promotes TNBC migration and invasion.

### ATR induces EMT through the LINC complex.

**Figure 4:**
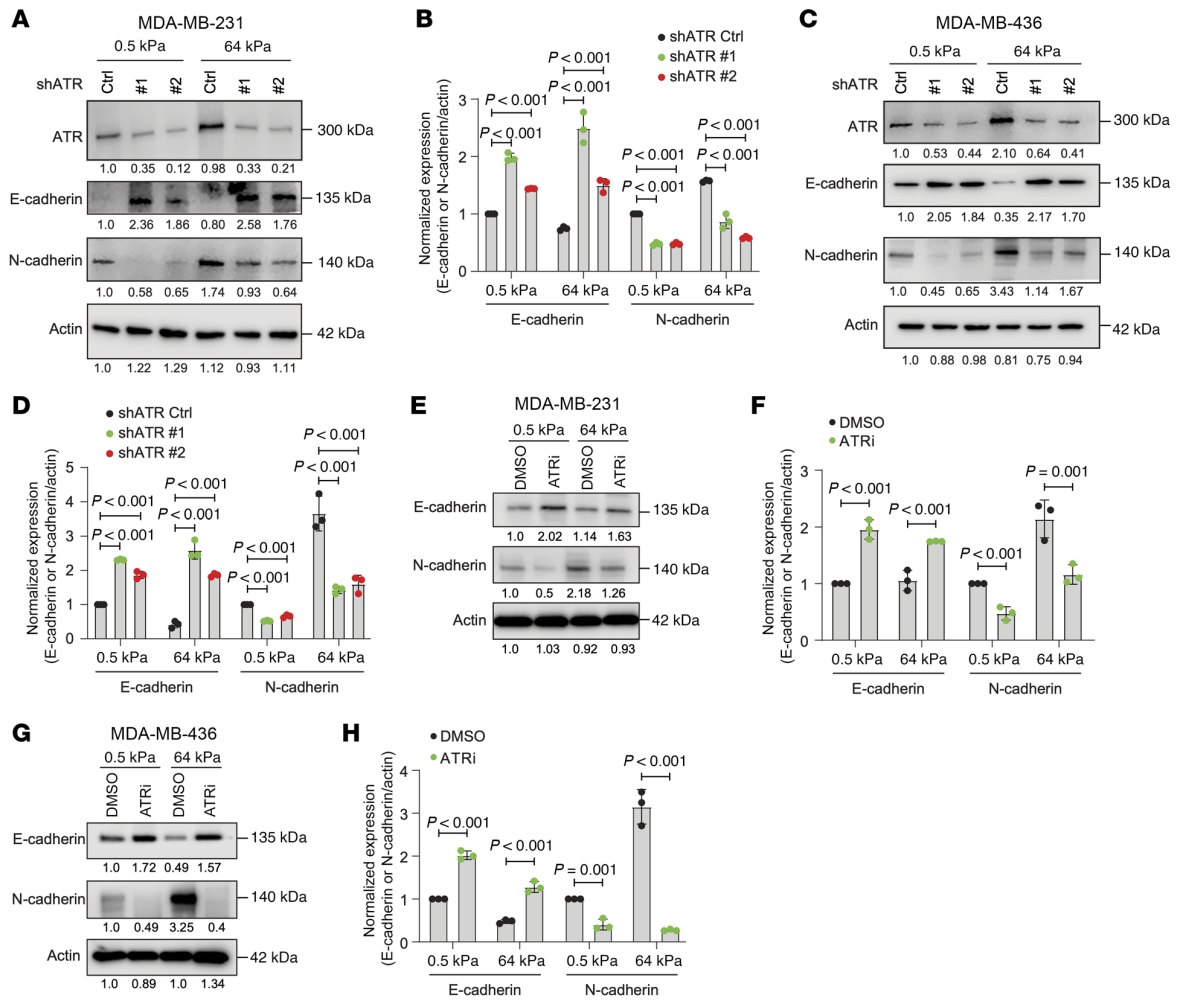
ATR promotes ECM stiffness–induced EMT. (**A**) ATR knockdown decreases N-cadherin and increases E-cadherin levels. Lentivirus encoding control or shATR was used to infect MDA-MB-231 cells, and the cells were seeded in 6-well plates with different stiffnesses. E- and N-cadherin levels were then assessed. (**B**) Statistical analysis of the Western blot results in **A** was performed using 2-way ANOVA (*n* = 3). (**C**) MDA-MB-436 cells were infected with lentivirus encoding either control or shATR, and cells were seeded in 6-well plates and analyzed for E- and N-cadherin expression. (**D**) Statistical analysis of the Western blot results in **C** performed using 2-way ANOVA (*n* = 3). (**E**) ATR inhibition decreases N-cadherin and increases E-cadherin expression. MDA-MB-231 cells were treated with DMSO or VX970 (80 nM) and seeded on substrates of varying stiffness, after which E- and N-cadherin levels were assessed. (**F**) Statistical analysis of the Western blot results in **E** performed using 2-way ANOVA (*n* = 3). (**G**) MDA-MB-436 cells were treated with DMSO or VX970 (80 nM), seeded on substrates of varying stiffness, and subsequently harvested for analysis of E-cadherin and N-cadherin expression. (**H**) Statistical analysis of the Western blot results in **G** by 2-way ANOVA (*n* = 3). All statistical data represent biological replicates, and results are expressed as mean ± SD. Western blot and co-IP experiments were independently repeated at least 3 times with similar results.

The process of EMT equips cancer cells with migratory and invasive capabilities and also facilitates their detachment and dissemination to adjacent tissues and distant organs (42, 43). Thus, we next evaluated the effects of ECM stiffness, USP-21, and ATR on the expression of E-cadherin and N-cadherin, markers of epithelial and mesenchymal phenotypes, respectively. Increased ECM stiffness promoted EMT, consistent with prior studies (44). However, ATR inhibition or ATR knockdown increased E-cadherin and decreased N-cadherin expression in MDA-MB-231, MDA-MB-436, and MDA-MB-468 TNBC cells, consistent with a more epithelial phenotype (Figure 4, A–H, and Supplemental Figure 3, A and B). 

**Figure 5:**
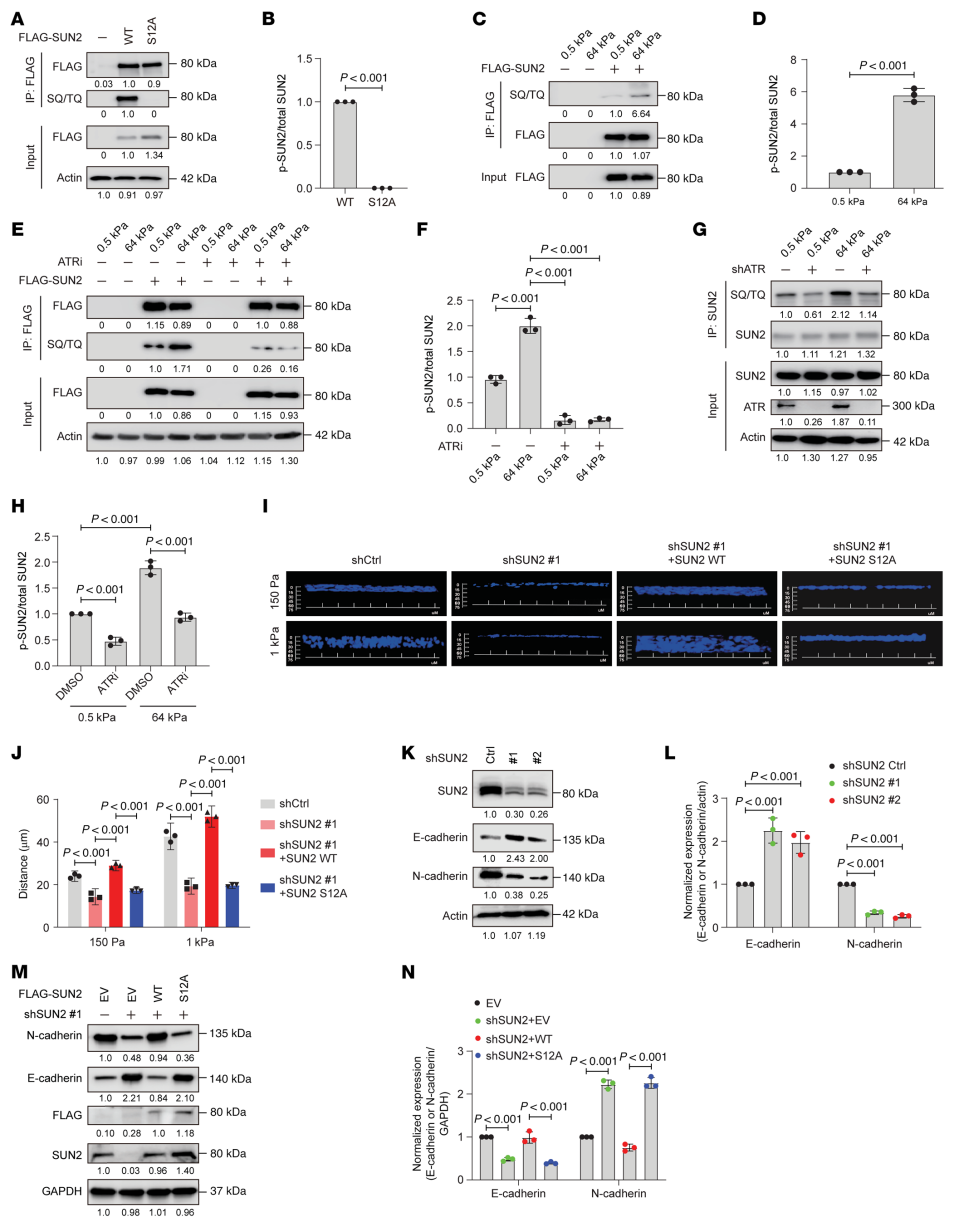
ATR induces EMT by phosphorylating SUN2. (**A** and **B**) ATR phosphorylates SUN2 at S12. MDA-MB-231 cells expressing empty vector, FLAG-SUN2 WT, or FLAG-SUN2 S12A were subjected to FLAG immunoprecipitation, followed by immunoblotting with a phospho-(Ser/Thr) ATM/ATR substrate antibody. Quantification was performed using a 2-tailed Student’s *t* test (*n* = 3). (**C** and **D**) Matrix stiffness promotes SUN2 phosphorylation. Cells expressing empty vector or FLAG-SUN2 WT were cultured on substrates of different stiffnesses for 24 hours, followed by FLAG immunoprecipitation and SUN2 phosphorylation analysis. Statistical significance was determined using 2-tailed Student’s *t* test (*n* = 3). (**E**–**H**) ATR inhibition or knockdown suppresses stiffness-induced SUN2 phosphorylation. SUN2-overexpressing cells cultured on substrates of different stiffnesses were treated with DMSO or VX970 for 18 hours (**E** and **F**) or infected with control or ATR-targeting shRNA (**G** and **H**). SUN2 phosphorylation was assessed following FLAG or SUN2 immunoprecipitation. Data were analyzed by 2-way ANOVA (*n* = 3). (**I** and **J**) SUN2 S12 phosphorylation is required for MDA-MB-231 invasion. Control, SUN2-knockdown, and SUN2-knockdown cells reconstituted with SUN2 WT or S12A were cultured for 3 days on collagen type I gels of different stiffnesses (150 Pa and 1 kPa). Invasion depth was measured by DAPI staining and analyzed by 2-way ANOVA (*n* = 3). (**K** and **L**) SUN2 knockdown decreases N-cadherin and increases E-cadherin expression. Protein levels were assessed by immunoblotting and analyzed by 2-way ANOVA (*n* = 3). (**M** and **N**) Reexpression of SUN2 WT, but not S12A, rescues the changes in E- and N-cadherin caused by SUN2 knockdown. Quantification was performed using 2-way ANOVA (*n* = 3). Data are presented as mean ± SD.

SUN2 is a member of the SUN-domain protein family that forms the LINC complex together with KASH-domain proteins (45). SUN proteins localize to the inner nuclear membrane and interact with the nuclear lamina, whereas KASH proteins are located on the outer nuclear membrane and connect to actin, intermediate filaments, and microtubules (46). Disruption of LINC complex composition has been shown to impair cell migration and EMT (47–49), and a prior mass spectrometry–based analysis of UV-induced ATM/ATR signaling identified SUN2 Ser12 as a potential ATR phosphorylation site (50). To further evaluate SUN2 as an ATR substrate, we overexpressed FLAG-tagged WT SUN2 or an S12A mutant SUN2 in MDA-MB-231 cells under normal culture conditions. Following FLAG IP, we observed robust phosphorylation of WT SUN2 at SQ/TQ motifs, the consensus sites for ATM/ATR kinases (Figure 5, A and B). This phosphorylation was abolished by the S12A mutation, suggesting that S12 is the major ATR target site on SUN2 (Figure 5, A and B, and Supplemental Figure 3, C and D). Notably, SUN2 phosphorylation was detectable under unstressed conditions (Figure 5, A and B, and Supplemental Figure 3, C and D), suggesting that this modification occurs constitutively. However, SUN2 phosphorylation was further enhanced when TNBC cells were grown on stiff surfaces (Figure 5, C and D, and Supplemental Figure 3, E and F). Based on these data, we hypothesized that ATR mediates the response of breast cancer cells to ECM stiffness through SUN2. Consistent with this model, stiffness-induced SUN2 phosphorylation was suppressed by ATR knockdown or pharmacologic ATR inhibition (Figure 5, E–H). Moreover, SUN2 knockdown impaired the invasive capacity of MDA-MB-231 cells and was associated with decreased N-cadherin and increased E-cadherin expression (Figure 5, I–L, and Supplemental Figure 3, G and H). These effects were rescued by reexpression of WT SUN2, but not by the phosphorylation-deficient S12A mutant (Figure 5, I, J, M, and N). Of note, treatment with the ATM inhibitor (ATMi) AZD0156 caused only a slight decrease in SUN2 phosphorylation under high-stiffness conditions, whereas inhibition of DNA-PK with NU7441 had no effect (Supplemental Figure 3, I–L), indicating that SUN2 is primarily an ATR substrate. Interestingly, both AZD0156 and NU7441 reduced invasion through collagen matrices in serum-free medium, potentially via alternative signaling pathways (Supplemental Figure 2A), whereas inhibition of downstream ATR DNA damage response effectors using a dual Chk1/Chk2 inhibitor had no effect (Supplemental Figure 3, M and N). In contrast, hydroxyurea (1 mM) increased cell invasion, consistent with prior reports that DNA damage can promote invasive behavior (Supplemental Figure 2A) (51). Importantly, we observed that combined ATRi and ATMi treatment further reduced invasion, suggesting that the proinvasive effect of ATR inhibition is at least partially mediated through functions independent of the DNA damage response pathway (Supplemental Figure 2B). Collectively, these findings suggested that ATR phosphorylates SUN2 in response to increased ECM stiffness, thereby promoting a more aggressive, mesenchymal phenotype.

**Figure 6:**
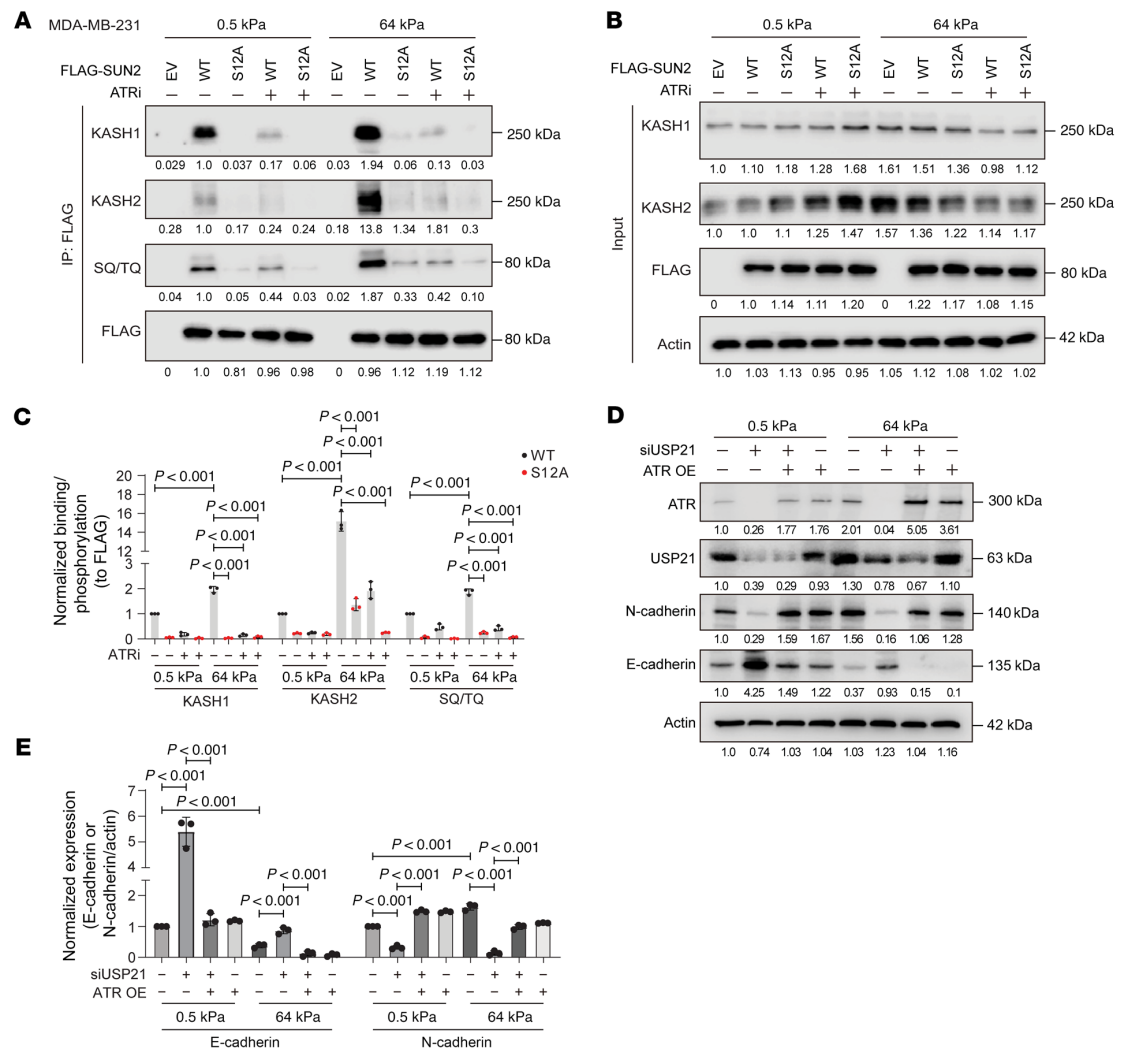
ATR induces EMT by remodeling the LINC complex. (**A** and **B**) SUN2 phosphorylation at S12 mediates its interaction with KASH proteins. MDA-MB-231 cells infected with lentivirus encoding control vector, FLAG-tagged WT SUN2, or FLAG-tagged S12A mutant SUN2 were cultured on 0.5 and 64 kPa surfaces and treated with or without VX970 for 24 hours. FLAG antibody was used for IP, and the binding affinity of SUN2 with KASH1 and KASH2 was assessed by Western blot. All statistical data represent biological replicates, and results are expressed as mean ± SD. Western blot and co-IP experiments were independently repeated at least 3 times with similar results. (**C**) Statistical analysis of the Western blot results in **A** and **B** by 2-way ANOVA (*n* = 3). (**D**) Control, USP21-knockdown, USP21-knockdown plus ATR-overexpressing, or ATR-overexpressing MDA-MB-231 cells were cultured on surfaces with different stiffnesses, and E- and N-cadherin expression was analyzed. (**E**) Statistical analysis of the Western blot results in **D** by 2-way ANOVA (*n* = 3). All statistical data represent biological replicates, and results are expressed as mean ± SD. Western blot and co-IP experiments were independently repeated at least 3 times with similar results.

We next explored the impact of ATR on the LINC complex by examining the interaction between SUN2 with KASH proteins. FLAG-tagged WT SUN2 or the S12A mutant was overexpressed in MDA-MB-231 and MDA-MB-436 cells cultured on surfaces of varying stiffness followed by FLAG IP to assess SUN2 binding to KASH1 and KASH2. Increased ECM stiffness enhanced the interaction of SUN2 with KASH1 and KASH2 (Figure 6, A–C, and Supplemental Figure 3, O and P). In contrast, either the S12A mutation or ATRi treatment significantly reduced these interactions, with ATRi exerting little to no additional effect in cells expressing the S12A mutant (Figure 6, A–C, and Supplemental Figure 3, O and P). In addition, USP21 knockdown similarly reduced N-cadherin expression and increased E-cadherin expression, and these effects were reversed by ATR overexpression (Figure 6, D and E). We concluded from this series of experiments that ATR-mediated phosphorylation of SUN2 is important for LINC complex protein interactions and that a USP21/ATR/SUN2 axis may regulate the EMT process in response to ECM stiffness.

### SUN2 phosphorylation by ATR promotes nuclear localization of β-catenin to drive EMT.

**Figure 7:**
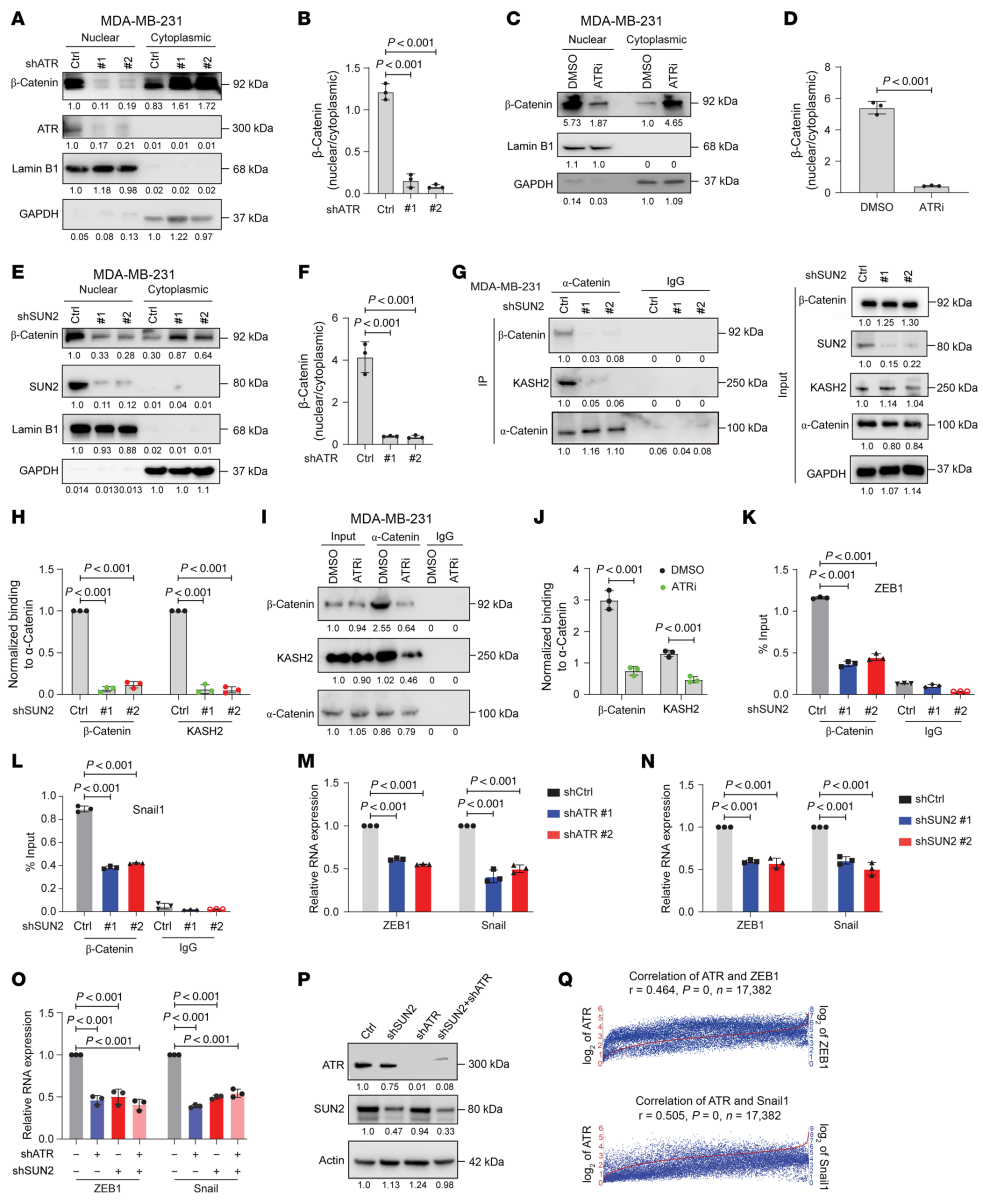
The phosphorylation of SUN2 by ATR facilitates the nuclear translocation of β-catenin. (**A**–**F**) ATR knockdown, ATR inhibition with VX970, or SUN2 knockdown reduces β-catenin nuclear localization in MDA-MB-231 cells, as determined by nuclear-cytoplasmic fractionation and immunoblotting. Quantification was performed using 1-way ANOVA or Student’s *t* test (*n* = 3). (**G**–**J**) SUN2 knockdown or ATR inhibition reduces the association of α-catenin with β-catenin and KASH2. Protein interactions were assessed by α-catenin immunoprecipitation followed by immunoblotting. Data were analyzed by 2-way ANOVA (*n* = 3). (**K** and **L**) SUN2 knockdown decreases β-catenin recruitment to the ZEB1 and SNAIL1 promoter regions, as determined by ChIP-qPCR. Statistical significance was assessed using 1-way ANOVA (*n* = 3). (**M**) ATR knockdown reduces the expression level of ZEB1 and Snail1. MDA-MB-231 cells infected with lentivirus encoding control or shATR were harvested, and the mRNA levels of ZEB1 and Snail were detected by RT-qPCR (*n* = 3). Statistical analysis was performed using 1-way ANOVA. (**N**) SUN2 knockdown reduces the expression level of ZEB1 and Snail1. MDA-MB-231 cells infected with lentivirus encoding control or shSUN2 were harvested, and the mRNA level of ZEB1 and Snail was detected using RT-qPCR (*n* = 3). Statistical analysis was performed using 1-way ANOVA. (**O** and **P**) MDA-MB-231 cells infected with lentiviruses encoding control, shSUN2, shATR, or both shSUN2 and shATR were harvested for RNA extraction, and the expression of ZEB1 and Snail was subsequently assessed. Statistical analysis was performed using 1-way ANOVA. (**Q**) ATR expression level is positively correlated with ZEB1 and Snail1. The data were extracted from the TCGA database. All statistical data represent biological replicates, and results are expressed as mean ± SD. Western blot and co-IP experiments were independently repeated at least 3 times with similar results.

β-Catenin interacts with E-cadherin at the cell membrane to form adherens junctions that maintain the epithelial phenotype. Disruption of this interaction allows β-catenin to translocate into the nucleus, where it promotes EMT (52). Previous studies have shown that KASH proteins within the LINC complex regulate EMT by modulating β-catenin nuclear localization through interactions with α-catenin (14, 15). Once in the nucleus, β-catenin functions as a transcriptional coactivator to induce expression of genes associated with a mesenchymal phenotype (53). Based on these observations, we investigated whether ATR and SUN2 regulate β-catenin localization. In control cells, β-catenin exhibited prominent nuclear localization. In contrast, ATR inhibition or knockdown of ATR or SUN2 resulted in redistribution of β-catenin to the cytoplasm in MDA-MB-231 and MDA-MB-436 cells (Figure 7, A–F, and Supplemental Figure 4A). Moreover, SUN2 knockdown, ATR knockdown, and ATR inhibition reduced the interaction of β-catenin with α-catenin and KASH2 (Figure 7, G–J).

Consistent with the reduced nuclear localization of β-catenin following loss of ATR or SUN2, β-catenin occupancy at the promoters of its EMT-associated target genes ZEB1 and SNAI1 was also diminished under these conditions (Figure 7, K and L, and Supplemental Figure 4B), resulting in decreased expression of both ZEB1 and SNAI1 expression (Figure 7, M and N, and Supplemental Figure 4, C and D). Combined knockdown of ATR and SUN2 did not further reduce ZEB1 and SNAI1 levels, suggesting that ATR and SUN2 function in the same signaling pathway (Figure 7, O and P). Furthermore, analysis of normal tissue samples from The Cancer Genome Atlas (TCGA) database revealed a significant positive correlation between ATR expression and the expression of ZEB1 (*r* = 0.464, *P* = 0) and SNAI1 (*r* = 0.505, *P* = 0) (Figure 7Q). Collectively, these results support a model in which ATR promotes β-catenin nuclear localization and EMT by modulating LINC complex composition through SUN2 phosphorylation.

### Inhibiting ATR suppresses metastasis and immunosuppression by reversing EMT.

**Figure 8:**
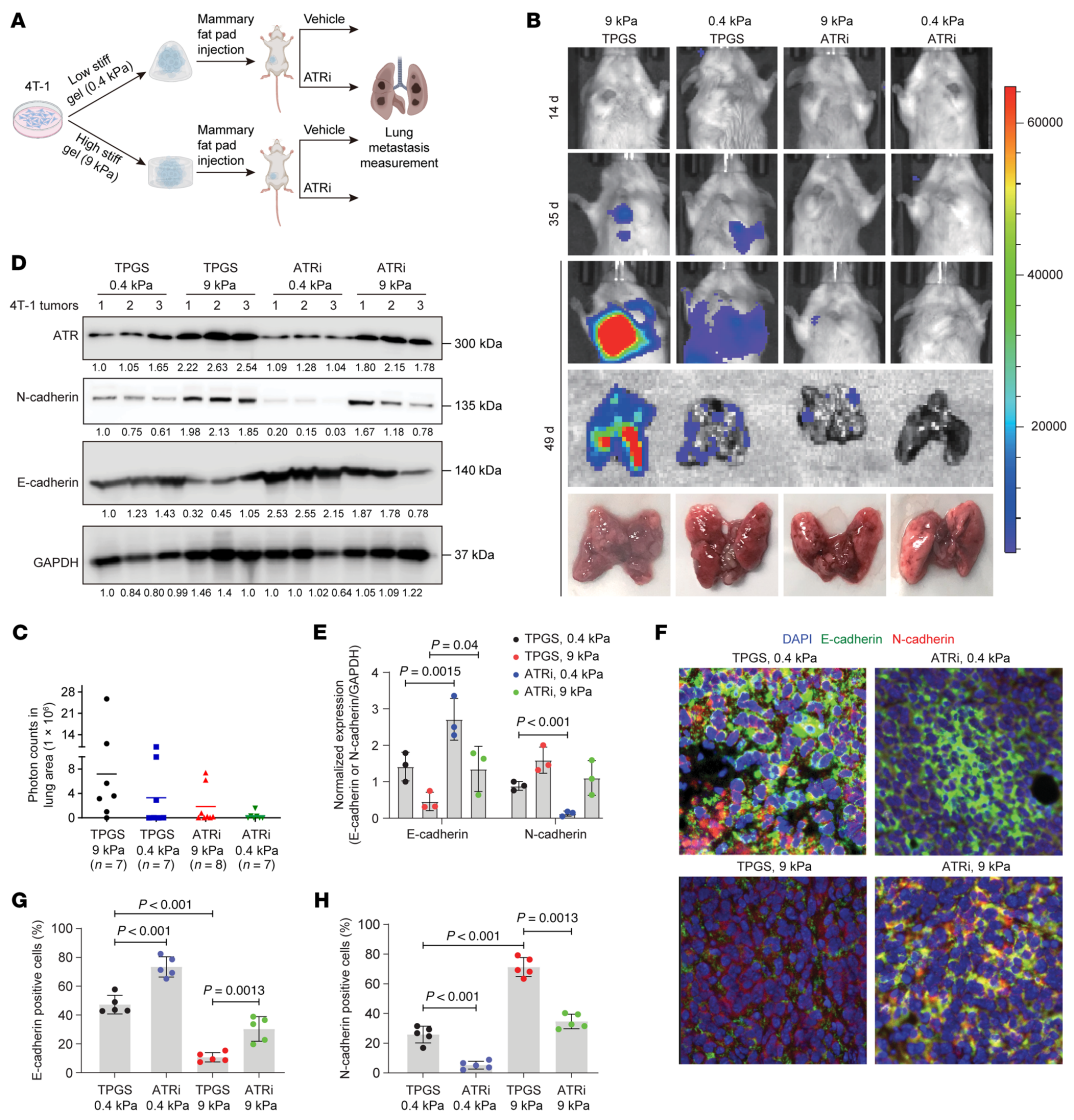
ATR inhibition suppresses metastasis. (**A**) Schematic of the 4T1 model to determine the role of ATR in metastasis. 4T1 cells were initially coated with low- (0.4 kPa) or high-stiffness (9 kPa) HA gel and injected into the mammary fat pad of BALB/c mice. The mice were subsequently treated with either vehicle or VX970 (60 mg/kg), and the effect of ATR inhibition on antitumor immunity and metastasis was assessed. (**B** and **C**) Quantified lung metastasis photon counts in all groups of mice. (**D**) The expression of E- and N-cadherin in tumor tissues harvested from **A** was detected by Western blot. (**E**) Statistical analysis of the Western blot results in **D** by 2-way ANOVA (*n* = 3). (**F**–**H**) Tumor tissues from 4T1 primary tumors were harvested, and the levels of E- and N-cadherin were determined using immunofluorescence. The percentage of positive cells per ×40 high-power field is displayed. Quantification from 5 randomly selected fields was used for statistical analysis (*n* = 5). Statistical significance was determined by 1-way ANOVA. Data are presented as mean ± SD.

EMT has been shown to inhibit antitumor immunity, as mesenchymal cells secrete immunosuppressive cytokines and recruit immune cells with immunosuppressive functions (54). These features support immune evasion and may contribute to metastatic progression. We therefore evaluated the effects of ECM stiffness and ATR inhibition on EMT and metastasis in vivo in the immune-competent 4T1 TNBC mouse model. Luciferase-expressing 4T1 cells were coated with low- (0.4 kPa) or high-stiffness (9 kPa) hyaluronic acid (HA) gels prior to injection into the mammary fat pad, modeling distinct ECM stiffness conditions within the tumor microenvironment, as previously described (Figure 8A) (55). These stiffness values approximate those reported for normal mammary tissue and breast tumors (25–31). Consistent with our prior studies demonstrating limited single-agent activity (41), ATR inhibition did not significantly affect primary tumor volume at the injection sites. In addition, primary tumor size did not differ between low- and high-stiffness conditions (Supplemental Figure 5A). In contrast, tumors grown under high-stiffness conditions exhibited a significantly greater lung metastatic burden (Figure 8, B and C). Moreover, despite the lack of single-agent activity at the primary tumor site, ATR inhibition significantly reduced lung metastasis under stiff ECM conditions (Figure 8, B and C). Consistent with our observations in cultured cells (Figure 4, A–D), 4T1 tumors grown in stiffer environments displayed increased N-cadherin and decreased E-cadherin expression (Figure 8, D–H). ATRi treatment reversed this pattern, reducing N-cadherin and increasing E-cadherin levels, indicating that ATR inhibition can counteract the mesenchymal phenotype induced by increased ECM stiffness (Figure 8, D–H).

**Figure 9:**
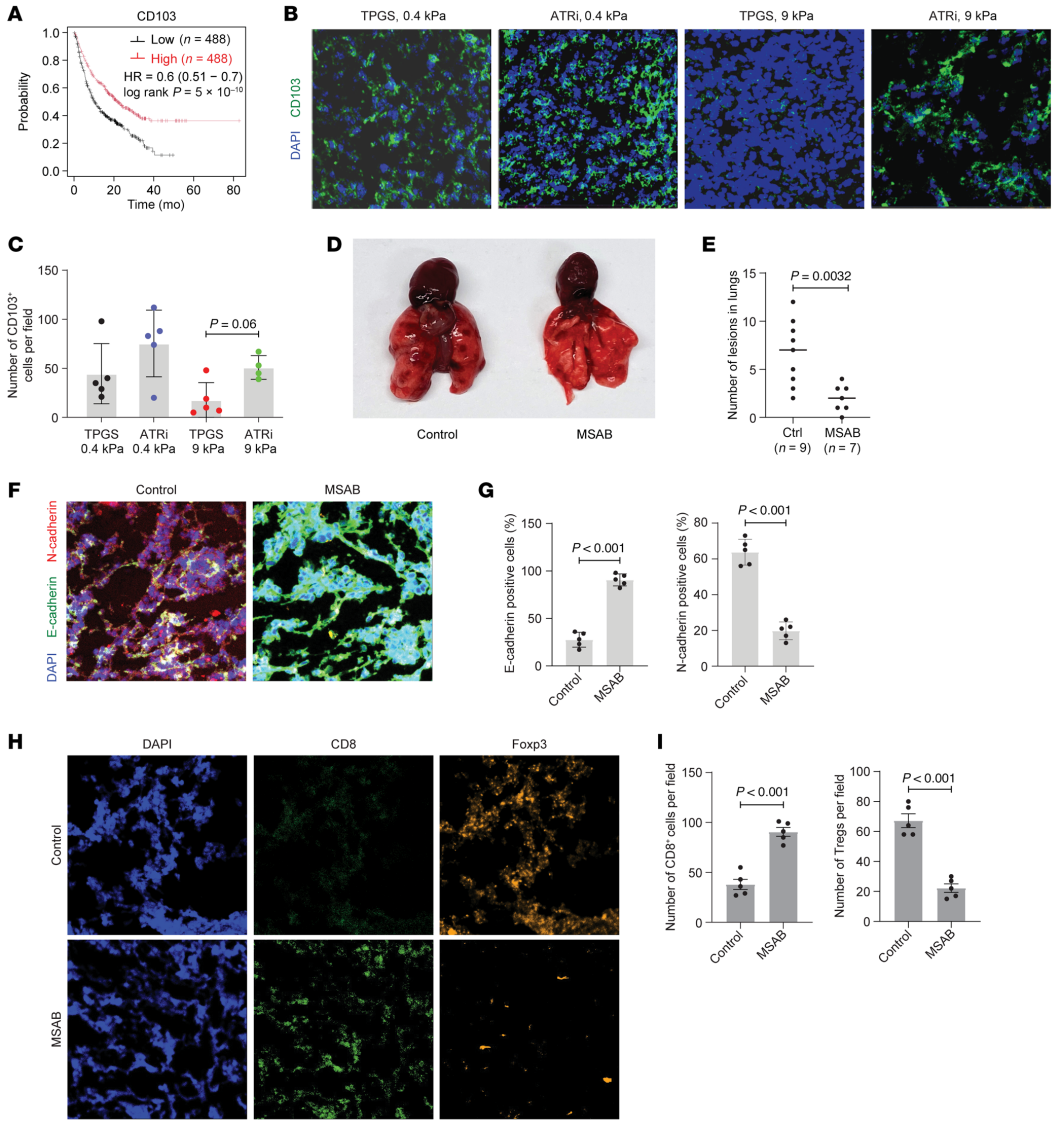
ATR inhibition promotes antitumor immunity. (**A**) CD103 cells were positively correlated with overall survival of patients treated with immunotherapy. This result was obtained using the Kaplan-Meier plotter, with the median CD103 expression level used as the cutoff to divide patients into high- and low-expression groups. (**B** and **C**) The 4T1 tumor tissues from Figure 1D were collected, and the levels of CD103^+^ cells were evaluated using IHC. Quantification from 5 randomly selected fields was used for statistical analysis (*n* = 5). All statistical data represent biological replicates, and results are expressed as mean ± SD. Statistical significance was determined using 1-way ANOVA. (**D** and **E**) 3 × 10^4^ 4T1 cells were injected into the mammary fat pad of BALB/c mouse models. Mice were treated daily for 2 weeks with either vehicle (corn oil) or the β-catenin inhibitor MSAB (10 mg/kg), after which lung metastasis was evaluated. Statistical analysis was performed using 2-tailed Student’s *t* test. (**F** and **G**) Tumors harvested from Figure 8A were evaluated for the expression of E- and N-cadherin (*n* = 5). (**H** and **I**) Tumors harvested from Figure 8A were evaluated for CD8^+^ T cell and Treg cell infiltration (*n* = 5). Statistical analysis was performed using 2-tailed Student’s *t* test. Original magnification for all micrographs, ×40.

E-cadherin interacts with CD103, facilitating the recruitment, retention, and functional activity of CD103^+^ immune cells within tumors. These cells have been associated with improved responsiveness to immunotherapy and favorable clinical outcomes (56, 57). Consistent with this, analysis of a publicly available dataset revealed a positive correlation between intratumoral CD103^+^ cell abundance and patient survival following immunotherapy (Figure 9A). We therefore examined the effects of ECM stiffness and ATR inhibition on CD103^+^ immune cells and other immune populations within 4T1 tumors. Tumors grown under stiff ECM conditions exhibited features of an immunosuppressed tumor microenvironment, including reduced numbers of CD103^+^ cells, CD11c^+^ dendritic cells, and CD4^+^ and CD8^+^ T lymphocytes, along with increased Foxp3^+^ Tregs (Supplemental Figure 5, B–G). Notably, ATRi treatment significantly increased the abundance of CD103^+^ cells within the tumor microenvironment (Figure 9, B and C). In addition, ATR inhibition increased dendritic cells and CD4^+^ and CD8^+^ lymphocytes while reducing immunosuppressive Tregs (Supplemental Figure 5, B–G), indicating enhanced antitumor immune activity.

Given that ATR inhibition regulates EMT by promoting the nuclear localization of β-catenin, we next examined whether pharmacologic inhibition of β-catenin using MSAB similarly suppresses metastasis and enhances antitumor immunity in the 4T1 model. Consistent with this hypothesis, MSAB treatment significantly reduced lung metastasis (Figure 9, D and E). Immunofluorescence analysis demonstrated that MSAB reversed the EMT phenotype, as evidenced by decreased N-cadherin and increased E-cadherin expression (Figure 9, F and G). Moreover, MSAB treatment increased tumor-infiltrating CD8^+^ T cells while reducing Tregs, closely mirroring the immune effects observed following ATR inhibition (Figure 9, H and I).

Collectively, these results suggested that ECM stiffness and ATR contribute to immunosuppression in TNBC and suggest that ATR inhibition may reverse stiffness-associated immune suppression by promoting a mesenchymal-to-epithelial transition.

### Inhibition of ATR enhances immunotherapy response in stiff tumors and promotes CD103^+^ dendritic cell recruitment.

**Figure 10:**
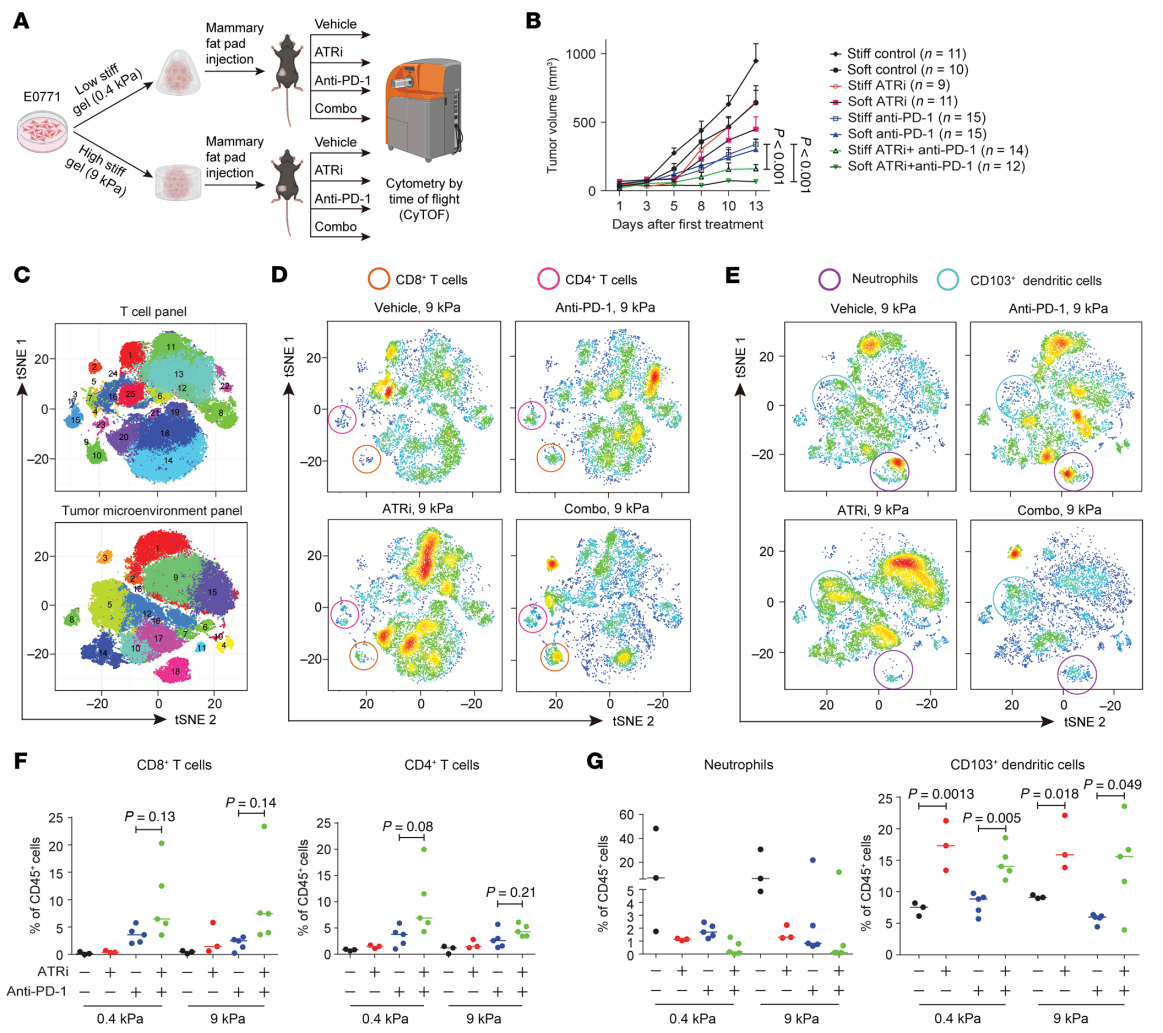
ATRi treatment sensitizes tumors to immunotherapy by remodeling the tumor immune microenvironment. (**A**) Schematic of the E0771 model to determine the role of ATR in antitumor immunity. E0771 cells were initially coated with low- (0.4 kPa) or high-stiffness (9 kPa) HA gel and injected into the mammary fat pads of C57BL/6J mice. The mice were subsequently treated with either a vehicle, VX970 (60 mg/kg), an anti-PD-1 antibody, or a combination treatment. The tumors were then harvested for CyTOF analysis. (**B**) The growth curves of tumors under different treatments are presented. High stiffness promoted tumor growth, while ATRi sensitized tumors to immunotherapy. (**C**) The tSNE plots for T cells and the tumor microenvironment panel for high-stiffness tumors are displayed. (**D**–**G**) The quantification of CD8^+^, CD4^+^, neutrophils, and CD103^+^ dendritic cells in tumors from different groups. ATR inhibition increased CD8^+^, CD4^+^, and CD103^+^ dendritic cells in tumors and decreased neutrophil cells in stiff tumors. Statistical analysis was performed by 1-way ANOVA (*n* = 3).

We next examined how ECM stiffness and ATRi treatment affect immunotherapy responsiveness in the E0771/C57BL/6 TNBC model. Because E0771 tumors exhibit low metastatic potential, this model is well suited for assessing local tumor responses to immunotherapeutic interventions (Figure 10A). Mice bearing mammary fat pad tumors established in either low- or high-stiffness ECM were randomly assigned to receive either vehicle, ATRi, anti–PD-1, or the combination of ATRi and anti–PD-1. Untreated tumors grown under high-stiffness conditions displayed the most rapid growth, whereas combining ATRi and anti–PD-1 treatment resulted in the greatest delay in tumor progression (Figure 10B). Consistent with our findings in the 4T1 model and in human cell lines studied in vitro, E0771 tumors grown in stiff matrices exhibited increased N-cadherin expression and reduced E-cadherin levels. Notably, ATR inhibition reversed these stiffness-induced EMT-associated changes (Supplemental Figure 6, A and B).

To further investigate how ATR inhibition might remodel the immune microenvironment within stiff tumors in the context of immunotherapy, we isolated tumor-infiltrating immune cells and performed time-of-flight mass cytometry (CyTOF). Analysis using a T cell–focused panel identified 25 clusters, while a tumor microenvironment panel resolved 19 distinct clusters (Figure 10C and Supplemental Figure 6C). ATRi monotherapy increased CD103^+^ dendritic cells as well as CD4^+^ and CD8^+^ T cells, while reducing immunosuppressive Ly6G^+^ neutrophils (Figure 10, D–G). Anti–PD-1 treatment also increased CD8^+^ and CD4^+^ T cell populations (Figure 10, D and F, and Supplemental Figure 6D). Notably, combined ATRi and anti–PD-1 treatment further amplified CD4^+^ and CD8^+^ T cell infiltration, significantly increased CD103^+^ dendritic cells, and markedly reduced neutrophil accumulation, consistent with enhanced immune activation (Figure 10, D–G, and Supplemental Figure 6D). In addition, B cells and Ly6C^+^ CD8^+^ T cells were significantly increased in the combination treatment group compared with anti–PD-1 monotherapy (Supplemental Figure 6, F and H). Ly6C^+^ CD8^+^ T cells represent a functionally active subset enriched in inflammatory and tumor-draining environments and are associated with heightened effector potential and stronger antitumor responses (58, 59).

Consistent with prior reports that immune checkpoint blockade can expand both effector T cells and Tregs (60), anti–PD-1 monotherapy increased Treg frequencies. Importantly, ATR inhibition attenuated this PD-1–associated Treg expansion, resulting in a more favorable effector to regulatory immune balance (Supplemental Figure 6, F–H).

### ATR suppresses CD103^+^ dendritic cell recruitment and promotes PMN myeloid-derived suppressor cell infiltration through EMT.

**Figure 11:**
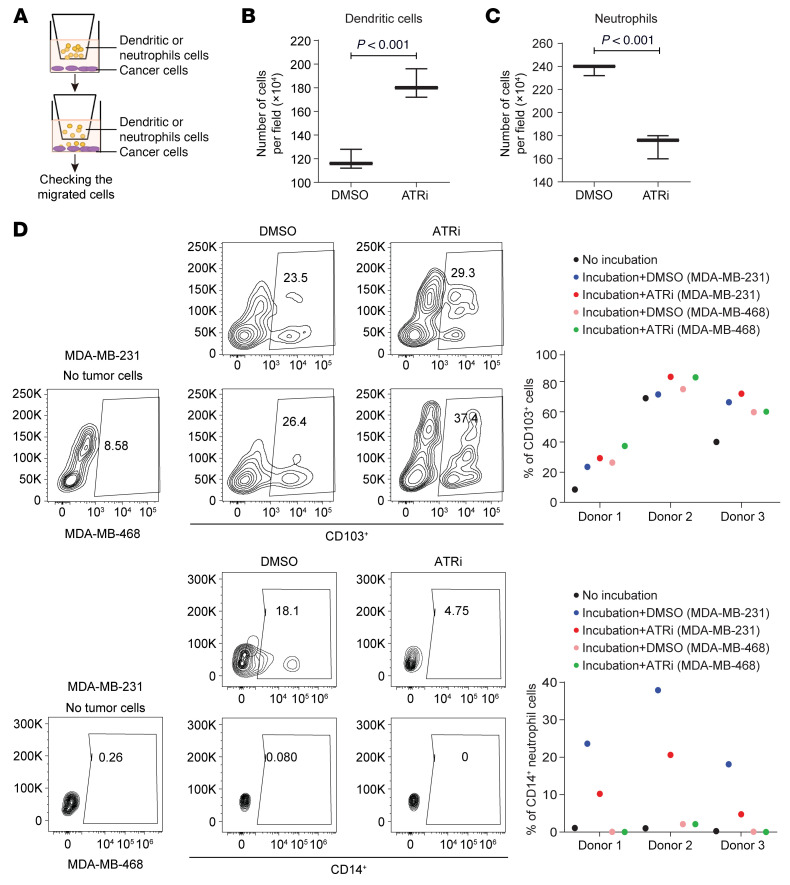
ATR inhibits the recruitment of CD103^+^ dendritic cells and promotes the infiltration of MDSCs. (**A**) 2 × 10^5^ MDA-MB-231 cells were cultured on the bottom of 24-well plates and treated with or without VX970 (80 nM) for 24 hours, while 1 × 10^7^ dendritic or neutrophil cells were isolated from PBMCs of healthy donors and seeded on the transwell inserts. The migrated immune cells were measured 8 hours later. (**B** and **C**) Statistical analysis of data from **A**. MDA-MB-231 cells pretreated with ATR inhibition increased CD103 cell recruitment and decreased neutrophil cell migration. Statistical significance was determined by 2-tailed Student’s *t* test. Data are presented as mean ± SD. (**D**) MDA-MB-231 and MDA-MB-468 cells were pretreated with VX970 (80 nM) for 24 hours, followed by incubation with PBMCs from healthy donors. The percentage of CD103^+^ dendritic cells and neutrophil cells was then measured.

To further examine how ATR inhibition influences cancer cell–mediated recruitment of dendritic cells and neutrophils, we performed ex vivo transwell migration assays using immune cells isolated from healthy human donors (Figure 11A). In alignment with our findings in the E0771 model, ATRi-treated MDA-MB-231 cells promoted increased dendritic cell migration while significantly reducing neutrophil migration compared with vehicle-treated controls (Figure 11, B and C).

**Figure 12:**
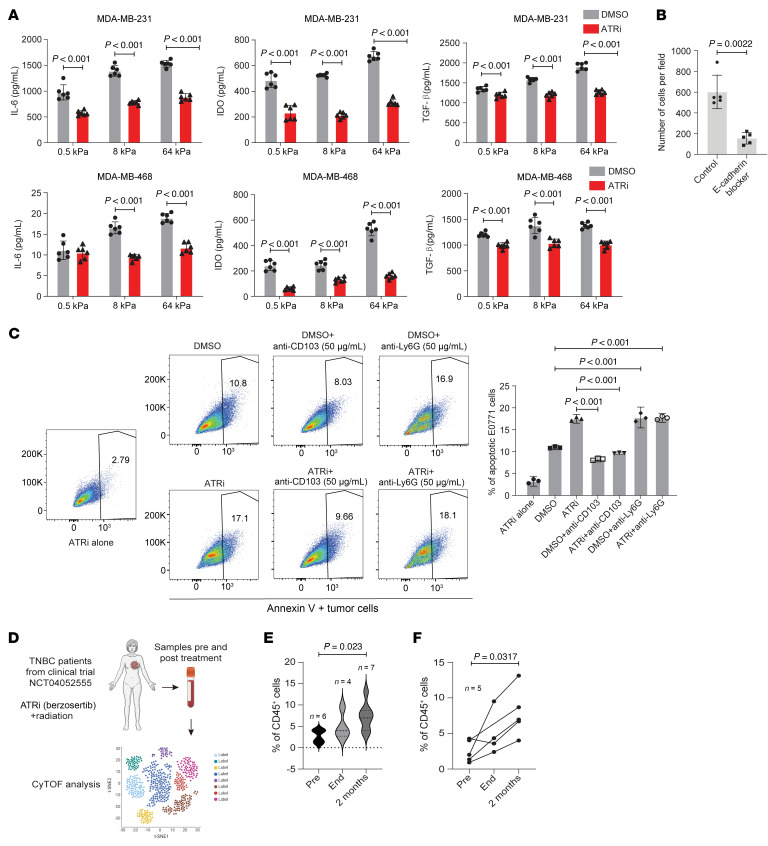
ATR inhibition enhances cytokine production and promotes antitumor immune responses. (**A**) MDA-MB-231 and MDA-MB-468 cells were pretreated with VX970 (80 nM) for 24 hours, after which the indicated cytokines were measured by ELISA (*n* = 6). Statistical analysis of the Western blot results in **E** by 2-way ANOVA (*n* = 3). (**B**) 2 × 10^5^ MDA-MB-231 tumor cells were seeded in the lower chamber of 24-well plates. After a 30-minute pretreatment in calcium-free DMEM to disrupt existing junctions, we treated the tumor cells with 5 μg/mL of either nonspecific IgG or E-cadherin blocking antibody for 48 hours. 1 × 10^4^ CD103^+^ cells, freshly isolated from healthy donor PBMCs, were placed in the upper chamber of the transwell insert. After 8 hours of migration, we counted the number of CD103^+^ cells that had traversed into the lower chamber. Statistical significance was determined by 2-tailed Student’s *t* test. (**C**) E0771 cells were pretreated with VX970 for 24 hours, followed by incubation with PBMCs under the indicated treatment. The apoptosis of E0771 was then measured by the percentage of annexin V^+^ cells (*n* = 3). Statistical analysis was performed by 1-way ANOVA (*n* = 3). (**D**–**F**) Enhanced levels of HLA-DR^+^ dendritic cells and reduced Tregs were observed in the peripheral blood of patients following ATRi treatment. All statistical data represent biological replicates, and results are expressed as mean ± SD. Statistical analysis was performed by 1-way ANOVA.

The EMT has been linked to enhanced infiltration of myeloid-derived suppressor cells (MDSCs) in TNBC (61). In TNBC, the majority of tumor-infiltrating neutrophils exhibit immunosuppressive properties and phenotypically correspond to PMN-MDSCs, which promote tumor progression and immune evasion (62). Unlike classic neutrophils, PMN-MDSCs are characterized by high expression of CD14^+^, a marker that is typically absent on conventional peripheral-blood neutrophils but may become aberrantly upregulated upon exposure to the tumor microenvironment (63, 64). Consistent with this paradigm, incubation of healthy donor PBMCs with MDA-MB-231 cells resulted in a marked increase in CD14^+^ neutrophils. Importantly, this effect was significantly attenuated when tumor cells were pretreated with ATRi (Figure 11D). We also observed that ATR inhibition reduced the secretion of mesenchyme-associated cytokines, including IL-6, IDO, and TGF-β, in both MDA-MB-231 and MDA-MB-468 cells (Figure 12A), factors known to promote MDSC recruitment and maintenance within the tumor microenvironment (54, 65, 66). Incubation of PBMCs with MDA-MB-468 cells did not increase the proportion of CD14^+^ neutrophils (Figure 11D), potentially reflecting the more epithelial phenotype of this cell line (67), as evidenced by higher E-cadherin expression (Figure 4, A–D).

In addition, ATR inhibition increased the proportion of dendritic cells expressing CD103^+^ (Figure 11D). CD103^+^ immune cells interact with epithelial tumor cells through binding to E-cadherin, a process that enhances their retention within tumors. Consistent with this mechanism, blockade of E-cadherin markedly reduced CD103^+^ cell migration toward tumor cells in the transwell assay (Figure 12B). Functionally, depletion of CD103^+^ cells from PBMCs impaired immune cell–mediated cytotoxicity following ATR inhibition in TNBC cells, whereas removal of neutrophils enhanced cell killing, underscoring the opposing roles of these immune populations in shaping antitumor responses to ATR inhibition (Figure 12C).

Finally, we explored whether ATR inhibition alters circulating antigen-presenting dendritic cells in patients treated with the ATRi berzosertib. Peripheral blood specimens were analyzed from a subset of patients enrolled in the National Cancer Institute 10291 (NCI10291) trial, a dose-escalation study of berzosertib in combination with standard-of-care locoregional therapy for therapeutically resistant early-stage breast cancer (ClinicalTrials.gov NCT04052555) (Figure 12D). While cryopreserved specimens were not suitable for neutrophil assessment, we observed a significant increase in the ratio of HLA-DR^+^ antigen-presenting dendritic cells in posttreatment samples compared to pretreatment samples (Figure 12E). Notably, all 5 patients with paired pre- and posttreatment specimens demonstrated increased HLA-DR^+^ dendritic cells following therapy (Figure 12F), supporting a clinically important role for ATR inhibition in enhancing antigen presentation and cytotoxic T cell priming (68).

## Discussion

Our findings identify a noncanonical ATR/SUN2 signaling axis that links ECM stiffness to β-catenin activation, EMT, immune evasion, and metastasis. ATR inhibition reversed these effects and enhanced anti–PD-1 responsiveness, highlighting ATR as a potential therapeutic target in stiffness-driven tumors.

Prior studies have linked DNA damage and replication stress to EMT through H2AX-, ALC1-, and ATR/ZEB1-dependent mechanisms (69–72). In contrast, our findings show that ATR activation under stiff ECM conditions can occur independently of detectable DNA damage, revealing a noncanonical, mechanotransduction-based mode of ATR engagement. By identifying SUN2 and the LINC complex as mediators of this process, our study extends the traditional DNA damage response–EMT framework and uncovers an ATR-regulated axis of nuclear-cytoskeletal communication.

Beyond its established role in DNA repair (73), USP21 is repurposed under stiff ECM conditions to stabilize ATR and drive DNA damage–independent mechanotransductive signaling that promotes malignant phenotypes.

Given the established association between CD103^+^ tumor-infiltrating immune cells, E-cadherin–mediated tumor retention, and improved immunotherapy responses (7, 74–78), our finding that ATR inhibition increased CD103^+^ immune cell infiltration while restoring E-cadherin expression suggests a shift toward a more immune-inflamed, therapy-responsive state. ATR inhibition also reduced immunosuppressive CD14^+^ neutrophils, supporting a role for ATR in coupling ECM stiffness to EMT-associated immune suppression.

Despite its strong effects on metastasis and immune suppression, ATR inhibition had only modest effects on primary tumor growth in the highly metastatic 4T1 model, whereas it significantly reduced tumor burden in the less metastatic E0771 model. These differences may reflect tumor-specific context, including ECM stiffness, drug delivery constraints, and compensatory survival pathways. Increased matrix stiffness can compress intratumoral vasculature and impair perfusion, oxygenation, and drug delivery (79, 80), while alternative DNA damage response and oncogenic signaling pathways may partially sustain tumor growth following ATR inhibition (81–87). Together, these factors likely influence tumor dependency on ATR and therapeutic response.

In summary, our study uncovers a noncanonical, mechanotransduction-based role for ATR in promoting EMT, immune evasion, and metastatic progression in response to ECM stiffness.

## Methods

Additional details may be found in the supplemental materials.

### Sex as a biological variable.

All patient samples were obtained from female patients, as TNBC is a subtype of breast cancer that occurs overwhelmingly in women. Therefore, sex was not treated as a variable in this analysis. Only female mice were used because breast cancer occurs predominantly in women and the models employed orthotopic implantation into the mammary fat pad.

### Cell lines, antibodies, and plasmids.

MDA-MB-231, MDA-MB-436, and 4T1 cells were obtained from ATCC and cultured in DMEM supplemented with 10% FBS.

Primary antibodies included ATR (13934S, Western blot; ab222820, IHC), β-catenin (8480S), E-cadherin (3195S), N-cadherin (13116S), CD4 (25229S), CD8 (98941S), Foxp3 (12653S), CD11c (97585S), β-tubulin (86298S), α-E-catenin (3240S), and SQ/TQ (6966S) from Cell Signaling Technology; GAPDH (PLA0125-100UL), USP21 (HPA028397-100UL), and FLAG (F1804) from Sigma-Aldrich; SUN2 (ab124916), KASH1 (ab192234), and KASH2 (ab224328) from Abcam; UB (sc-8017) and USP21 (sc-79305, IHC) from Santa Cruz Biotechnology; Lamin B (12987-1-AP) from Proteintech; and E-cadherin blocking antibody (14-3249-82) from Invitrogen.

ATR and SUN2 shRNAs were obtained from Sigma-Aldrich (MISSION shRNA, SHCLNG), and USP21 siRNA from Dharmacon (ON-TARGETplus SMARTpool). shRNA and siRNA sequences are as follows: shATR1, CCGGCTCCGTGATGTTGCTTGATTTCTCGAGAAATCAAGCAACATCACGGAGTTTTTG; shATR2, CCGGCTGTGGTTGTATCTGTTCAATCTCGAGATTGAACAGATACAACCACAGTTTTTG; shSUN2-1, CCGGGCCTATTCAGACGTTTCACTTCTCGAGAAGTGAAACGTCTGAATAGGCTTTTTTG; and shSUN2-2, CCGGGTGTATATATGTAGCATATCACTCGAGTGATATGCTACATATATACACTTTTTTG.

For shRNA knockdown, we used a second-generation lentiviral system. Briefly, we cotransfected 293T cells with package plasmids and shRNA (with puro resistance). The cells were then cultured for 48 hours to produce the virus. The media-containing viruses were harvested to perform infection. Infected cells were then screened with puromycin after 72 hours of culture.

For siRNA knockdown, we used DharmaFECT 4 reagent (Dharmacon, Horizon Discovery, NC1411281) and performed the experiment according to the manufacturer’s instructions.

The WT, mutant, and truncated USP21 were cloned into plvx3-puro, generated in house. WT and mutant SUN2 were cloned into plvx3-puro. The firefly luciferase–expressed vector Plx313-firefly luciferase was purchased from Addgene (plasmid 118017) (88).

USP21 siRNA was ordered from Dharmacon (ON-TARGETplus siRNA, LQ-006071-00-0002).

### RNA extraction, reverse transcription, and RT-qPCR.

RNA extraction was performed using the quick-RNA miniPrep kit (Zymo Research) according to the manufacturer’s instructions. Reverse transcription was performed with SuperScript III First-Strand Synthesis SuperMix (Invitrogen). RT-qPCR was performed using SYBR Green PCR Master Mix (Applied Biosystems).

### Co-IP.

Cells were first lysed by NETN buffer (20 mM Tris-HCl, pH 8.0, 150 mM NaCl, 1 mM EDTA, 0.5% Nonidet P-40). 3 �g antibody and protein A/G beads were then added to the cell lysate. After overnight incubation, the beads were washed 3–5 times with NETN. The coimmunoprecipitated protein was then analyzed by Western blot.

### Preparation of collagen and hydrogels.

Collagen gels of approximately 150 Pa and 1 kPa stiffness were prepared according to the manufacturer’s protocol (Advanced BioMatrix). Briefly, chilled collagen solution was mixed with 10× PBS at a 8:1 ratio, neutralized to pH 7.0–7.5 with sterile 0.1 M NaOH, adjusted to final volume with sterile water, and incubated at 37°C to induce gelation. PureCol collagen solution (5005-100ML, Advanced BioMatrix) was used for approximately 150 Pa gels, and Nutragen collagen solution (5010-50ML, Advanced BioMatrix) was used for approximately 1 kPa gels. Cytosoft Discovery Kit plates with defined stiffness values of 0.2, 2, 5, 8, 16, 32, and 64 kPa were purchased from Advanced BioMatrix (catalog 5190).

### Microscopy and 3D invasion analysis.

Immunofluorescence imaging was performed using a Zeiss LSM 780 laser scanning confocal microscope equipped with a 405 nm laser line for DAPI excitation. Cells were visualized using a Plan-Apochromat ×20/0.8 NA objective, maintaining identical acquisition parameters (laser power, detector gain, and pinhole size) across all experimental groups. Images were acquired using ZEN Black software (Zeiss).

*Z*-stack images were acquired at 5–10 μm optical intervals through the full depth of the collagen matrix using ZEN Black acquisition software. 3D reconstructions were generated using ZEN Black (version 2.3), and invasion depth was quantified from the exported 3D volume. The mean invasion depth and invasion index were calculated.

### Immunofluorescence imaging.

Immunofluorescence imaging was performed using a Zeiss LSM 780 laser scanning confocal microscope equipped with 405, 488, 561, and 633 nm laser lines. Cells were visualized using a Plan-Apochromat ×40/1.2 NA water immersion objective, maintaining identical acquisition parameters (laser power, detector gain, and pinhole size) across all experimental groups. All image acquisition and processing were performed using ZEN Black software (version 2.3) with identical settings applied to all conditions.

### Animal model.

Low- (0.4 kPa) and high-stiffness (9 kPa) hyaluronic hydrogels were prepared as previously described (55). For the 4T1 model, 3 × 10^4^ firefly luciferase–expressing 4T1 cells were coated with hydrogels and implanted into the mammary fat pads of BALB/c mice. Mice were treated with TPGS (d-α-tocopheryl polyethylene glycol 1000 succinate) vehicle or berzosertib (60 mg/kg) 3 times weekly for 3 weeks, and metastasis was assessed using an IVIS 200 imaging system (Xenogen).

For the E0771 model, 5 × 10^5^ hydrogel-coated E0771 cells were implanted into the mammary fat pads of C57BL/6 mice. Mice received TPGS, VX970 (60 mg/kg), anti–PD-1 antibody (200 μg/mouse), or the combination 3 times weekly for 3 weeks, and tumor growth was monitored.

For IHC, tumors were harvested, cryosectioned, stained using the Opal 4-color IHC kit (Akoya Biosciences, NEL820001KT), and quantified by confocal microscopy.

### ChIP.

ChIP was performed using a ChIP assay kit (Millipore, 17-295) according to the manufacturer’s instructions.

### Immunofluorescence.

Immunofluorescence analysis was performed as previously described with minor modification (89). Briefly, cells were first fixed by 3% paraformaldehyde, permeabilized with 0.5% Triton X-100, and blocked by 5% goat serum followed by incubating with primary antibody. Fluorescent secondary antibody and DAPI were then incubated with cells to stain the targeted proteins and nucleus. The data were analyzed by fluorescence microscopy.

### TNBC patient specimen collection and data analysis.

Primary tumor and adjacent nontumor tissues were harvested from 34 patients with TNBC at First Affiliated Hospital of Nanchang University and underwent paraffin embedding. Following IHC using the indicated antibodies, the expression of each protein was determined by a pathologist using the immunoreactive score.

### Statistics.

Statistical analyses were performed using 2-tailed Student’s *t* tests. 1- or 2-way ANOVA was used depending on the number of variables being compared, as specified in the figure legends. *P* values of less than 0.05 were considered statistically significant.

### Study approval.

Patients samples were analyzed from the NCI10291 trial, an investigator-initiated, multisite, single-arm, open-label, dose-finding phase Ib clinical trial with the aim of characterizing the safety profile and deriving the recommended phase II dose of berzosertib combined with conventional doses of breast or chest wall and regional nodal irradiation. The study protocol was reviewed and approved by the Mayo Clinic Institutional Review Board. The trial is registered at ClinicalTrials.gov (NCT04052555) and was independently monitored by a data safety and monitoring board. Written informed consent was obtained from all patients prior to enrollment. All animal experiments were performed under a protocol approved by the Mayo Clinic IACUC (protocol no. A00003935-18-R24; approval date, August 30, 2024) and were conducted in accordance with institutional guidelines for animal welfare.

### Data availability.

Raw data for all statistics can be found in the Supporting Data Values file.

## Author contributions

XT conceived the original idea, designed the overall experimental strategy, and performed the majority of the experiments. XT also collected and interpreted the primary preclinical data and drafted the initial version of the manuscript. YS, XZ, YO, YJ, KP, and RLF contributed to the collection, processing, and analysis of patient-derived samples. They participated in data interpretation and provided critical feedback on the clinical relevance of the findings. PY performed a subset of the animal experiments, including experimental execution and data collection, and assisted in the interpretation of in vivo results. HD provided essential reagents, technical tools, and methodological support required for the completion of this study and offered expert advice during experimental optimization. JC conducted imaging acquisition and quantitative imaging analysis and contributed to the visualization and interpretation of imaging data. LZ, SZ, JH, KL, HY, YH, and ZW assisted with various experiments, including sample preparation, data acquisition, and technical support, and contributed to the reproducibility of the experimental results. JNS and LW provided essential reagents. ZL and RWM jointly conceptualized the study, supervised the overall research project, and provided strategic guidance throughout the study. RWM is the principal investigator of NCI10291. XT, XZ, YS, YO, and LZ contributed equally to this work and share first authorship. The order of co–first authors was assigned based on the relative extent of their contributions to the study, including study conception and design, experimental execution, data generation and analysis, and manuscript preparation.

## Conflict of interest

The authors have declared that no conflict of interest exists.

## Funding support

This work is the result of NIH funding, in whole or in part, and is subject to the NIH Public Access Policy. Through acceptance of this federal funding, the NIH has been given a right to make the work publicly available in PubMed Central.

- Mayo Foundation (R01 CA264600 to ZL).
- American Society for Radiation Oncology.
- Mayo Clinic Research Pipeline K2R Award, K12 (HD065987).
- NIH grant R01 CA 261932 (to RWM).
- NIH grant R01 CA 272602 (to RWM and ZL).
- Eagle fellowship award (to XT).
- Wallace and Evelyn Simmers Career Development Award for Cancer Research (to XT).
- Breast SPORE (P50 CA116201).
- Cancer Center grant (P30 CA015083).
- NCI10291 was sponsored by NCI-CTEP under the auspices of the Experimental Therapeutics Clinical Trials Network and was supported by UM1 CA186709.

## Supplementary Material

Supplemental data

Unedited blot and gel images

Supporting data values

## Version 1 07/07/2026

In-Press Preview

## Version 2 08/17/2026

Electronic publication
