# Supplementary material for: ATR senses stiff extracellular matrix to promote epithelial-to-mesenchymal transition and immune suppression
Source: J Clin Invest. 2026 Jul 7;136(16):e192285. doi: 10.1172/JCI192285 (PMC13476147; doi:10.1172/JCI192285)
Supplement: Supplementary file 1 [file jci-136-192285-s089.pdf]

Cytometry by time of flight (CyTOF)

Sample and Processing Mouse Samples

Isolated cells (3-4 million) derived from tumors or spleen are resuspended in 1 mL of Maxpar / Fluidigm Cell Staining Buffer (CSB) (catalog number: 201068). Each sample was incubated for 5 minutes with 0.5 m Cisplatin solution(catalog number 201064, Fluidigm/Cell-ID Cisplatin reagent) in PBS. Samples were then washed twice with CSB. An antibody cocktail of the entire phenotyping or function panel was prepared as a master mix and filtered to remove aggregates prior to adding 50 μL of cocktail to samples resuspended in 50 μ L of CSB. Samples were then incubated at room temperature for 45 minutes. After washing twice with CSB, samples were fixed with 2% PFA in PBS.

Preparation and Processing of Human Samples

Tumor-, spleen-, and PBMC-derived cells were processed using standard CyTOF staining protocols, including viability staining, antibody labeling, and fixation. Human PBMCs were isolated by Ficoll density centrifugation, cryopreserved, thawed, Fc-blocked, and stained with CyTOF antibody panels.

Samples were acquired on a Helios CyTOF system (Fluidigm). Data were normalized using CytoNorm (1,2), cleaned using FlowJo, and analyzed with Cytofkit and Rphenograph (3–5). Dimensionality reduction was performed using tSNE, and cluster abundance and marker expression were visualized by heatmaps and density plots.

Data Analysis

Cleanup of cell debris, removal of doublets and dead cells was performed using FloJo software version 10.5.3 (Ashland, OR). To minimize batch effects, all human samples were normalized against a reference standard using the CytoNorm algorithm (1, 2). Cleaned fcs files were analyzed by the R-based tool Cytofkit version 3.8 (3-5). Clustering and dimensionality reduction to 10,000 events per file was performed using the Rphenograph algorithm. Visualization of clusters was mapped onto a tSNE map. Relative marker intensities and cluster abundances per sample were visualized by a heatmap or density maps.

Migration and invasion assay

Migration assays were performed using 24-mm Transwell inserts with 8.0-μm polycarbonate membranes according to the manufacturer's instructions. Cells were serum-starved for 24 h, seeded into Transwell inserts, fixed with methanol, and stained with 5% crystal violet after the indicated incubation period.

For collagen invasion assays, cells were seeded onto collagen gels and cultured under serum-free conditions for 3 days, then fixed with 3% paraformaldehyde, permeabilized with 0.5% Triton X-100, stained with DAPI, and assessed for invasion depth.

For microchannel assays, cells were seeded into the right port of UVO microchannels (5250), while 3.5 mg/mL collagen was loaded into the left port. Invasion was quantified by the distance of cell migration into collagen.

Figure S1. ATR senses ECM stiffness through USP21

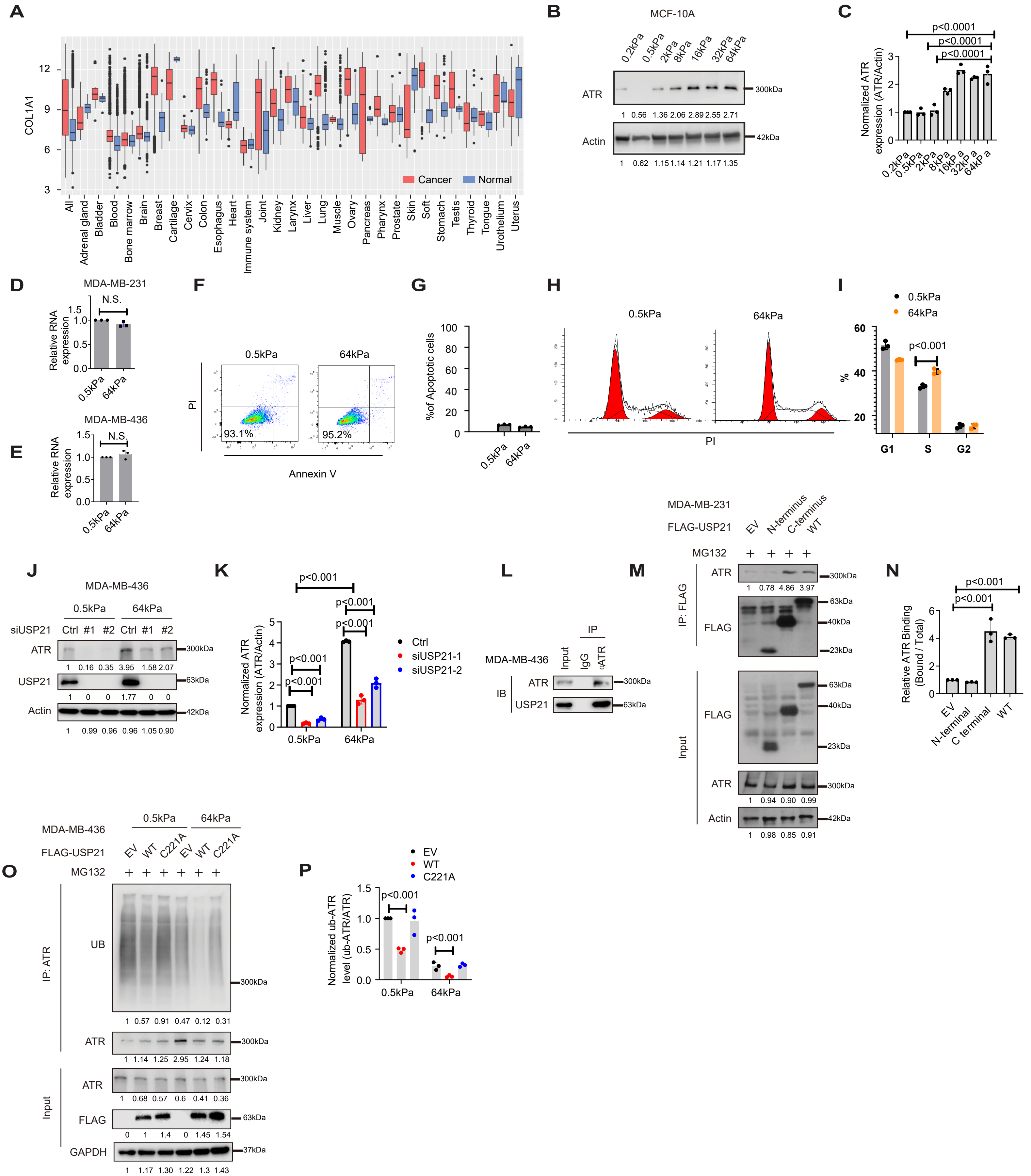

Figure S2. ATR promotes tumor invasion and metastasis

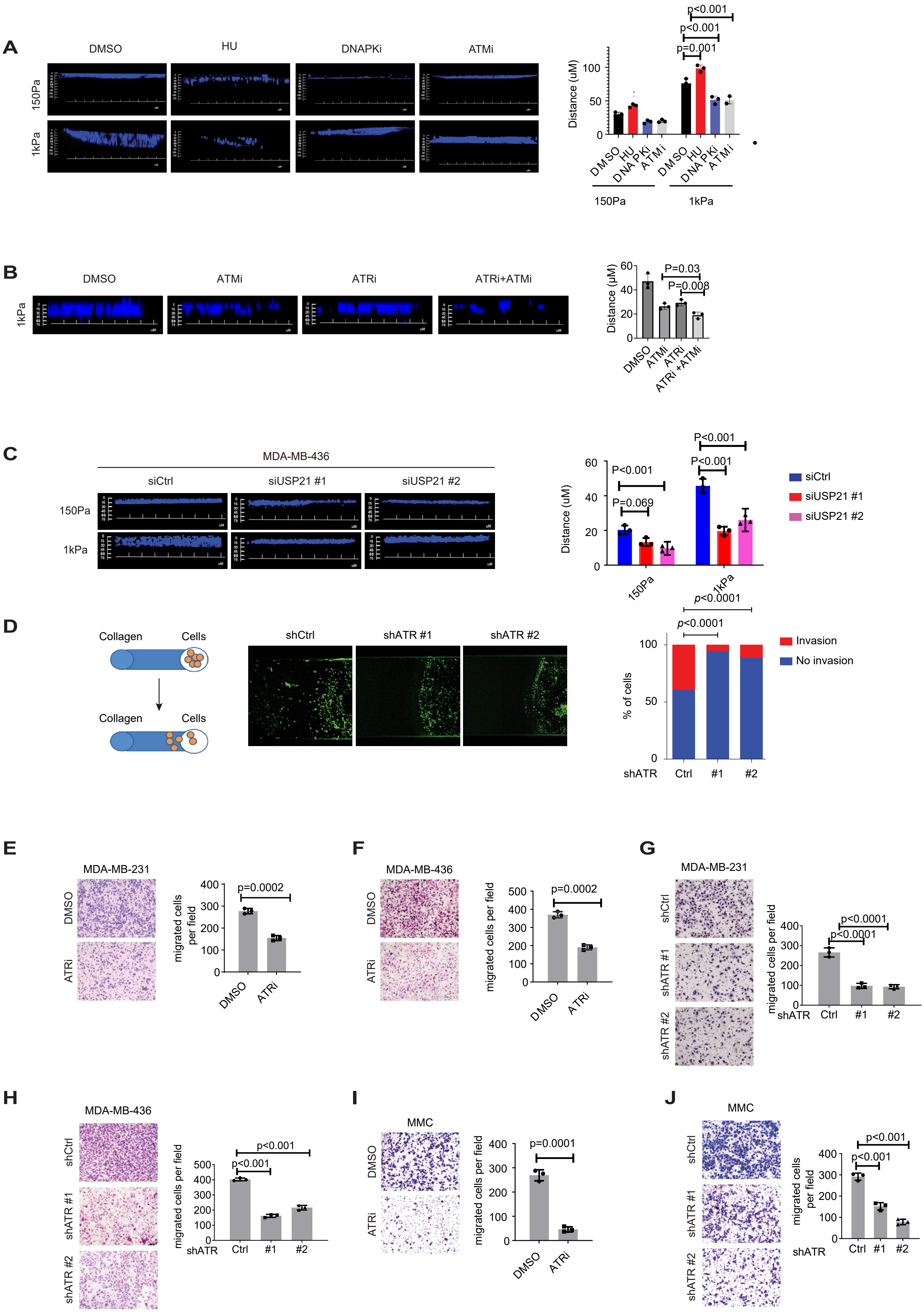

Figure S3. ATR Facilitates cytoskeleton remodeling induced EMT Process

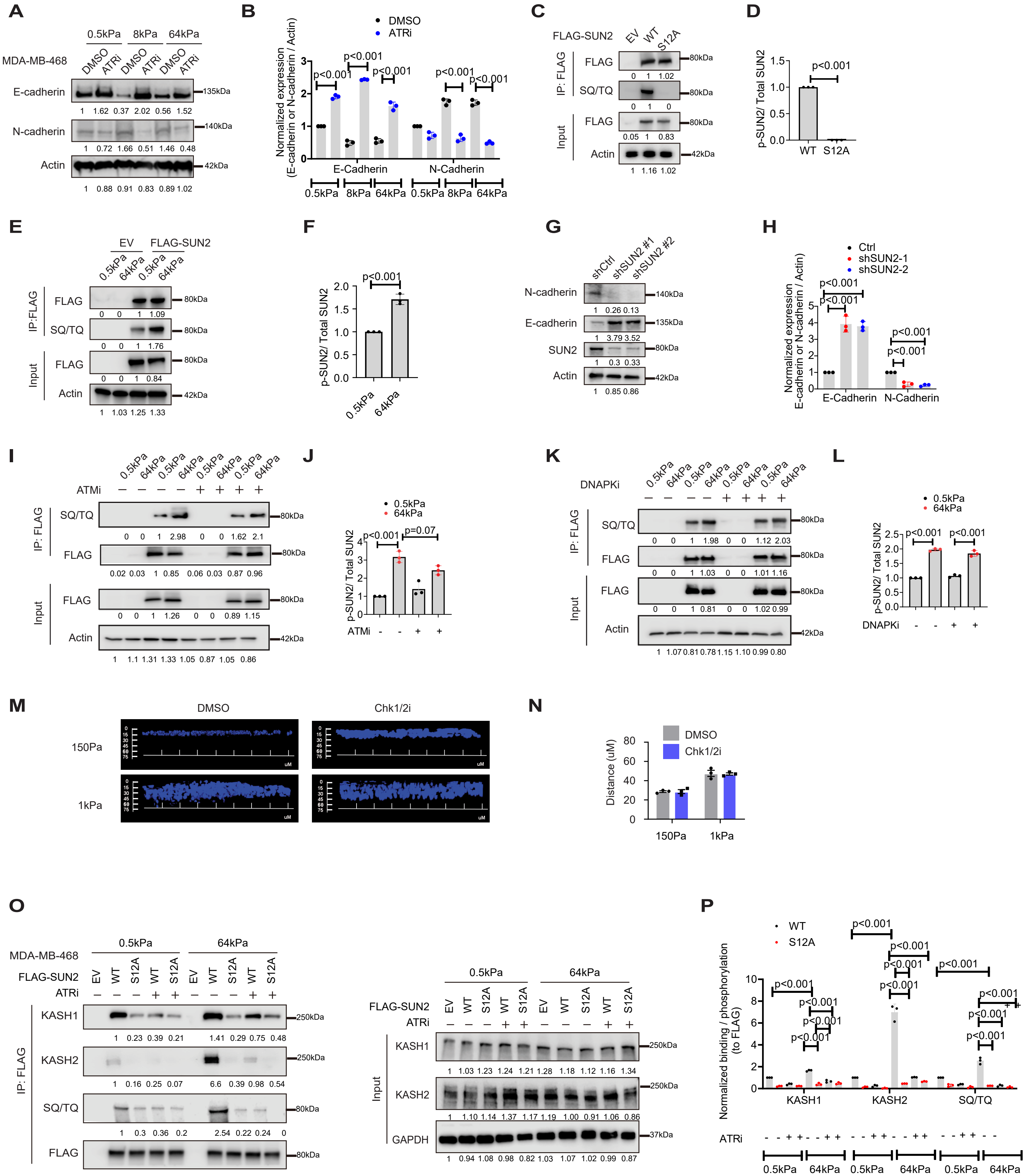

FigureS4. ATR inhibition suppresses ECM stiffness induced EMT through SUN2

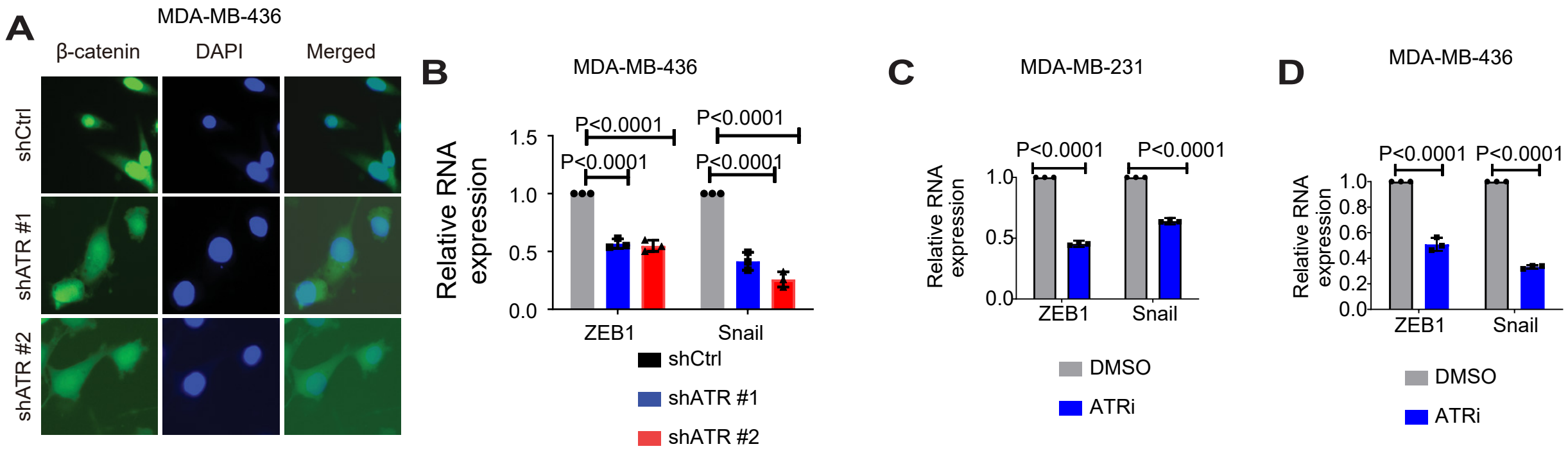

Figure S5. ATR inhibition activate anti-tumor immunity

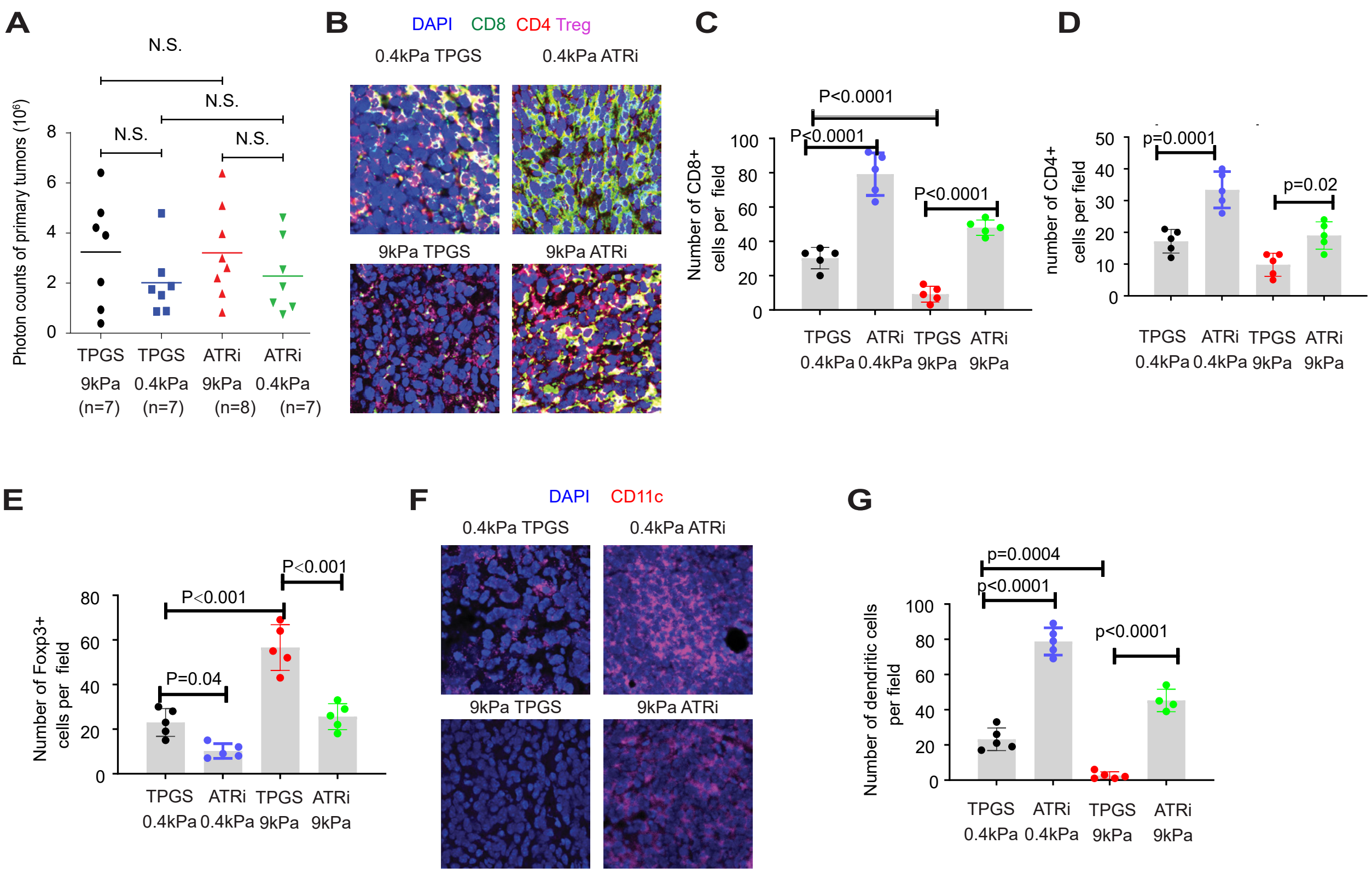

Figure S6. ATR inhibition sensitizes tumors to immunotherapy

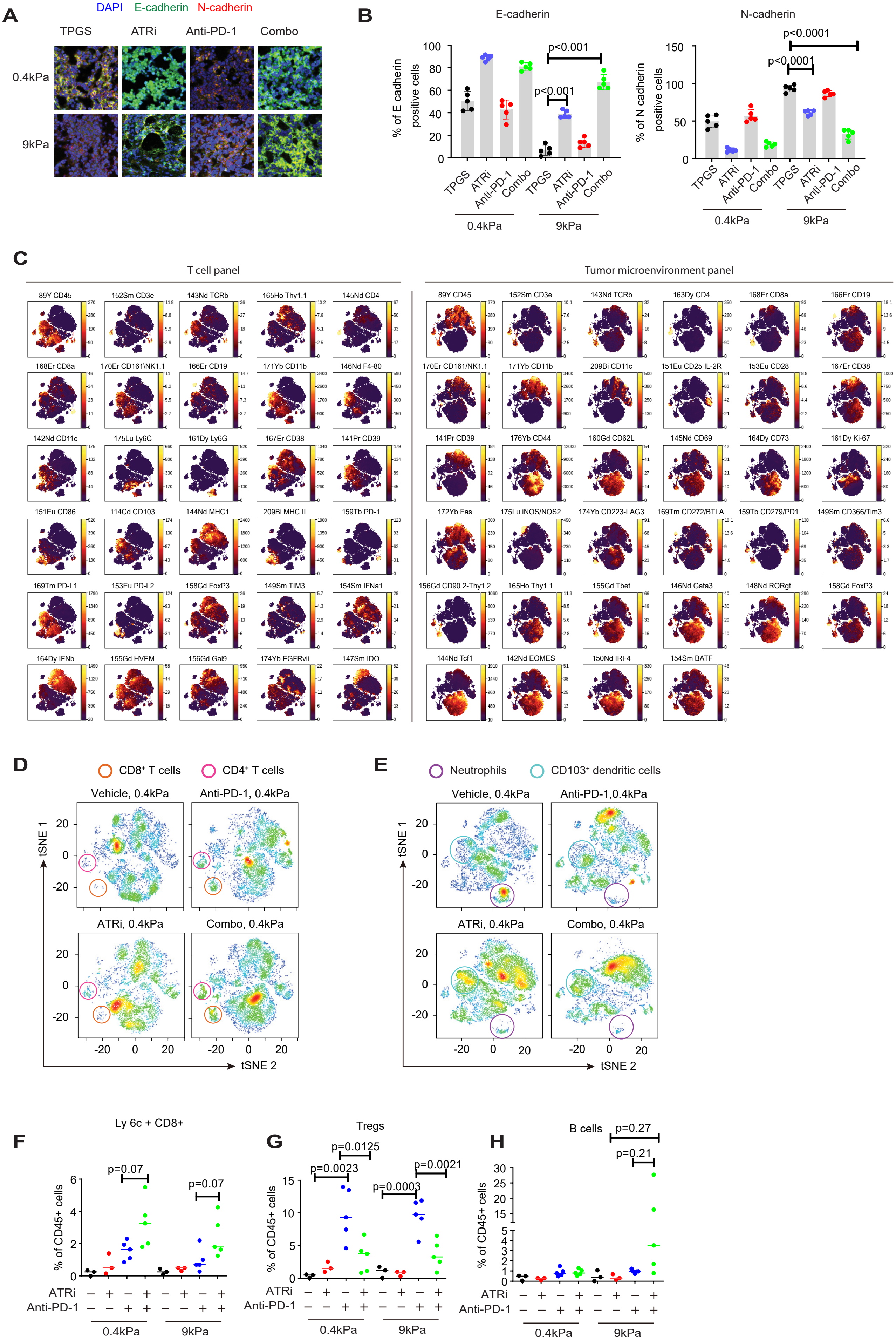

Figure S1. ATR senses ECM stiffness through USP21

(A) Expression of COL1A1 is found to be significantly elevated in various types of cancer, including breast cancer, as per the data obtained from the GENT2 database. **(B)** The expression of ATR in MCF-10A cells cultured on surfaces with varying stiffness was analyzed by western blotting. Actin served as a loading control. **(C)** The statistical analysis of the Western blot results shown in panel B was performed using a one-way ANOVA (n = 3). **(D-E)** MDA-MB-231 and MDA-MB-436 cells were cultured on different stiffness, and then ATR mRNA levels were determined by qRT-PCR (n=3). Statistical significance was determined using t-test. **(F-G)** MDA-MB-231 cells were cultured on surfaces with different stiffness, and apoptosis was subsequently assessed by flow cytometry (n=3). Statistical significance was determined using t-test. **(H-I)** MDA-MB-231 cells were cultured on surfaces with different stiffness, and cell cycle distribution was assessed by flow cytometry. (n=3). Statistical significance was determined using a two-way ANOVA (n = 3) **(J)** MDA-MB-436 cells were transfected with either control or USP21-specific siRNA and cultured on surfaces with varying stiffness for 48 hours. The expression of USP21 and ATR was then determined. **(K)** The statistical analysis of the Western blot results shown in panel J was performed using a two-way ANOVA (n = 3). (**L**) The interaction between USP21 and ATR in MDA-MB-436 cells was assessed by immunoprecipitation using ATR antibody. **(M)** MDA-MB-231 cells were infected with lentivirus encoding either vector, FLAG-tagged USP21 WT, FLAG-tagged USP21 N-terminus, or FLAG-tagged USP21 C-terminus, and the interaction of ATR with USP21 was determined by immunoprecipitation using FLAG antibody. **(N)** The statistical analysis of the Western blot results shown in panel M was performed using a one-way ANOVA (n = 3). **(O)** MDA-MB-436 cells were infected with lentivirus encoding either vector, wild type USP21, or C221A USP21 mutant, and seeded on surfaces with different stiffness. After 48 hours, the cells were harvested and performed IP using ATR antibody to assess ubiquitination. **(P)** The statistical analysis of the Western blot results shown in panel O was performed using a one-way ANOVA (n = 3).Statistical data represents biological replicates, and results are expressed as mean ± SD. Western blot and co-immunoprecipitation (co-IP) experiments were

independently repeated at least three times with similar results.

Figure S2. ATR promotes tumor invasion and metastasis

(A) MDA-MB-231 cells were seeded on collagen gel surfaces with different stiffness and treated with HU (1 mM), a DNA-PK inhibitor (NU7441, 1 μM), or an ATM inhibitor (AZD-0156, 100 nM). After 3 days under serum-free conditions, invasion depth was measured by DAPI staining (n = 3). The statistical analysis was performed using a two-way ANOVA. **(B)** MDA-MB-231 cells were seeded on collagen gels of varying stiffness and treated with the ATR inhibitor VX970 (80 nM), the ATM inhibitor AZD-0156 (100 nM), or a combination of both. After 3 days under serum-free conditions, invasion depth was assessed by DAPI staining (n = 3). Statistical comparisons between the combination treatment and each single-agent treatment were performed using Student's *t*-test, as each comparison involved two groups. **(C)** Either control or USP21-depleted MDA-MB-436 cells were seeded on collagen gel surfaces with different stiffness, and the invasion depth was measured by DAPI staining after 3 days under serum-free conditions(n=3). **(D)** Control or ATR knockdown cells were seeded in a microchannel and cell invasion through collagen was imaged and quantified by immunofluorescence. The statistical analysis was performed using Chi-square test **(E)** MDA-MB-231 cells were serum-starved for 24 hours and then treated with either DMSO (Control) or VX970 (80 nM) for 2 hours, followed by transwell migration assay. Cells were fixed with methanol and stained after 5 hours(n=3). **(F)** MDA-MB-436 cells were serum-starved for 24 hours and then treated with either DMSO (Control) or VX970 (80 nM) for 2 hours, followed by transwell migration assay. Cells were fixed with methanol and stained after 5 hours(n=3). Statistical significance was determined using t-test. **(G)** MDA-MB-231 cells were infected with lentivirus encoding either control or ATR shRNA, and the transwell migration assay was performed(n=3). The statistical analysis was performed using a one-way ANOVA. **(H)** MDA-MB-436 cells were infected with lentivirus encoding either control or ATR shRNA, and the transwell migration assay was performed(n=3). Statistical significance was determined using t-test. **(I)** ATR inhibition reduced the invasion of MDA-MB-231 cells independent of

replication stress. MDA-MB-231 cells were serum-starved for 24 hours. Then, either DMSO (Control) +MMC or VX970 (80 nM) +MMC were applied to cells for 2 hours before seeding them in transwell plates. Cells were fixed and stained after 5 hours (n=3). **(J)** MDA-MB-231 cells were infected with lentivirus encoding either control or ATR shRNA and then treated with MMC for 2 hours before seeding them in transwell inserts. Cells were fixed and stained after 5 hours (n=3). All statistical data represent biological replicates, and results are expressed as mean ± SD.

Figure S3. ATR Facilitates cytoskeleton remodeling induced EMT Process

(A) MDA-MB-468 cells were treated with either DMSO or VX970 (80 nM). After 24 hours, the cells were seeded on surfaces of varying stiffness. E-cadherin and N-cadherin levels were then assessed using Western blot. **(B)** The statistical analysis of the Western blot results shown in panel A was performed using a two-way ANOVA (n = 3). **(C)** MDA-MB-436 cells were infected with lentivirus encoding either empty vector, Flag-tagged SUN2 wild type, or Flag-tagged S12A SUN2 mutant. The cells were harvested and immunoprecipitated using FLAG antibody. The phosphorylation of SUN2 was detected using Phospho-(Ser/Thr) ATM/ATR Substrate Antibody. **(D)** The statistical analysis of the Western blot results shown in panel C is presented (n = 3) using t test. **(E)** MDA-MB-436 cells were infected with lentivirus encoding either empty vector or Flag-tagged SUN2 wild type. After 24 hours, the cells were seeded on surfaces with different stiffness. The cells were immunoprecipitated using FLAG antibody and the phosphorylation of SUN2 was detected using Phospho-(Ser/Thr) ATM/ATR Substrate Antibody. **(F)** The statistical analysis of the Western blot results shown in panel E is presented (n = 3) using t test. **(G)** MDA-MB-436 cells were infected with lentivirus encoding either control or shSUN2 and assessed for E-cadherin and N-cadherin expression. **(H)** The statistical analysis of the Western blot results shown in panel G was performed using a two-way ANOVA (n = 3). **(I)** Control and SUN2 overexpressing cells were seeded on different stiff surfaces and treated with DMSO or ATMi (AZD-0156, 100 nM) for 18 hours. Immunoprecipitation was then performed using FLAG antibody, the phosphorylation of SUN2 was then determined using

Phospho-(Ser/Thr) ATM/ATR Substrate. **(J)** The statistical analysis of the Western blot results shown in panel I was performed using a two-way ANOVA (n = 3). **(K)** Control and SUN2 overexpressing cells were seeded on different stiff surfaces and treated with DMSO or DNAPKi (NU7441, 1 μM) for 18 hours. Immunoprecipitation was then performed using FLAG antibody, the phosphorylation of SUN2 was then determined using Phospho-(Ser/Thr) ATM/ATR Substrate. **(L)** The statistical analysis of the Western blot results shown in panel K was performed using a two-way ANOVA (n = 3). **(M-N)** Chk inhibition did not affect the invasion of MDA-MB-231. MDA-MB-231 treated with either DMSO or AZD7762 (100nM) were seeded on collagen gel surfaces with different stiffness and incubated for 3 days in serum-free condition. The invasion depth was measured by DAPI staining.

(O) MDA-MB-436 cells infected with lentivirus encoding either empty vector, wild type SUN2, or S12A SUN2 mutant were seeded on surfaces with different stiffness and treated with either DMSO or VX970. The cells were immunoprecipitated using FLAG antibody. **(P)** The statistical analysis of the Western blot results shown in panel O was performed using a two-way ANOVA (n = 3). Western blot and co-immunoprecipitation (co-IP) experiments were independently repeated at least three times with similar results.

Figure S4. ATR inhibition suppresses ECM stiffness-induced EMT through SUN2

(A) Immunofluorescence images show the localization of β-catenin in MDA-MB-436 cells infected with lentivirus encoding control or shATR. **(B)** MDA-MB-436 cells infected with lentivirus encoding either control or shATR were harvested and analyzed for the mRNA level of ZEB1 and snail by qRT-PCR (n=3). Statistical significance was determined using one-way ANOVA. **(C)** ATR inhibition reduced the expression level of ZEB1 and Snail1. MDA-MB-231 cells treated with either DMSO or VX970 (80nM) were harvested and analyzed for the mRNA level of ZEB1 and snail by qRT-PCR(n=3). Statistical significance was determined using t-test. **(D)** MDA-MB-436 cells treated with either DMSO or VX970 (80nM) were assessed for the mRNA level of ZEB1 and snail by qRT-PCR (n=3). Statistical significance was determined using t-test. All statistical

data represent biological replicates, and results are expressed as mean ± SD.

Figure S5. ATR inhibition activates anti-tumor immunity

(**A**) The photon counts of primary tumors were quantified using Xenogen IVIS 200 at the end of the experiment. Statistical significance was determined using one-way ANOVA. **(B-E)** Representative IHC images and statistics for CD4+, CD8+ and Tregs detection performed on tumor tissues harvested from primary tumors. Quantification from five randomly selected fields was used for statistical analysis (n=5). Statistical significance was determined using one-way ANOVA. **(F-G)** The images and statistics of the IHC data for CD11c+ cells from the 4T-1 model. Quantification from five randomly selected fields was used for statistical analysis (n=5). Statistical significance was determined using one-way ANOVA. All statistical data represent biological replicates.

Figure S6. ATR inhibition sensitizes tumors to immunotherapy

(A) The IHC data of E cadherin and N cadherin from primary tumors are schematically represented. **(B)** The statistics of the IHC data from **(A).** Quantification from five randomly selected fields was used for statistical analysis (n=5). Statistical significance was determined using two-way ANOVA. **(C)**The staining intensity for all markers for T cell panel and tumor microenvironment panel**. (D-E)** The tSNE plots for T cells and tumor microenvironment panel from low stiffness tumors in Figure 6. All statistical data represent biological replicates. (F-H) The proportions of $Ly6C^{+}$ $CD8^{+}$ cells, B cells, and Tregs in tumors from different groups were quantified.
