# Supplementary material for: ATR senses stiff extracellular matrix to promote epithelial-to-mesenchymal transition and immune suppression
Source: J Clin Invest. 2026 Jul 7;136(16):e192285. doi: 10.1172/JCI192285 (PMC13476147; doi:10.1172/JCI192285)

1E

ATR

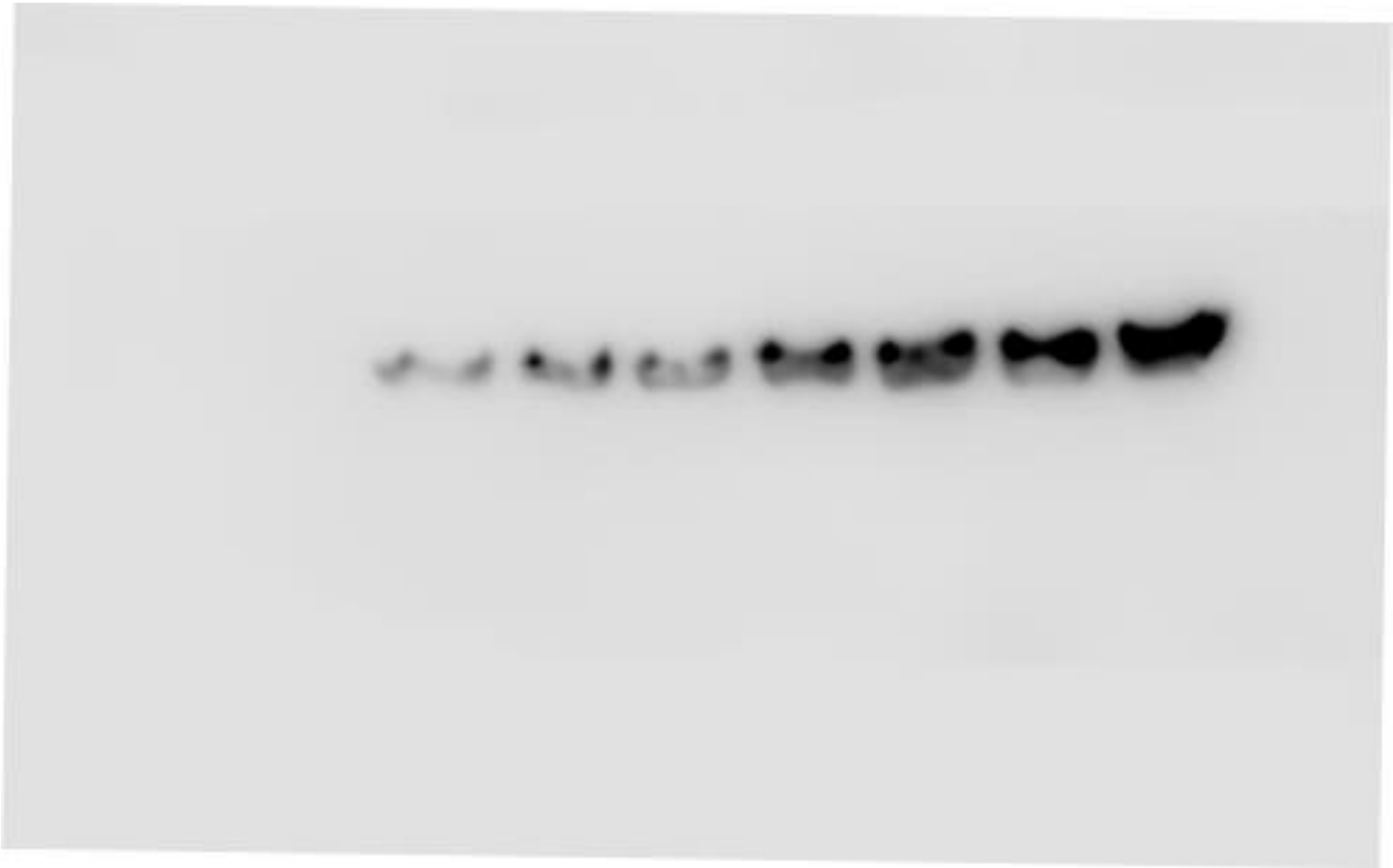

Actin

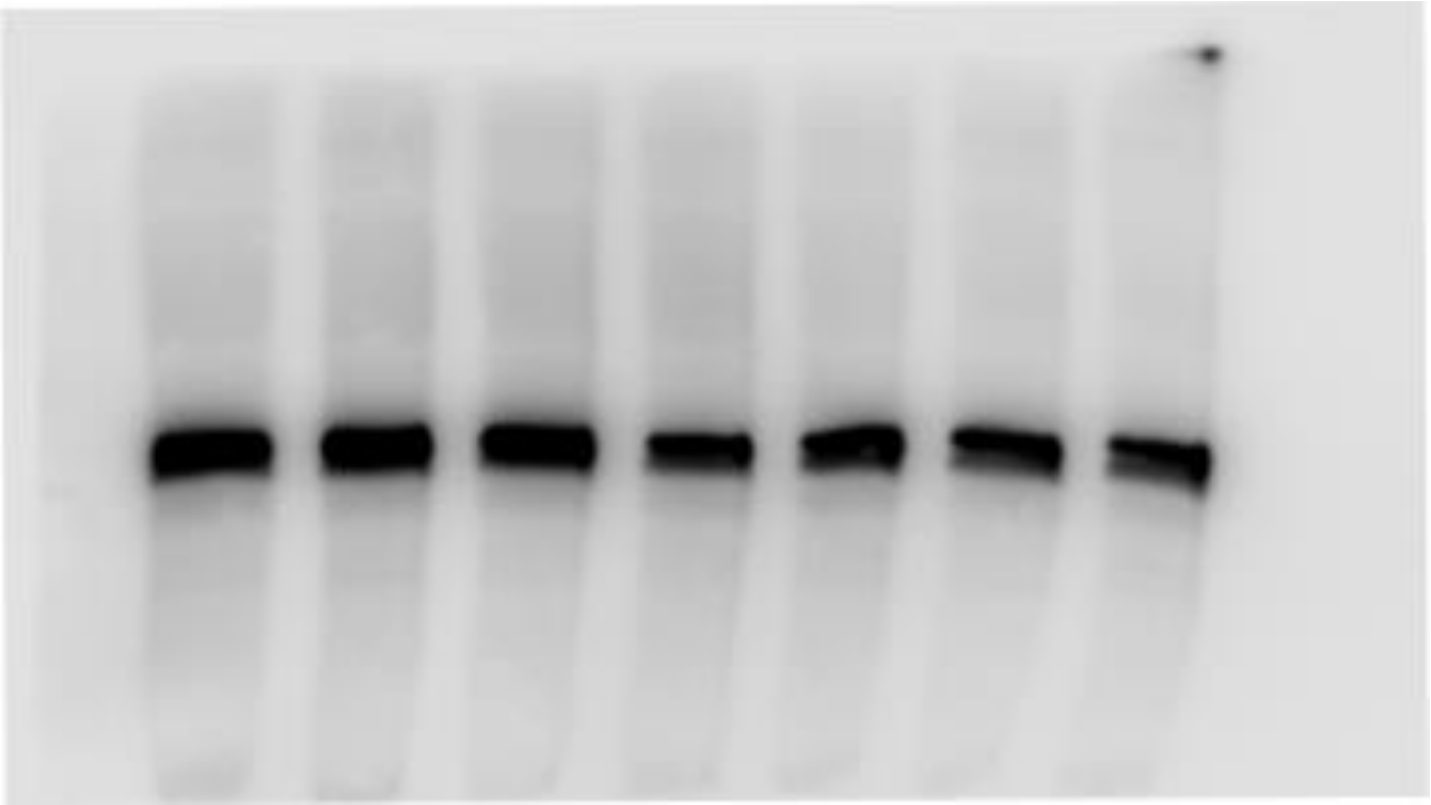

Figure 1h

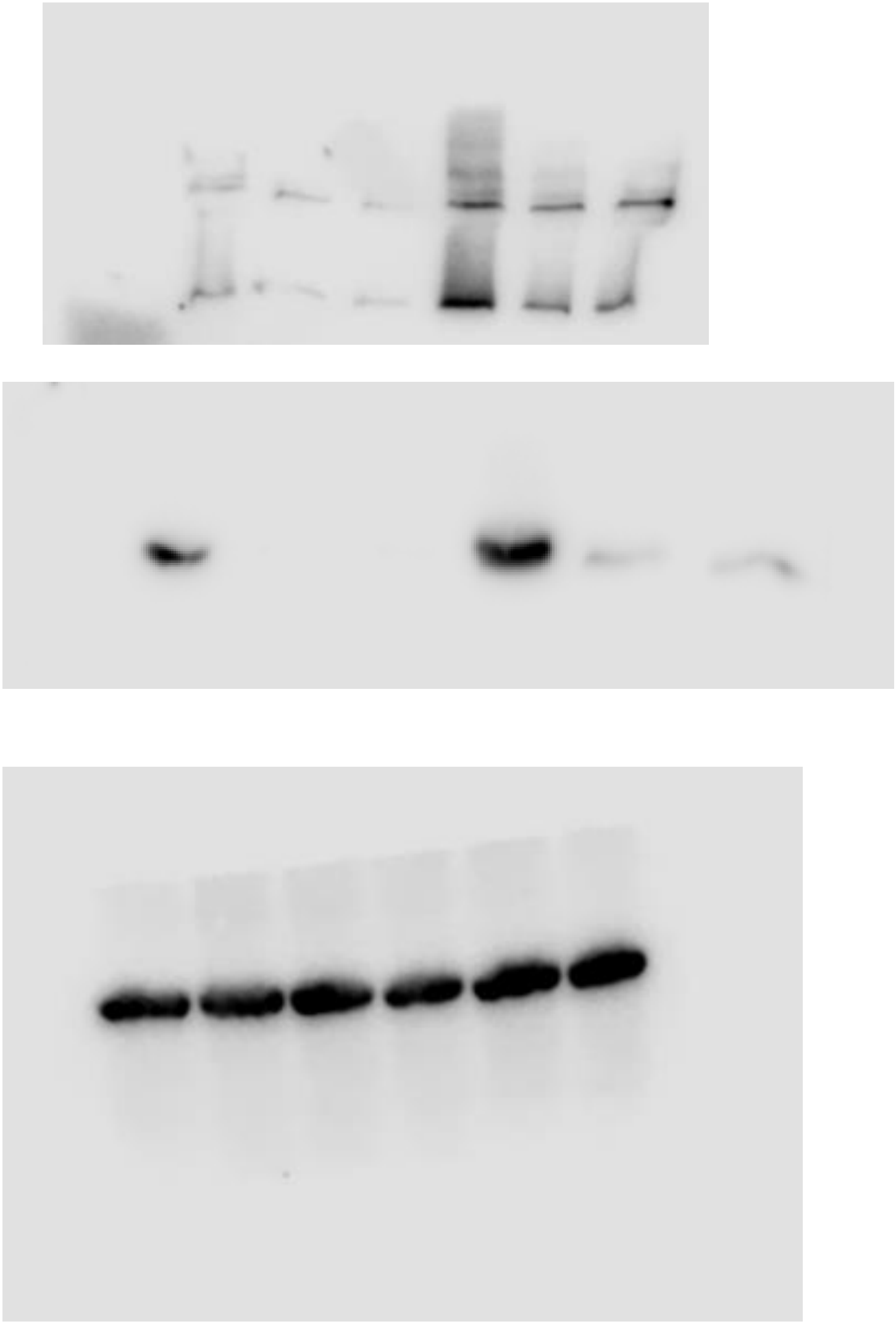

Figure 2D

USP21

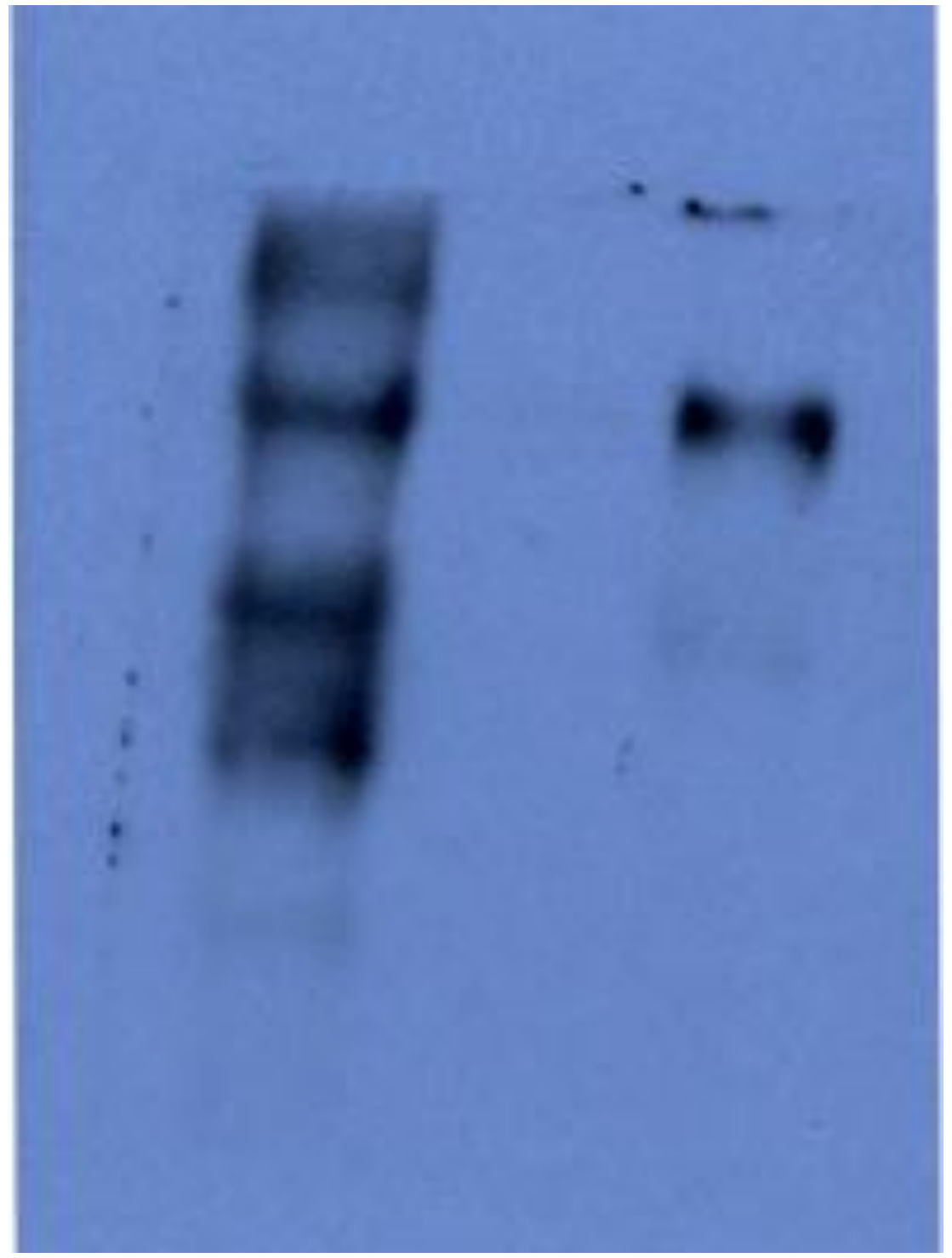

ATR

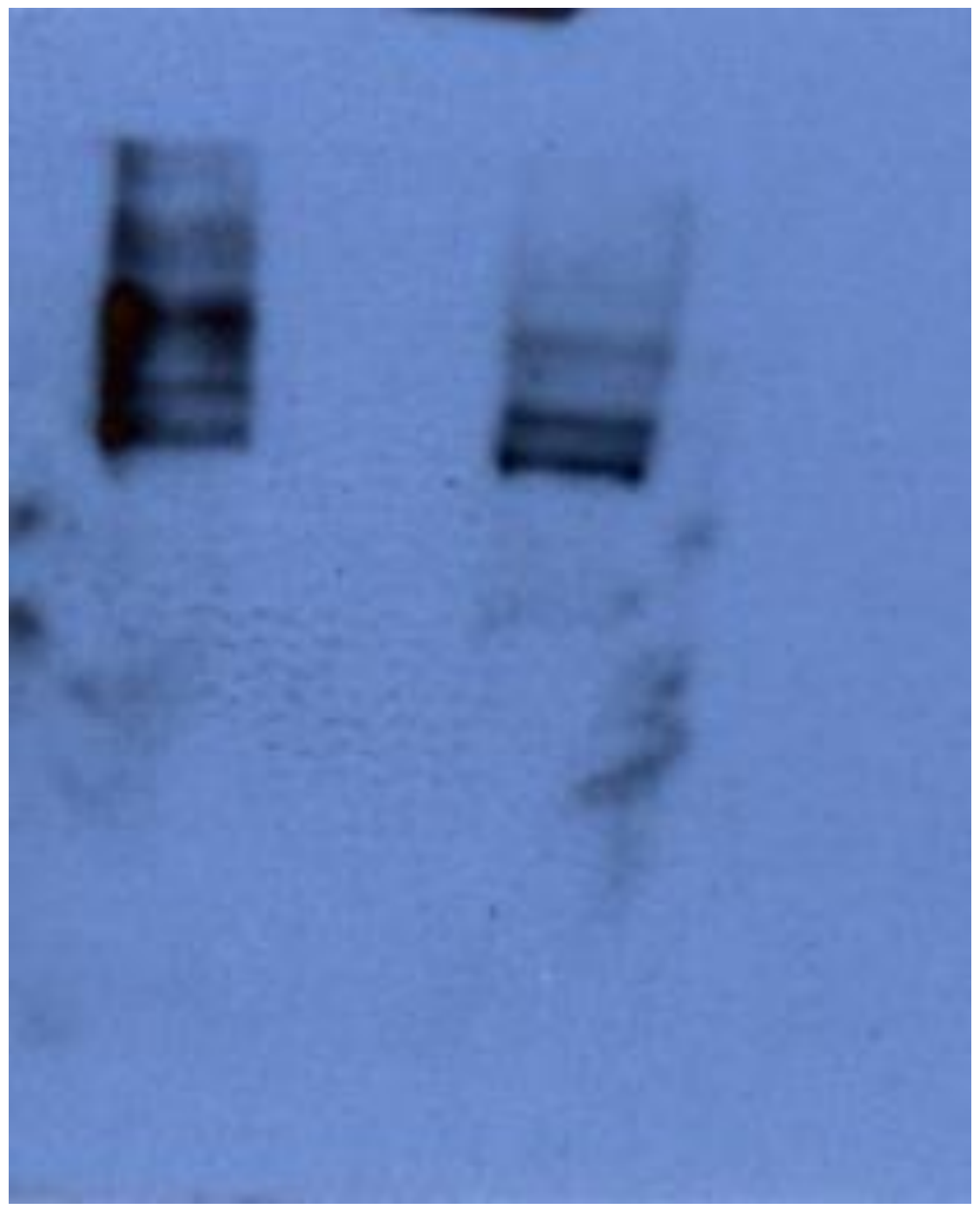

2E

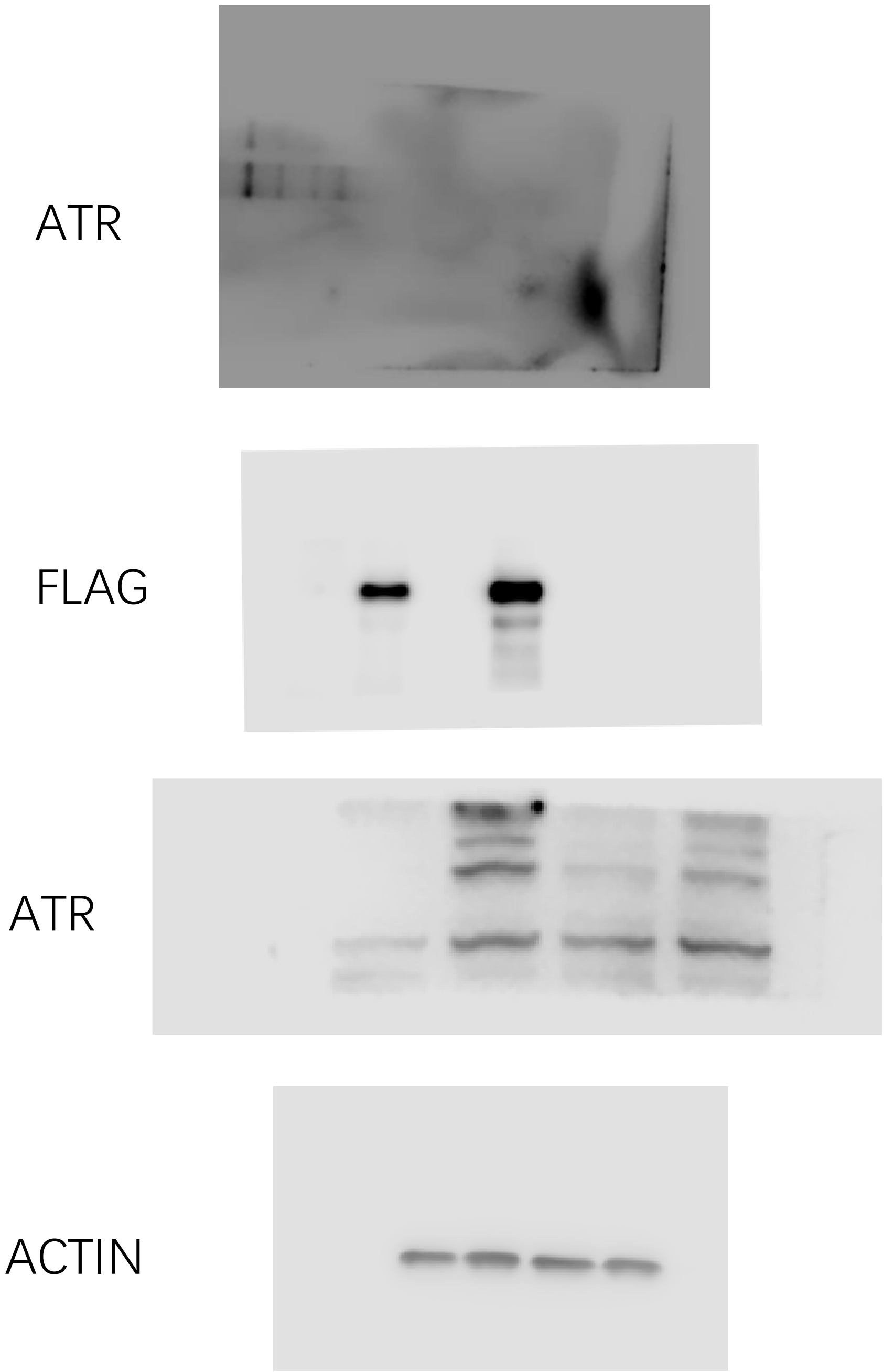

2G

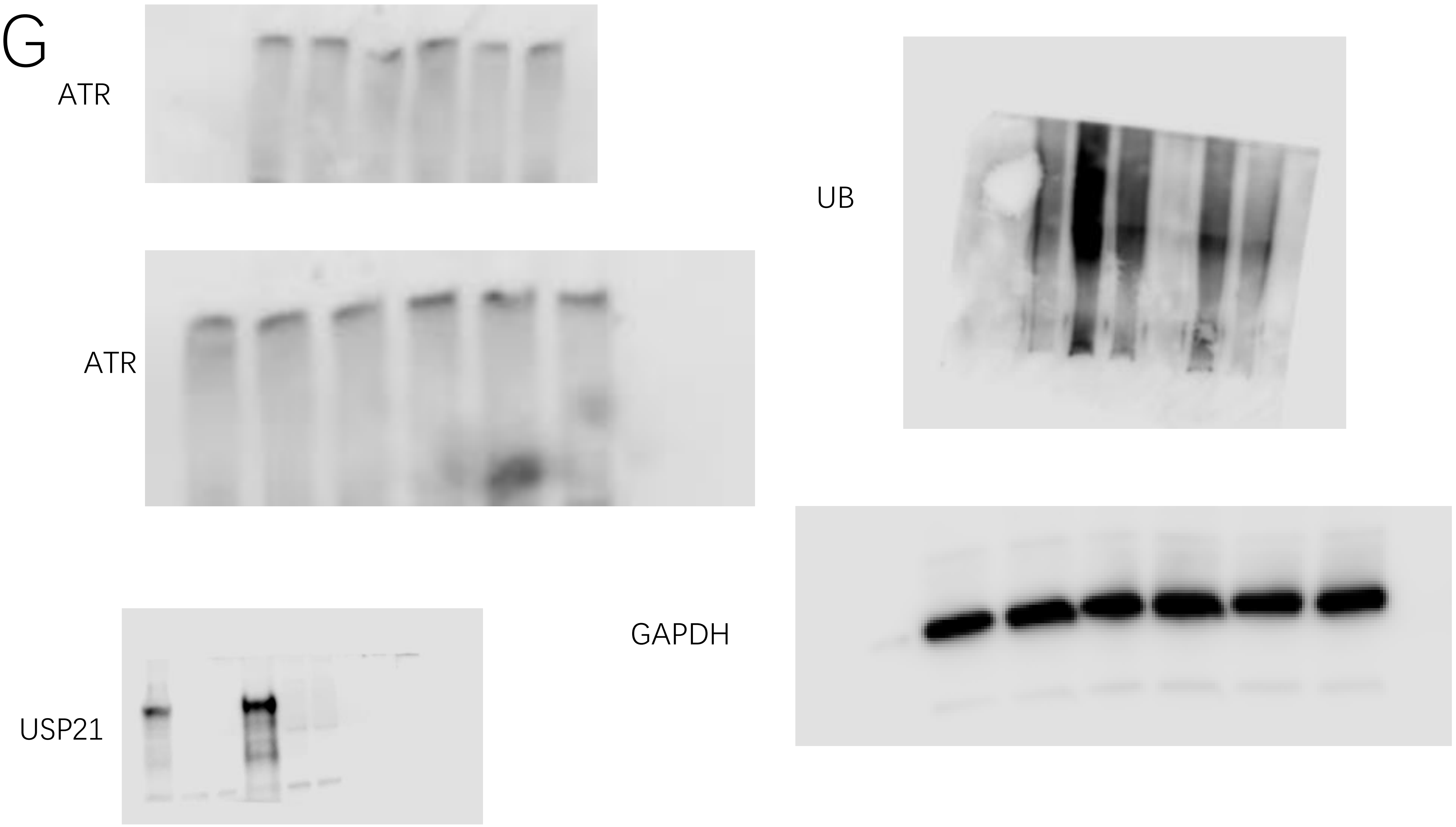

2H

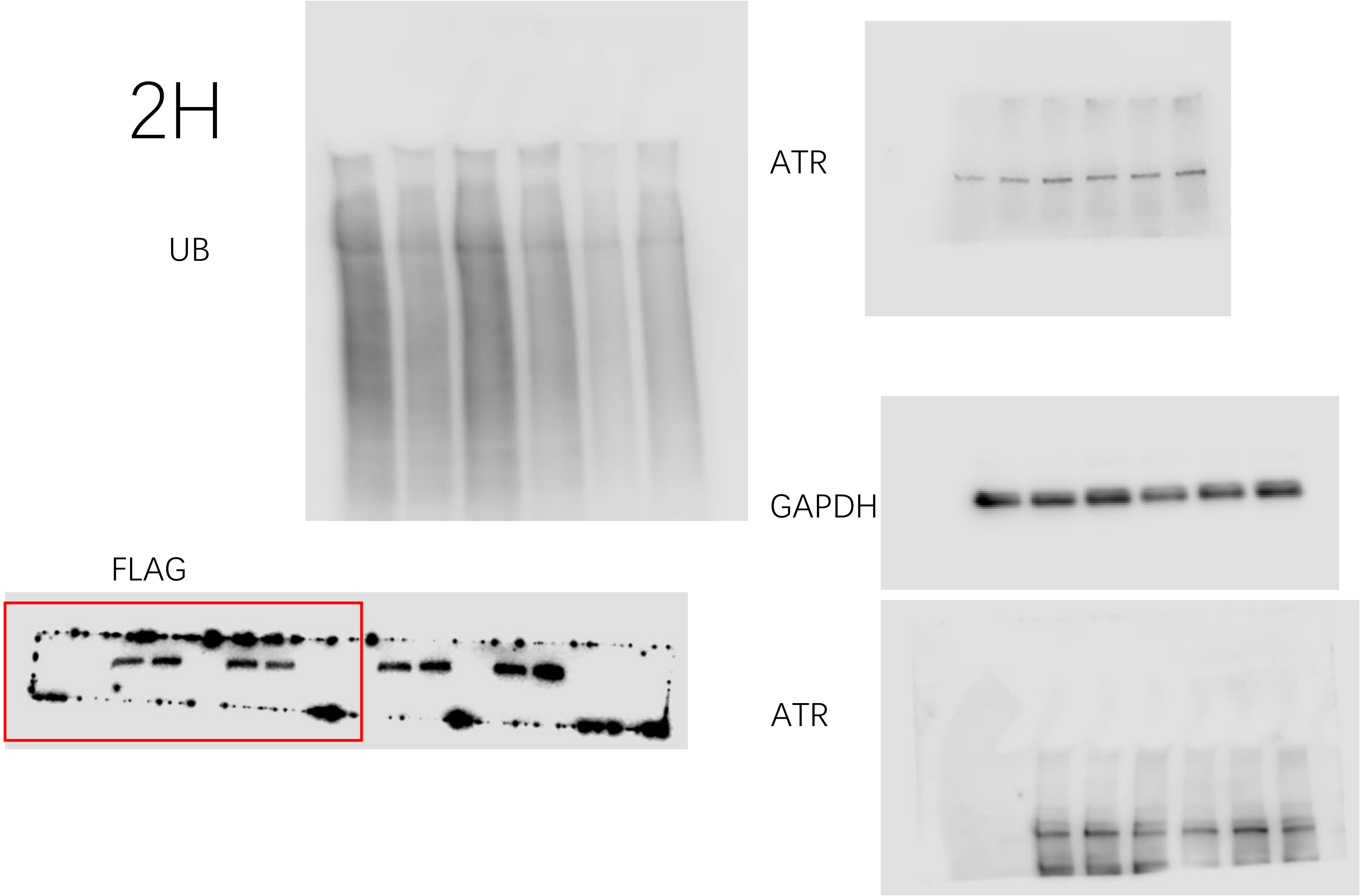

4A

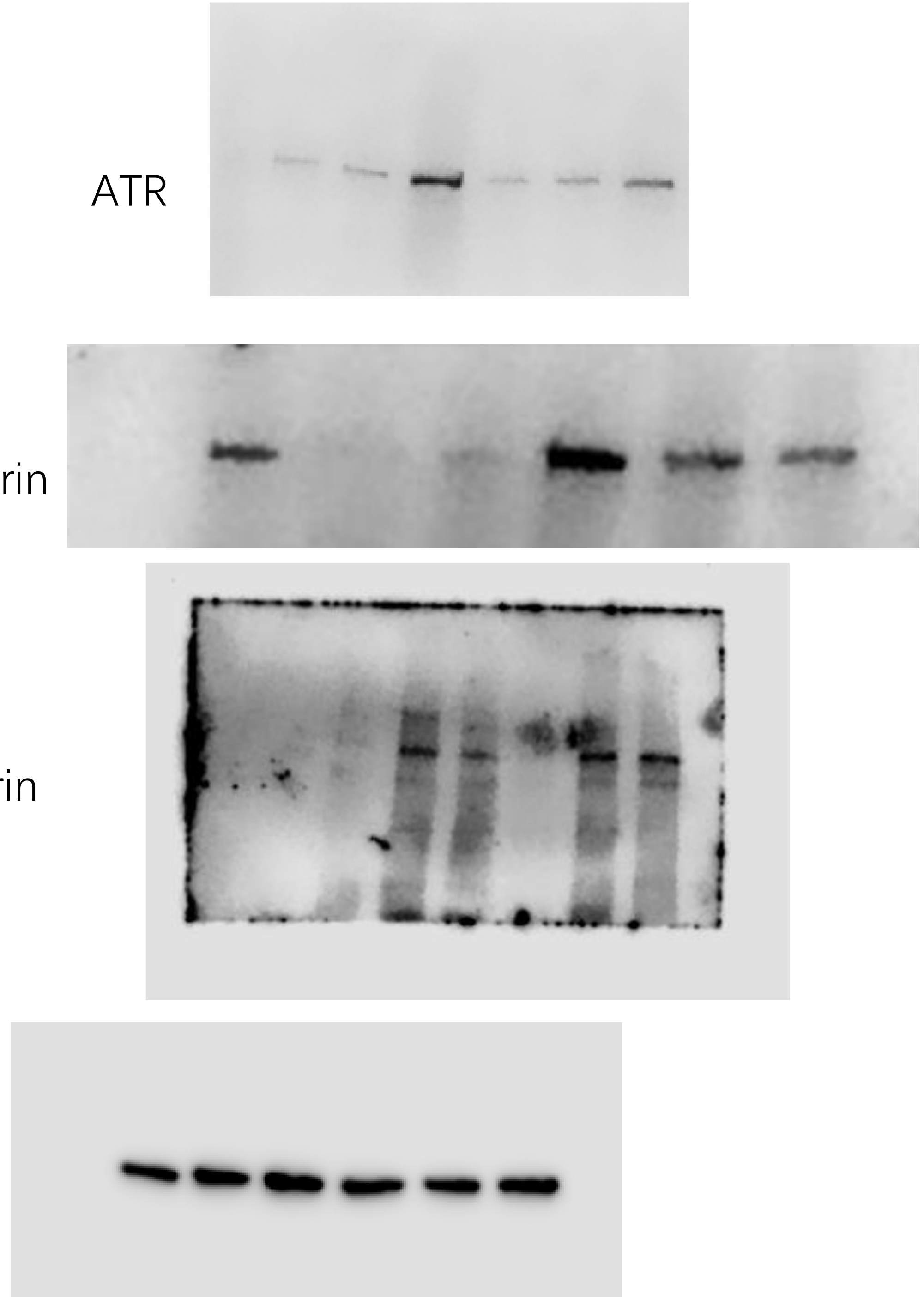

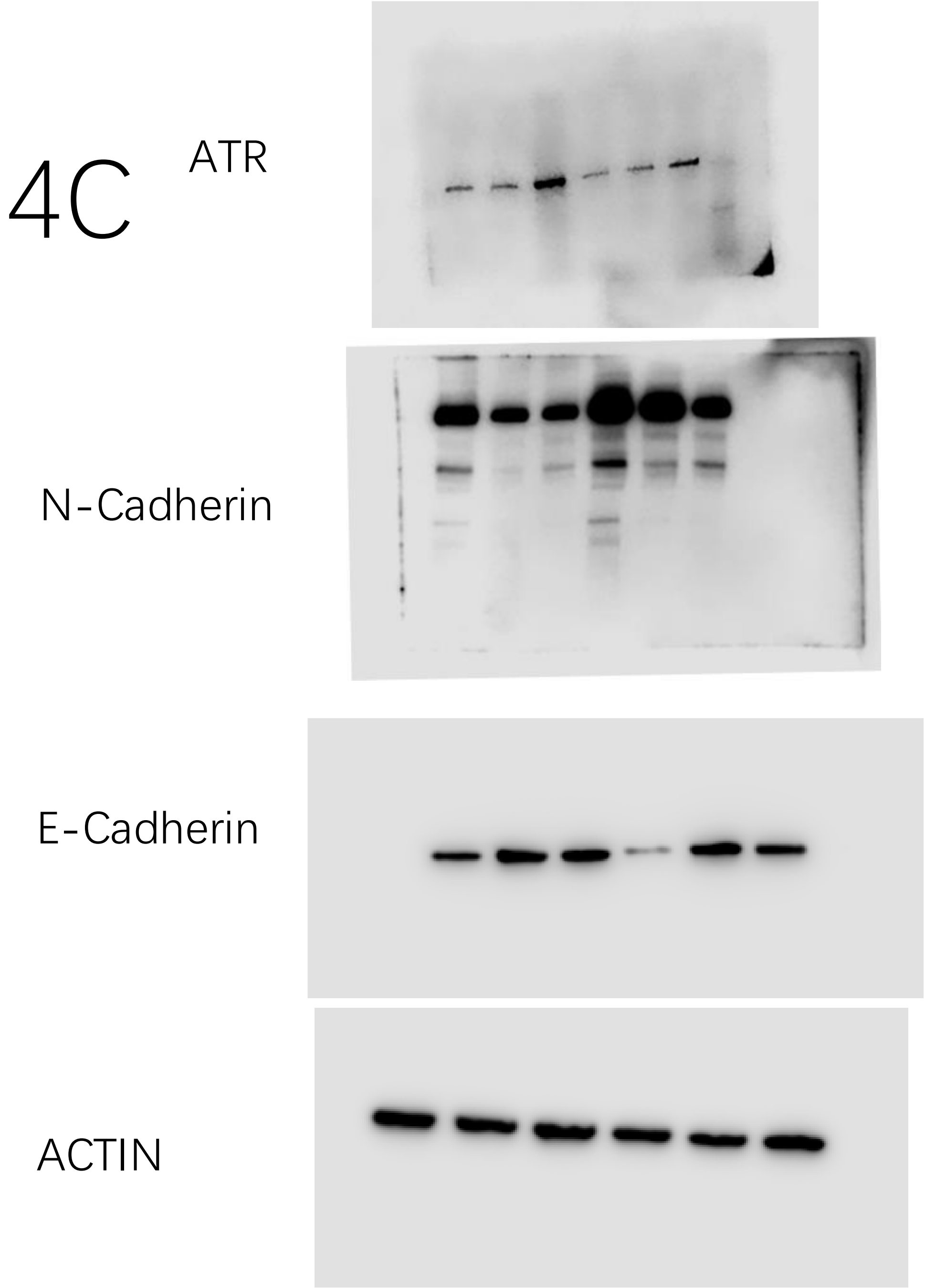
4C
ATR
N-Cadherin
E-Cadherin
ACTIN

Figure 4E

E Cadherin

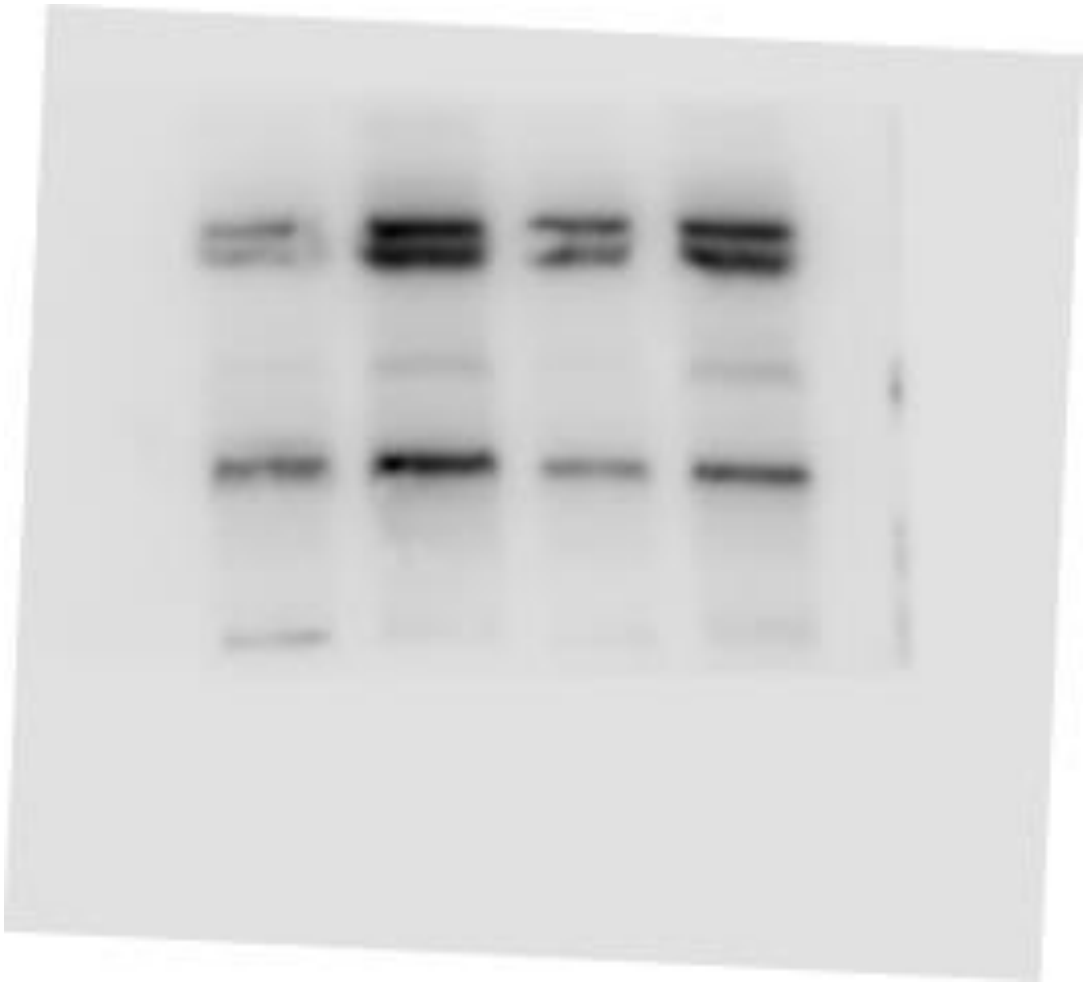

N Cadherin

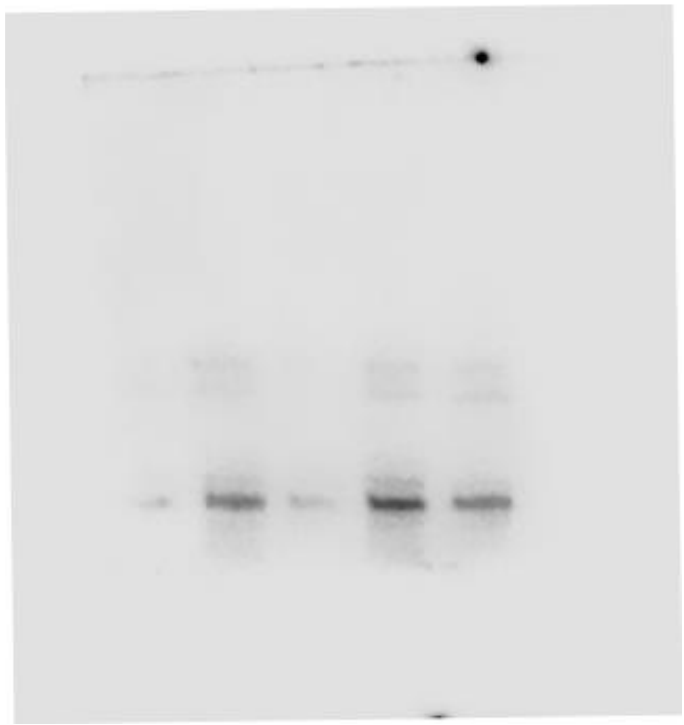

ACTIN

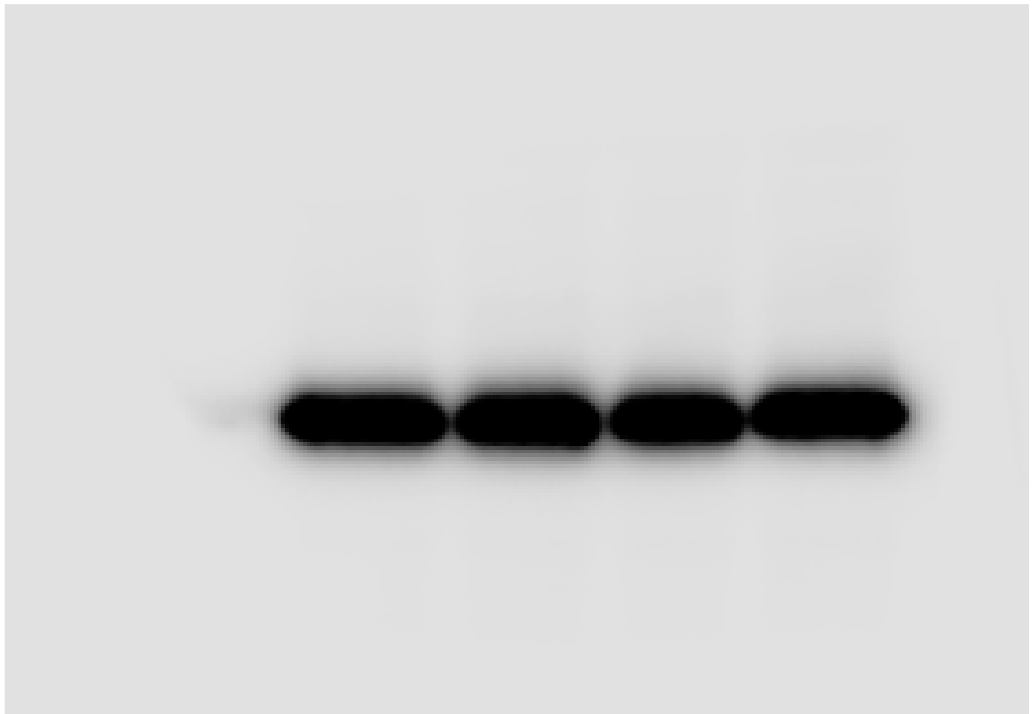

Figure 4G

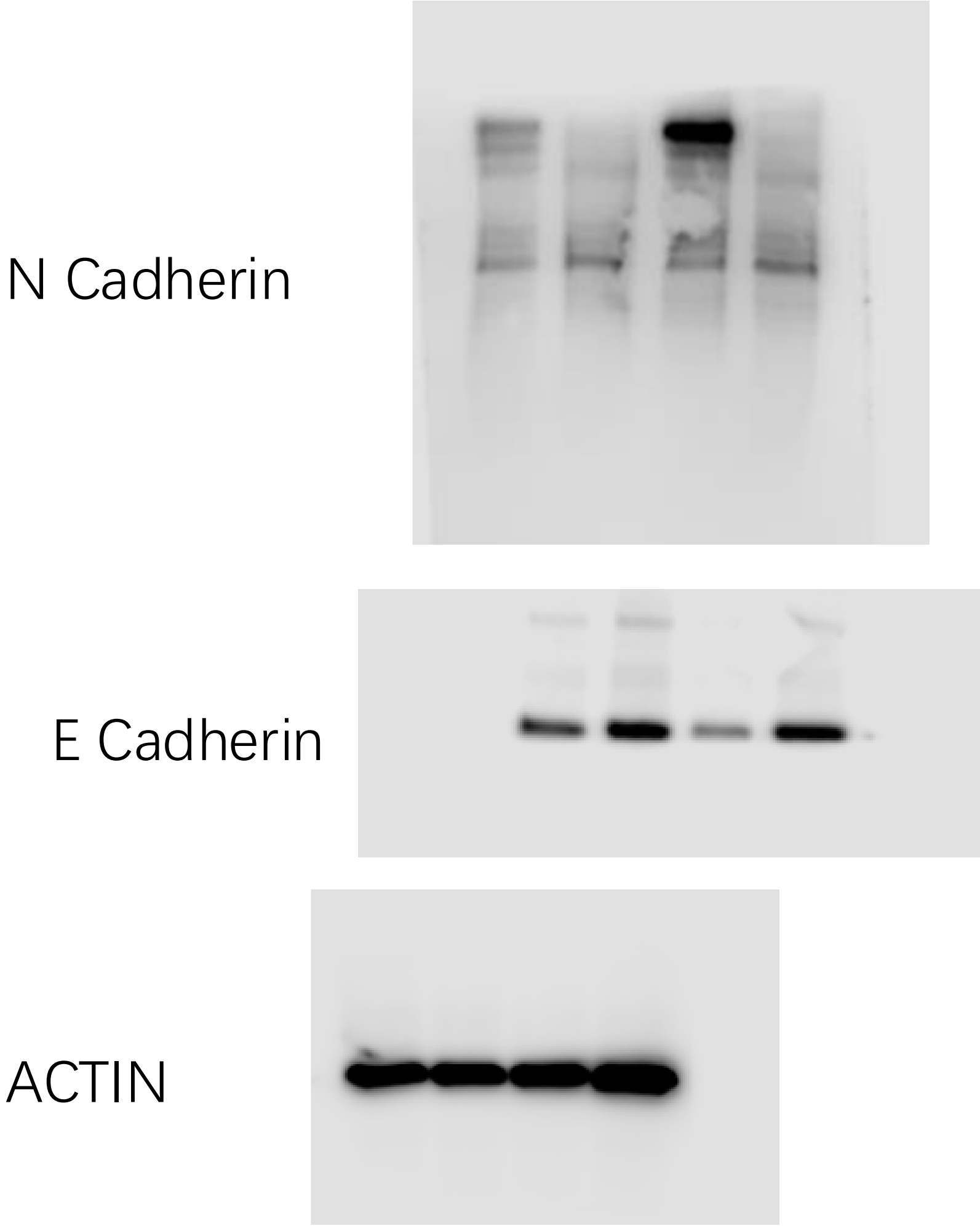

Figure 5A

FLAG

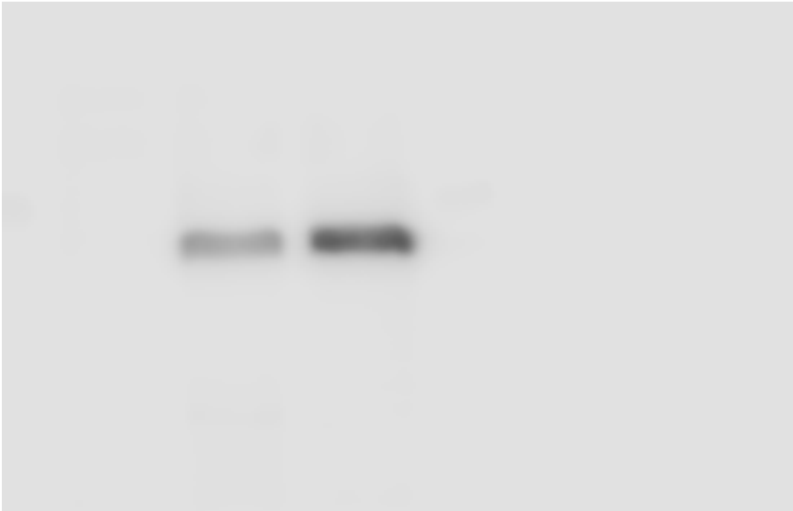

FLAG

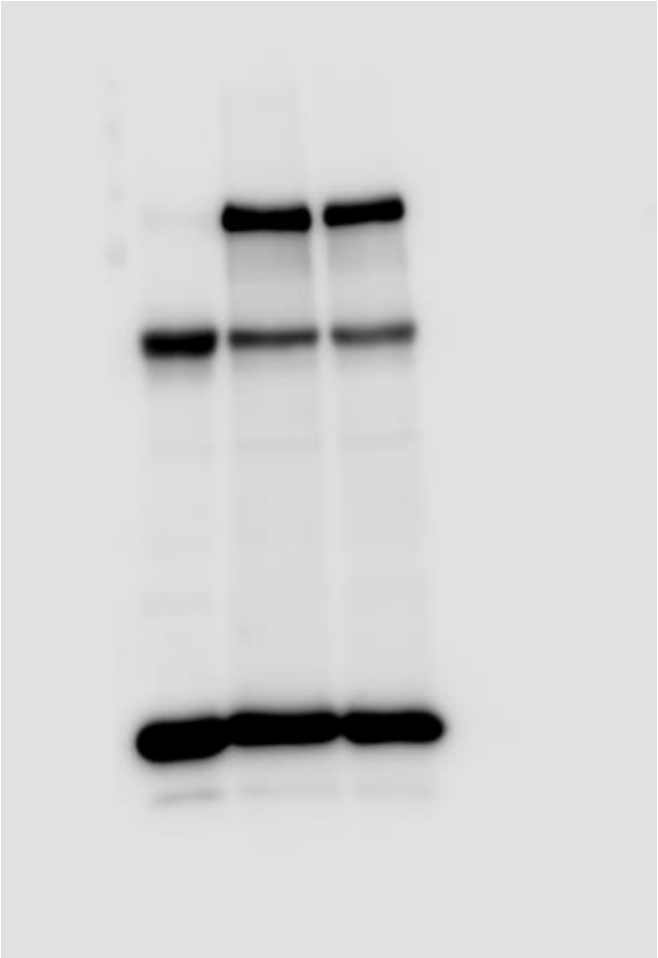

ACTIN

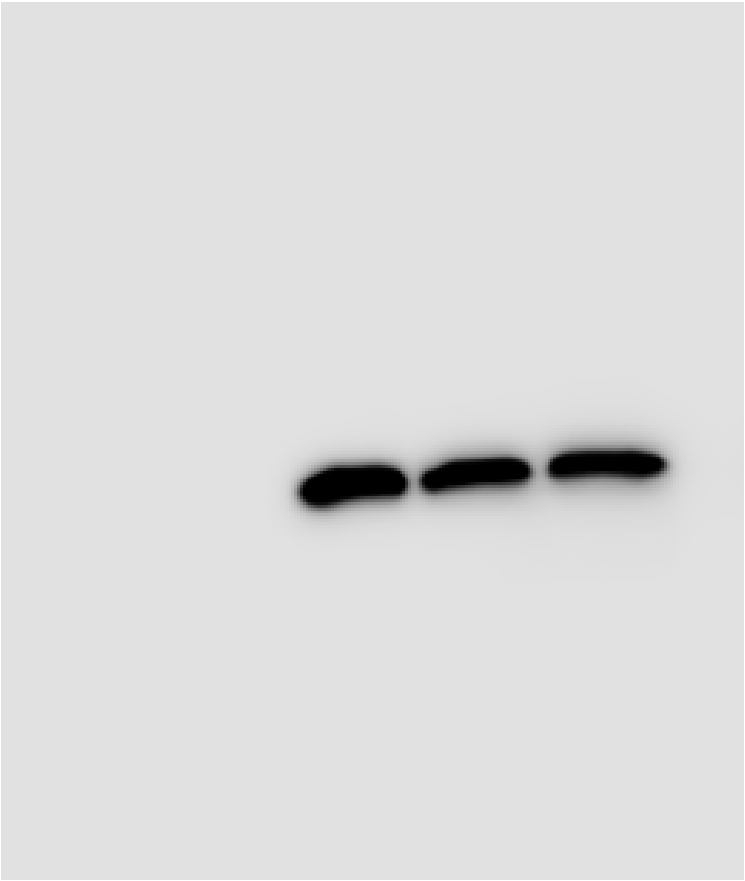

SQ/TQ

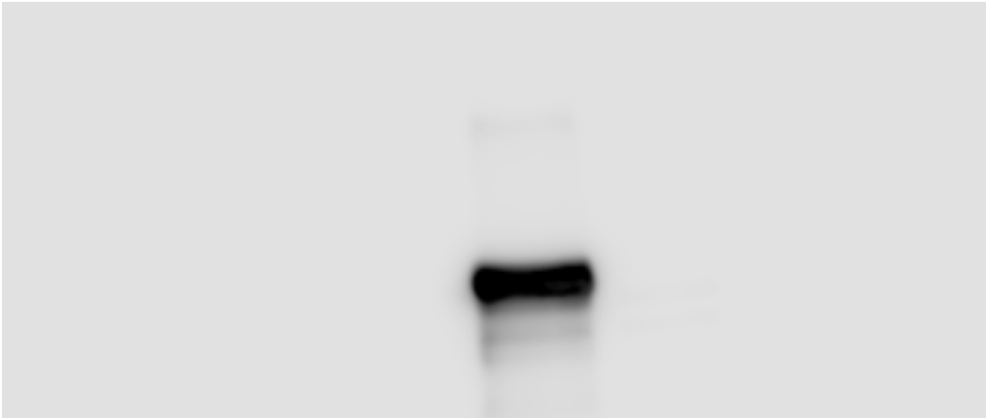

Figure 5C

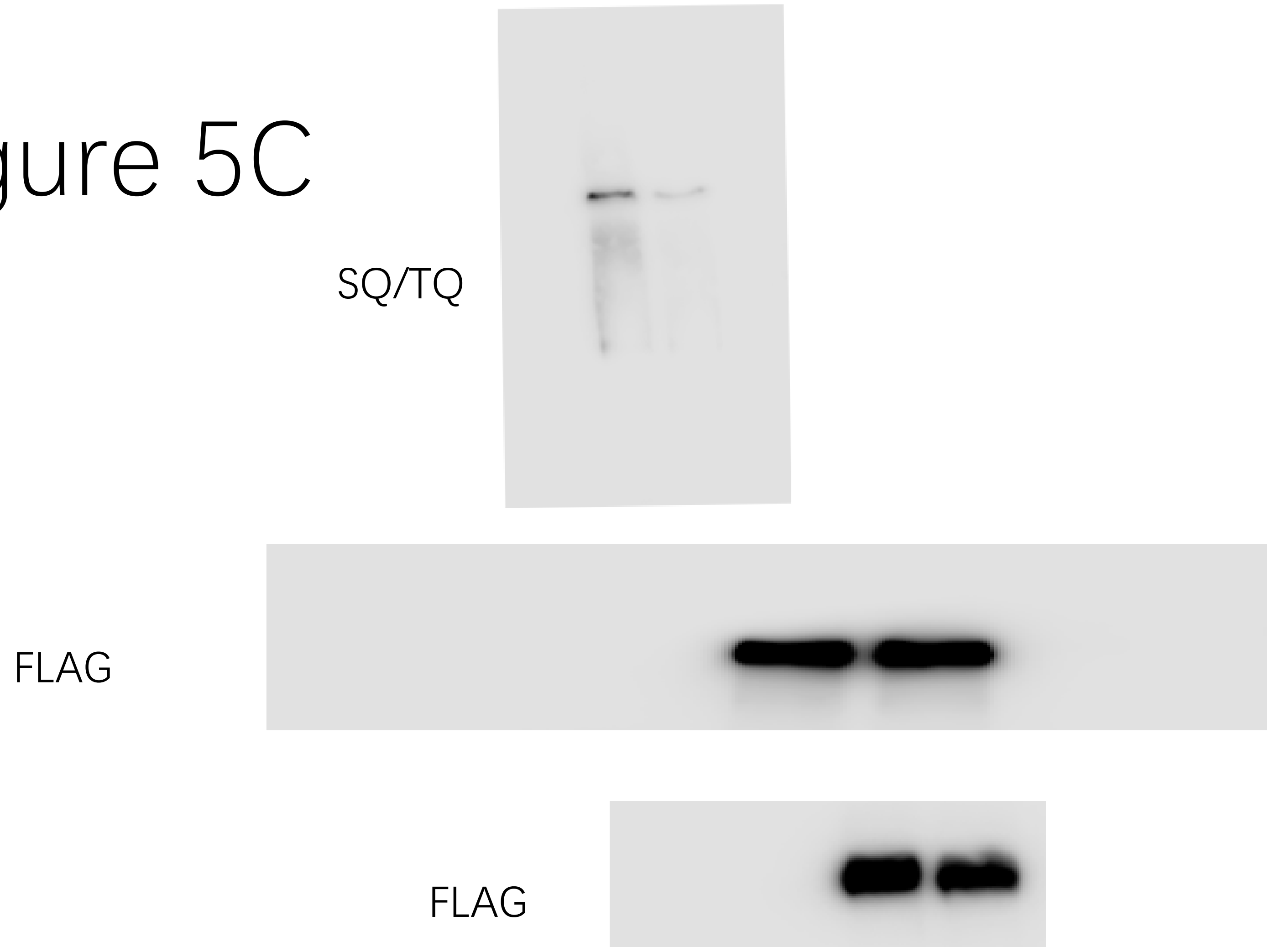

Figure 5E

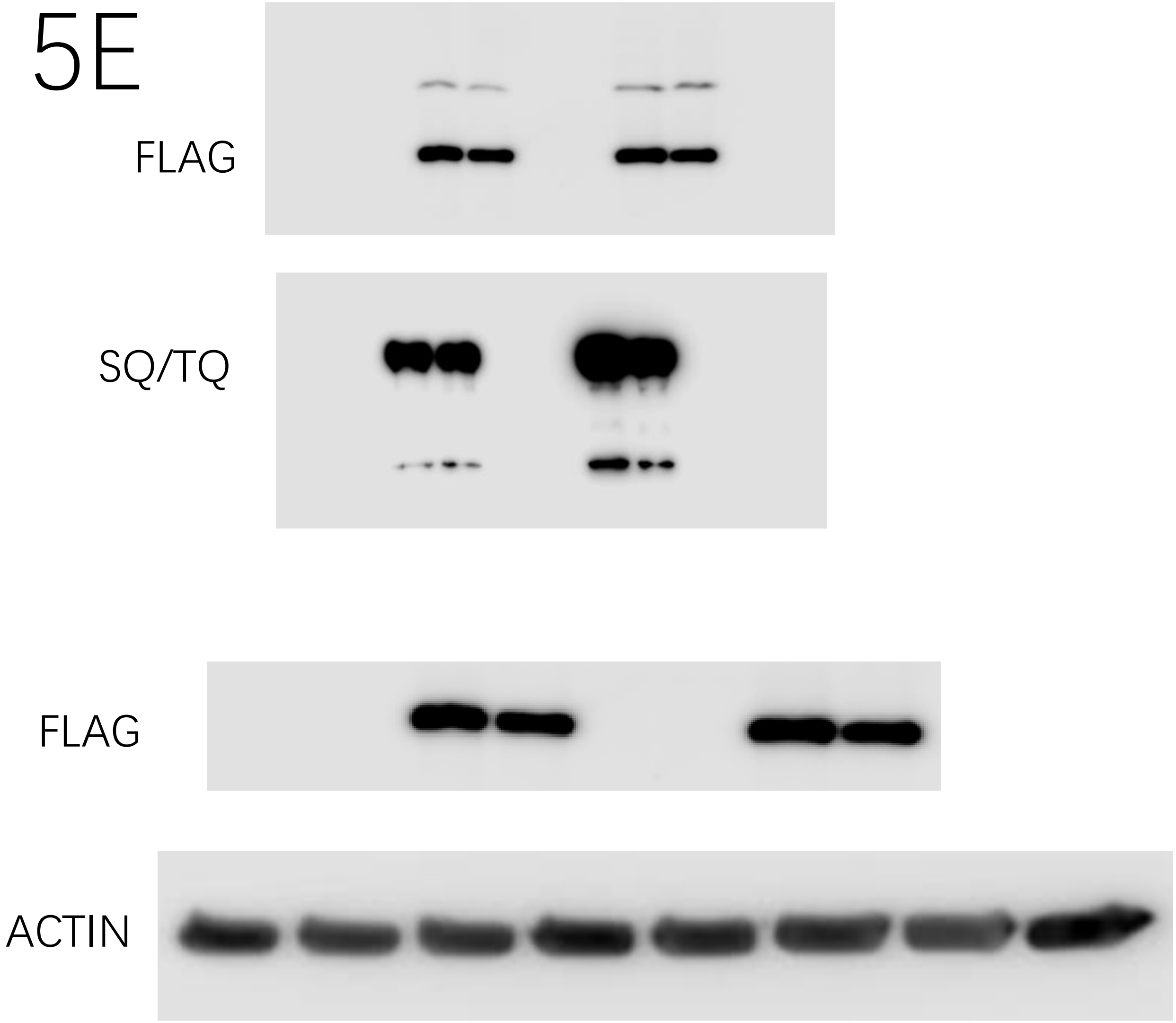

Figure 5G

SQ/TQ

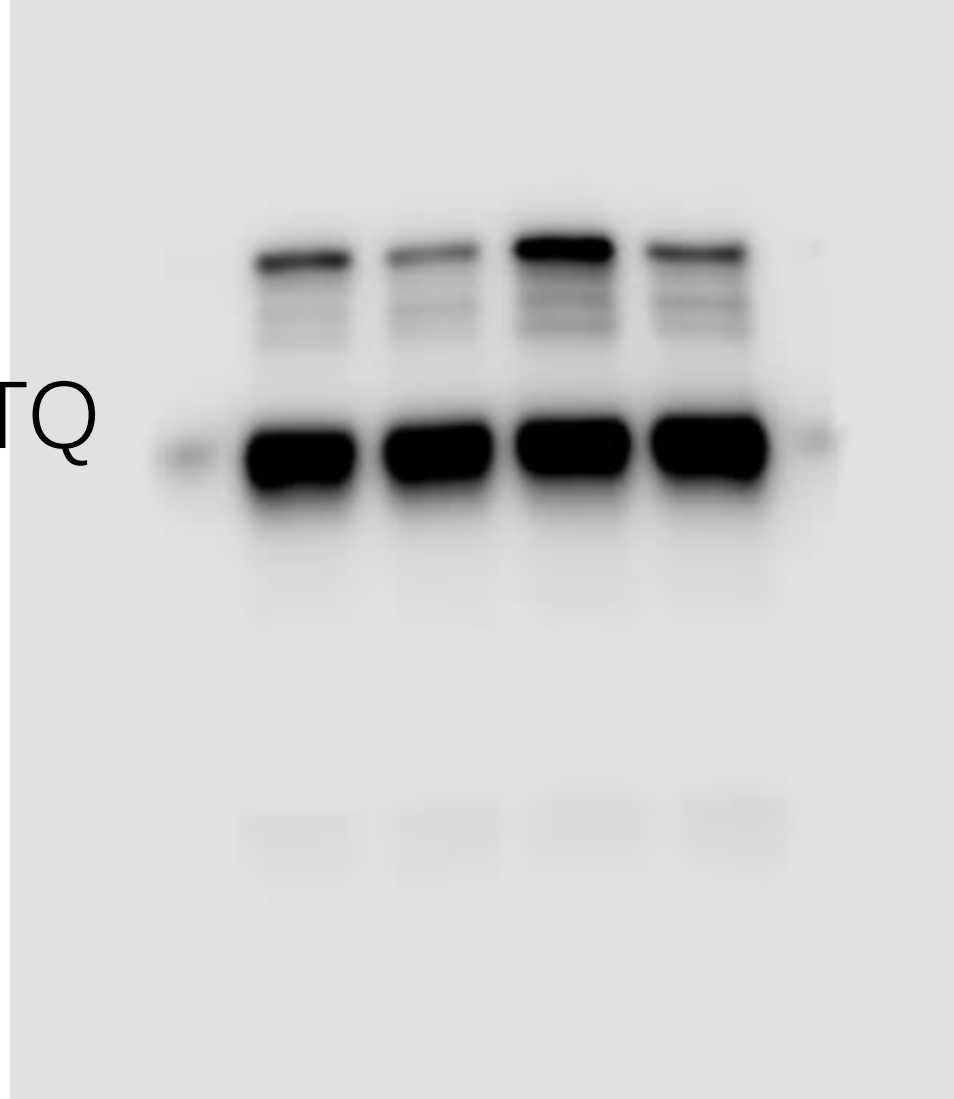

SUN2

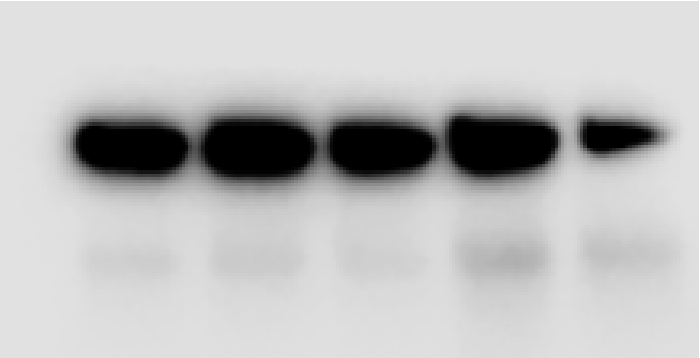

SUN2

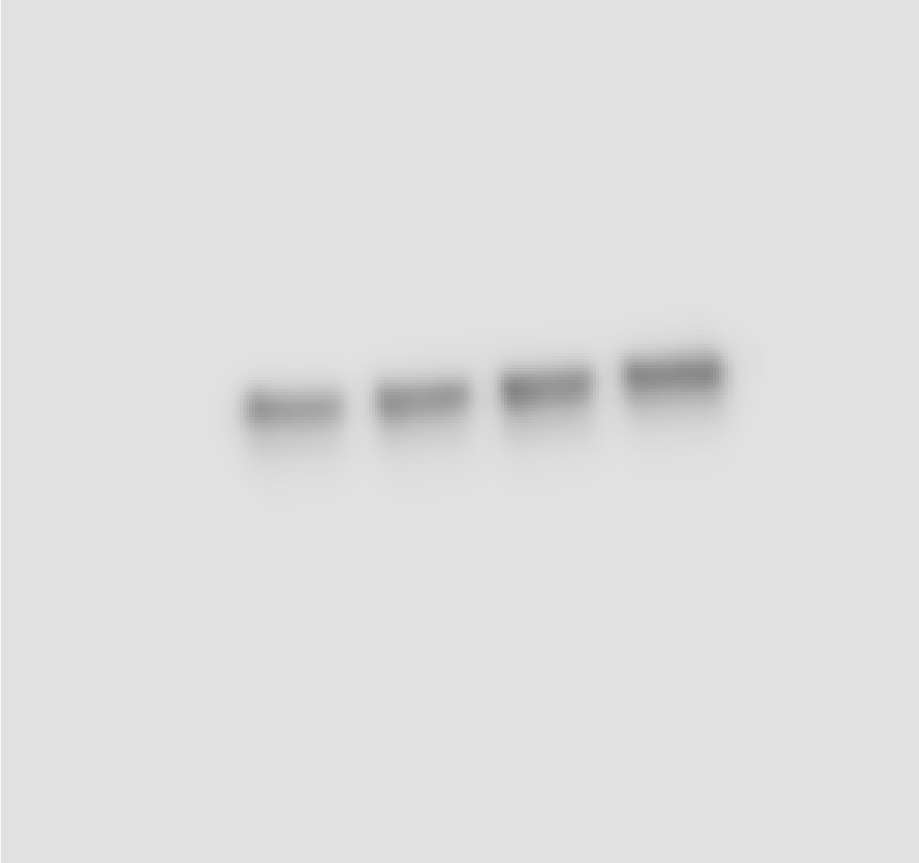

ACTIN

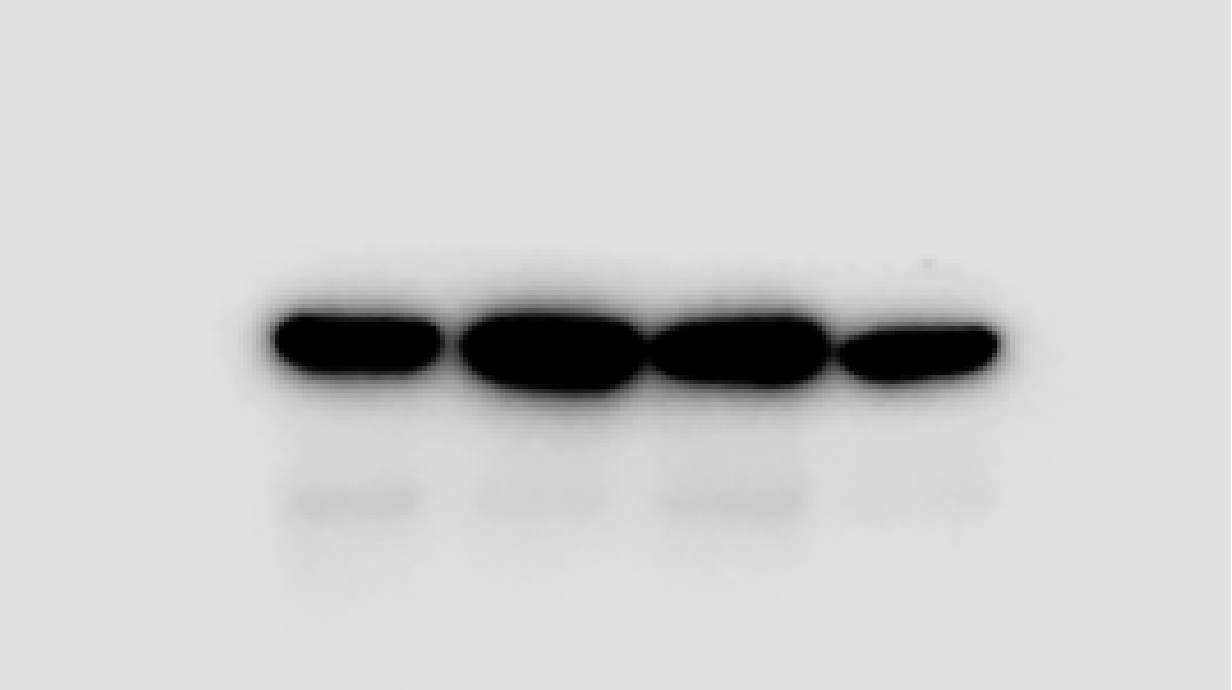

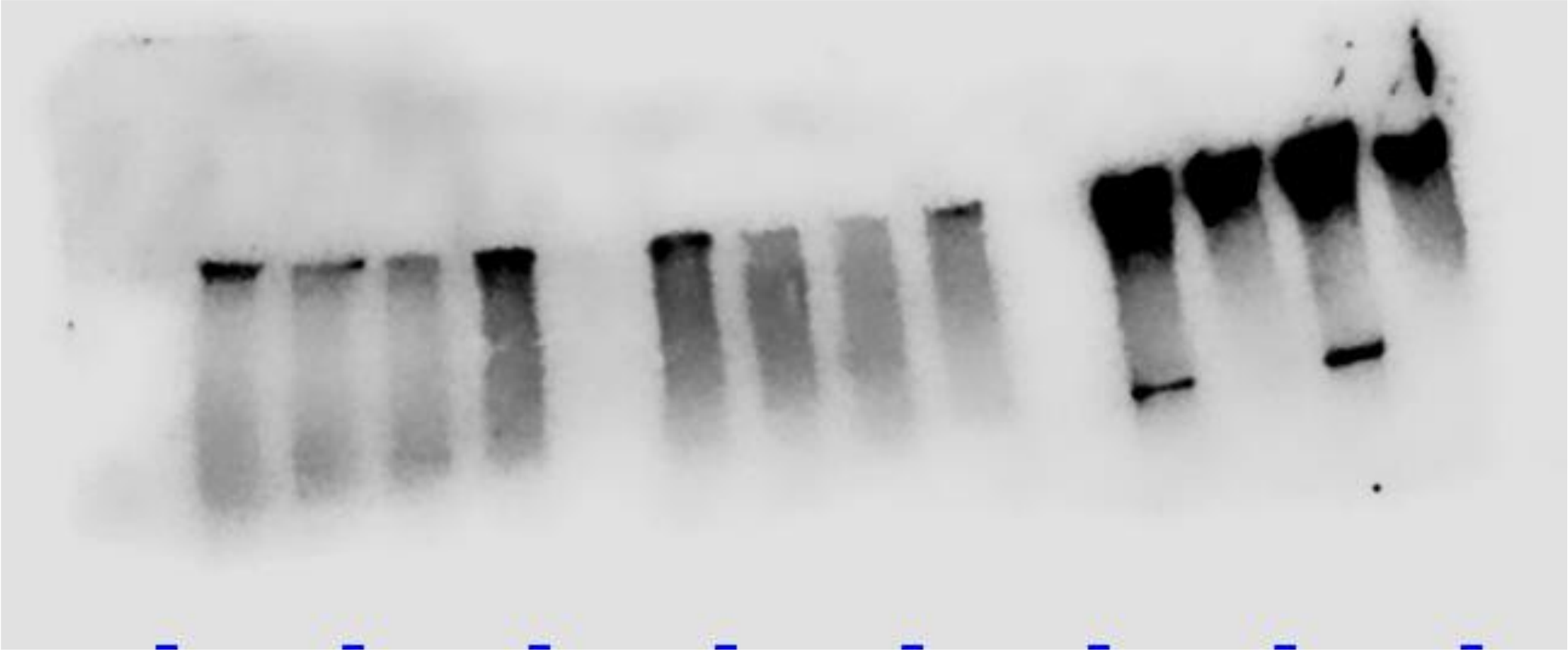

ATR

Figure 5K

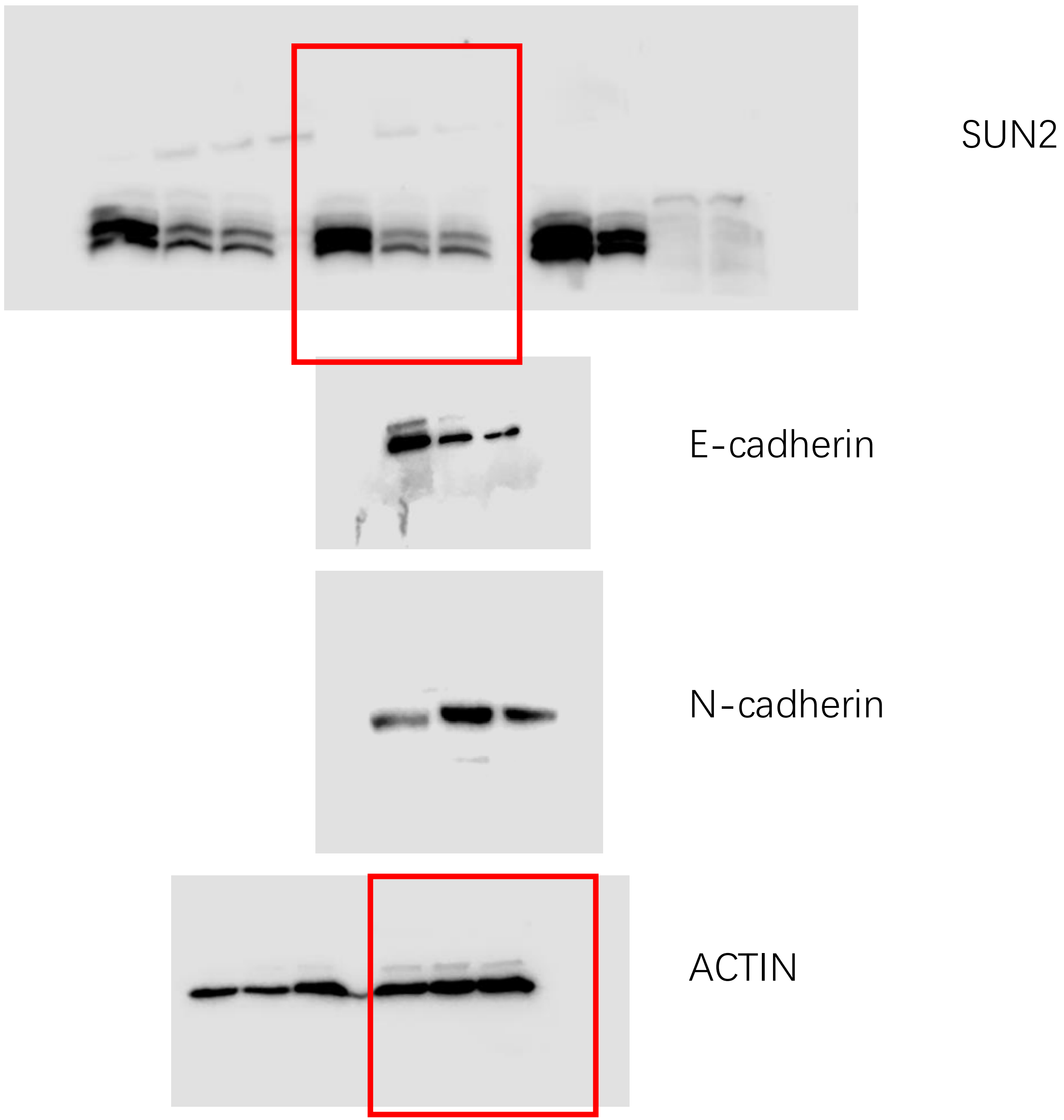

Figure 5M

N-cadherin

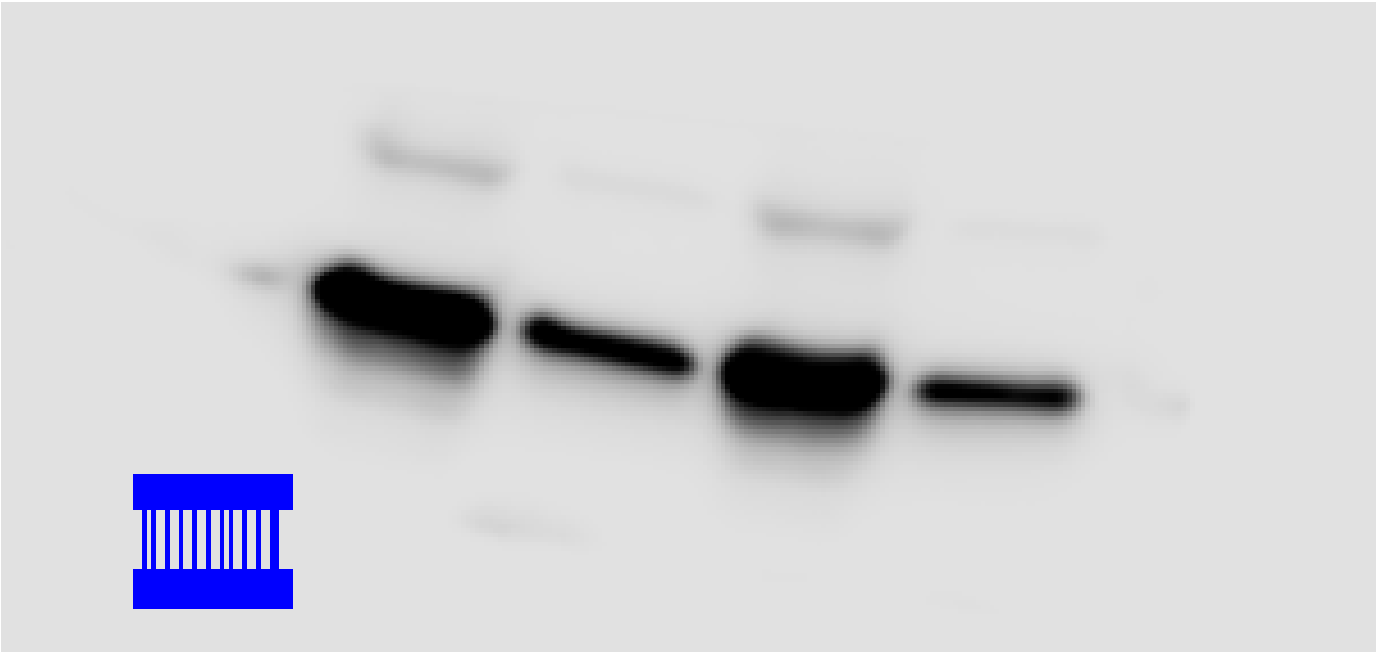

SUN2

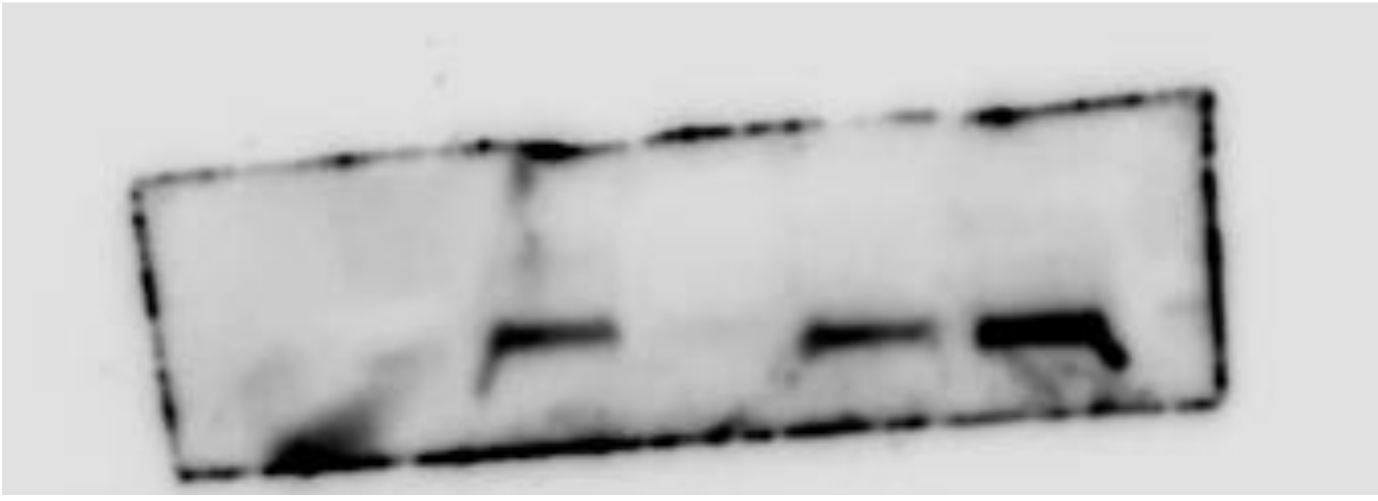

E-cadherin

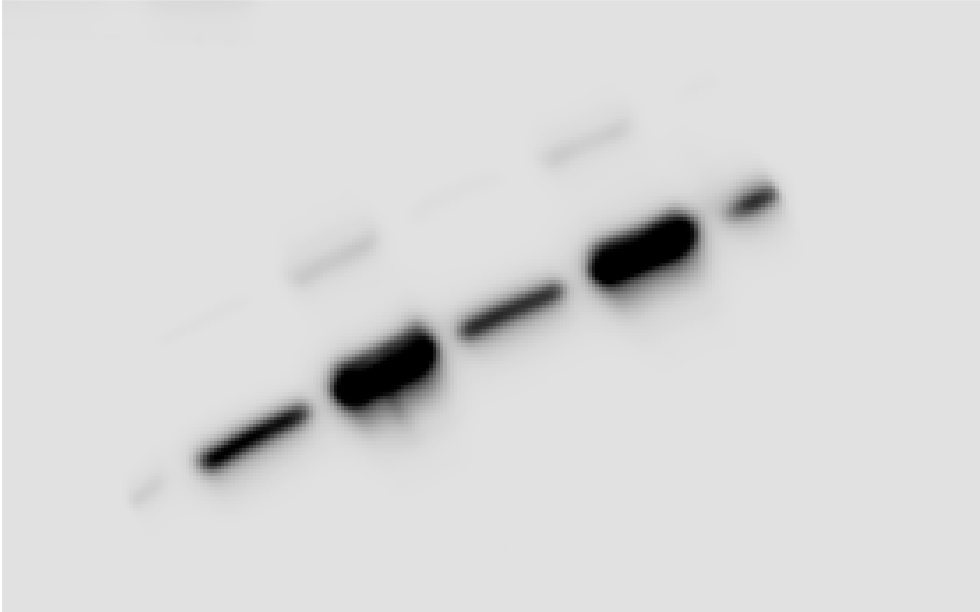

GAPDH

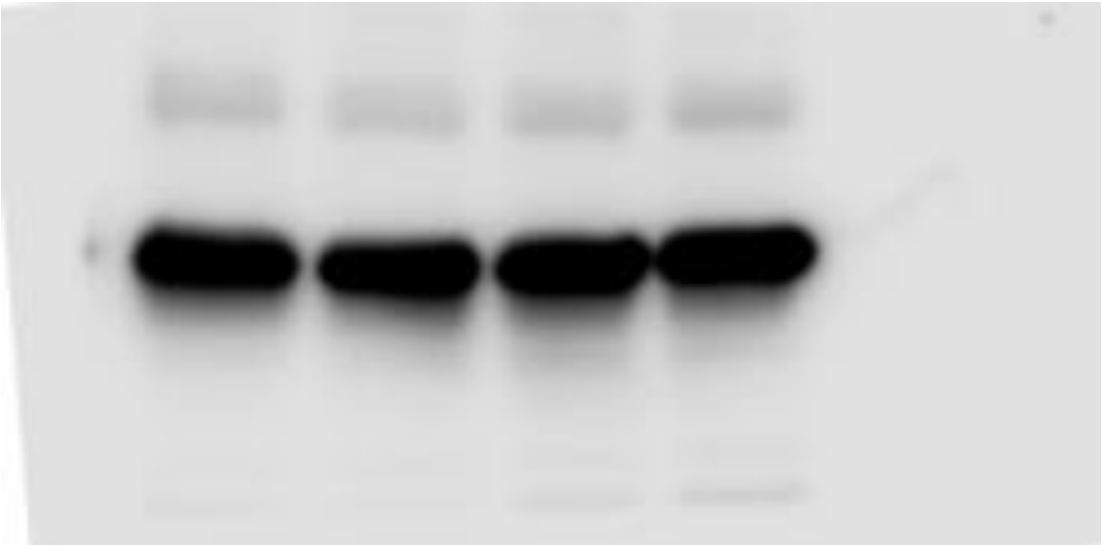

FLAG

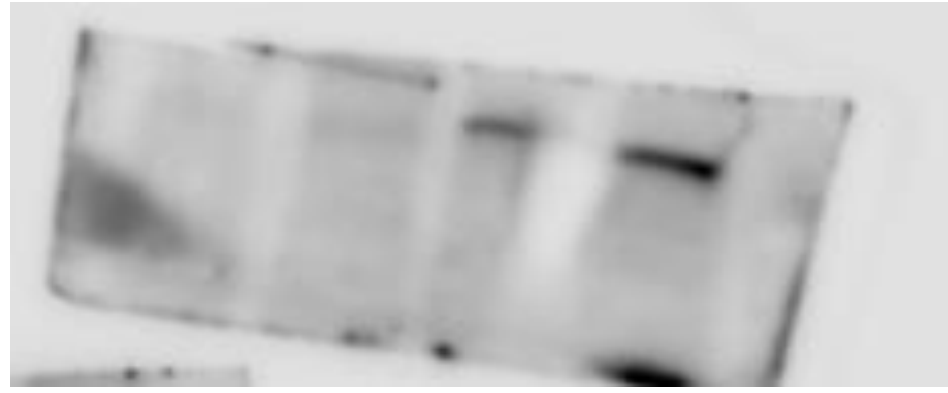

Figure 6A-B

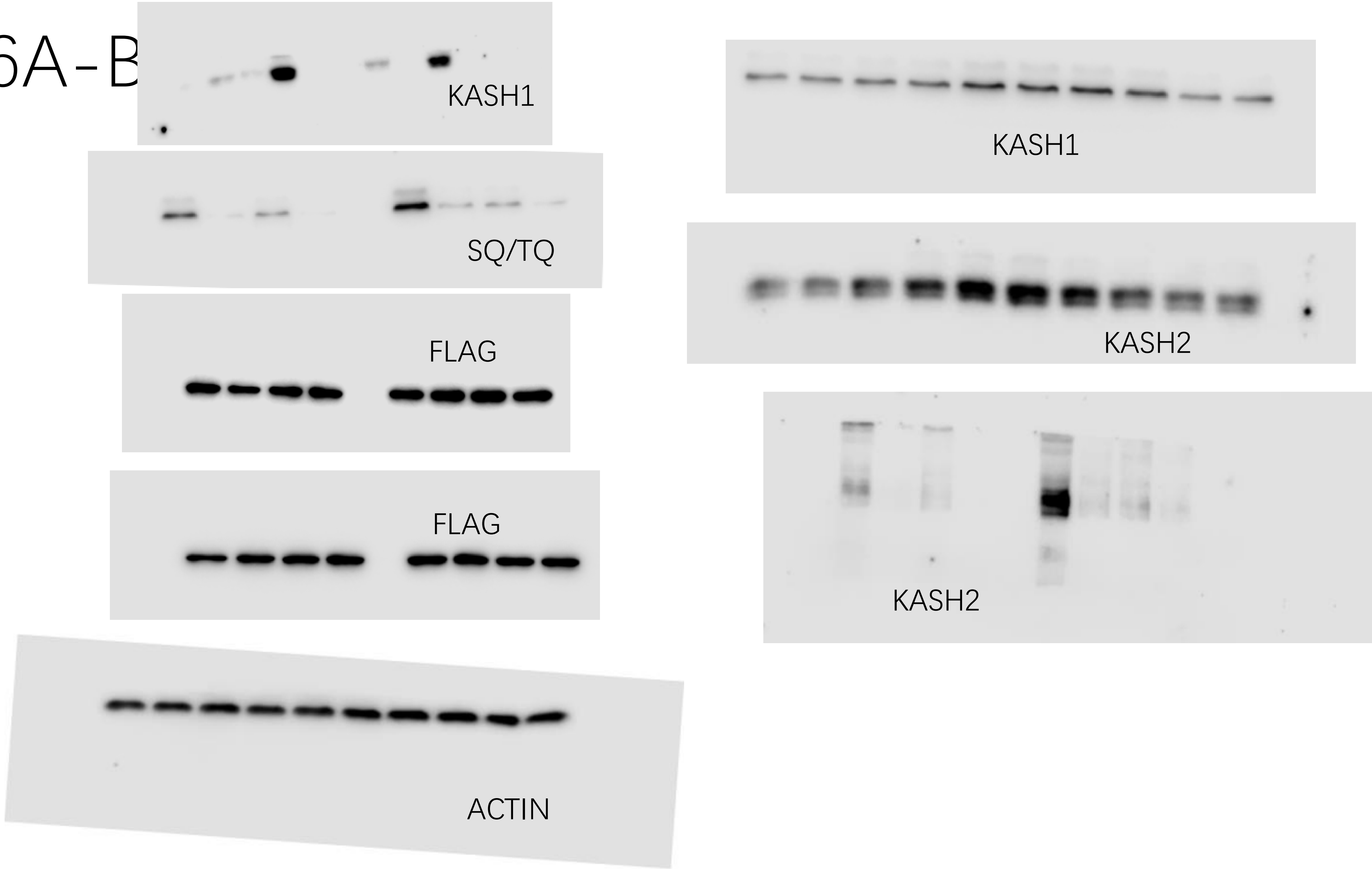

Figure 6D

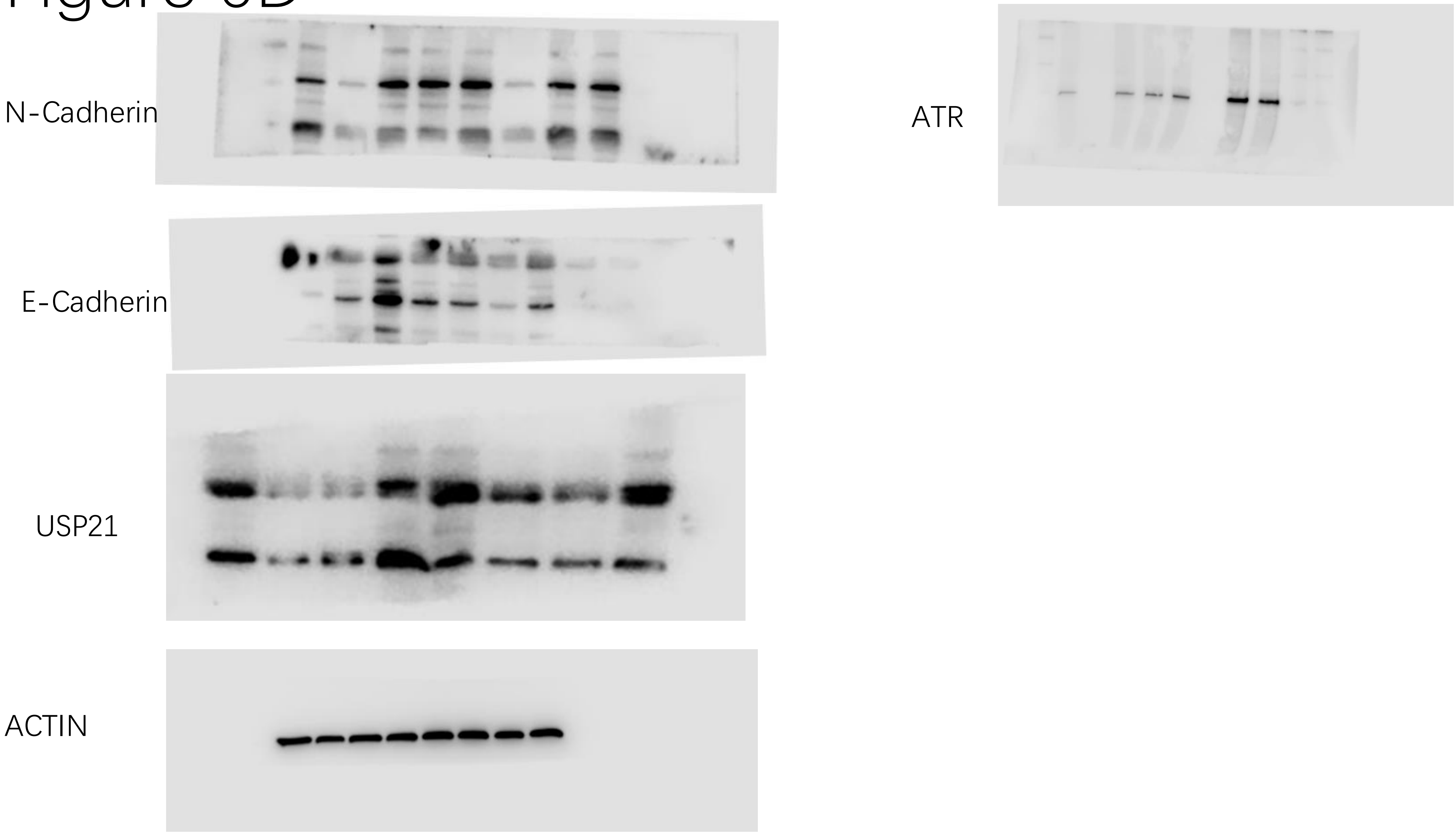

7A

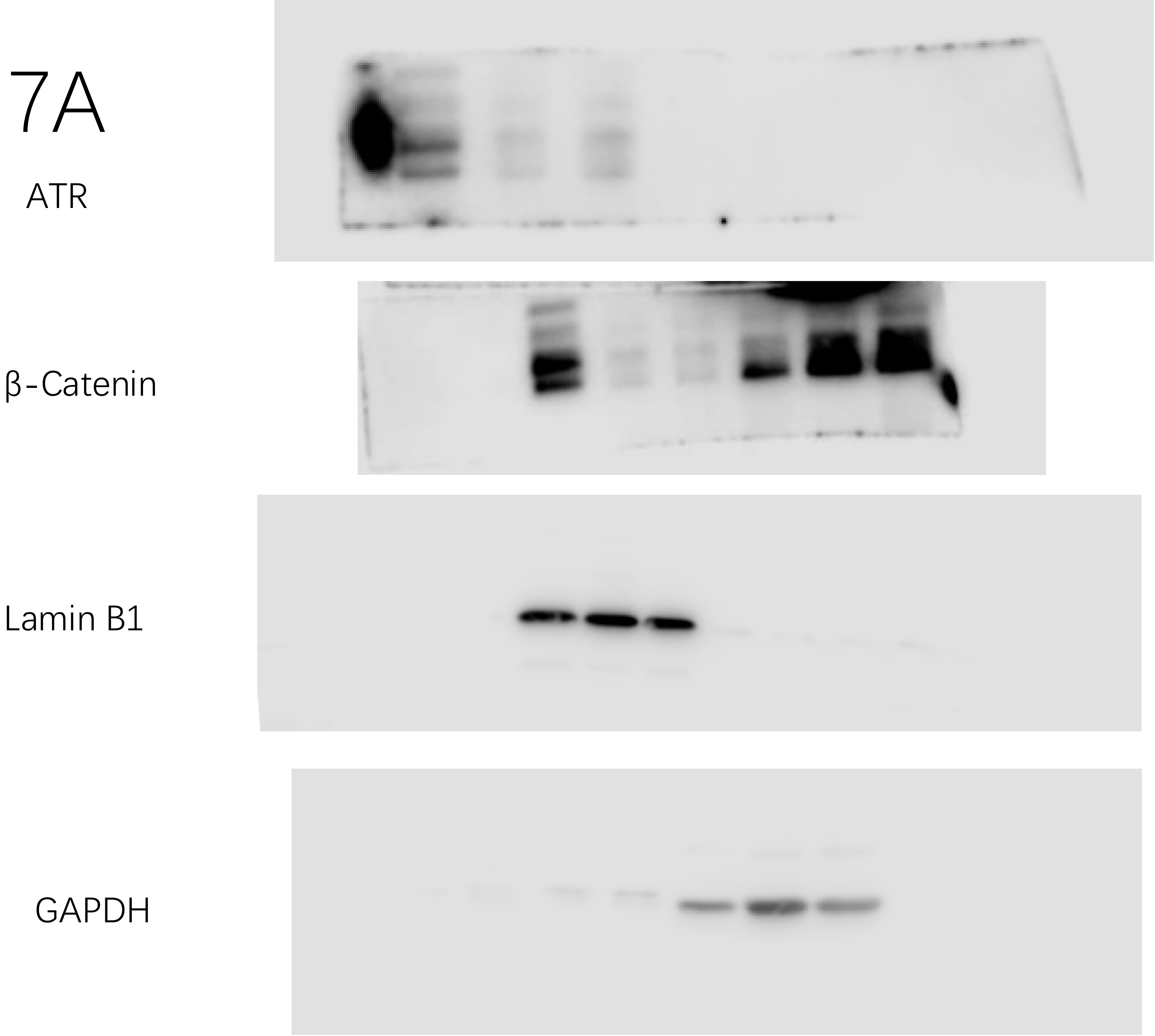

Figure 7C

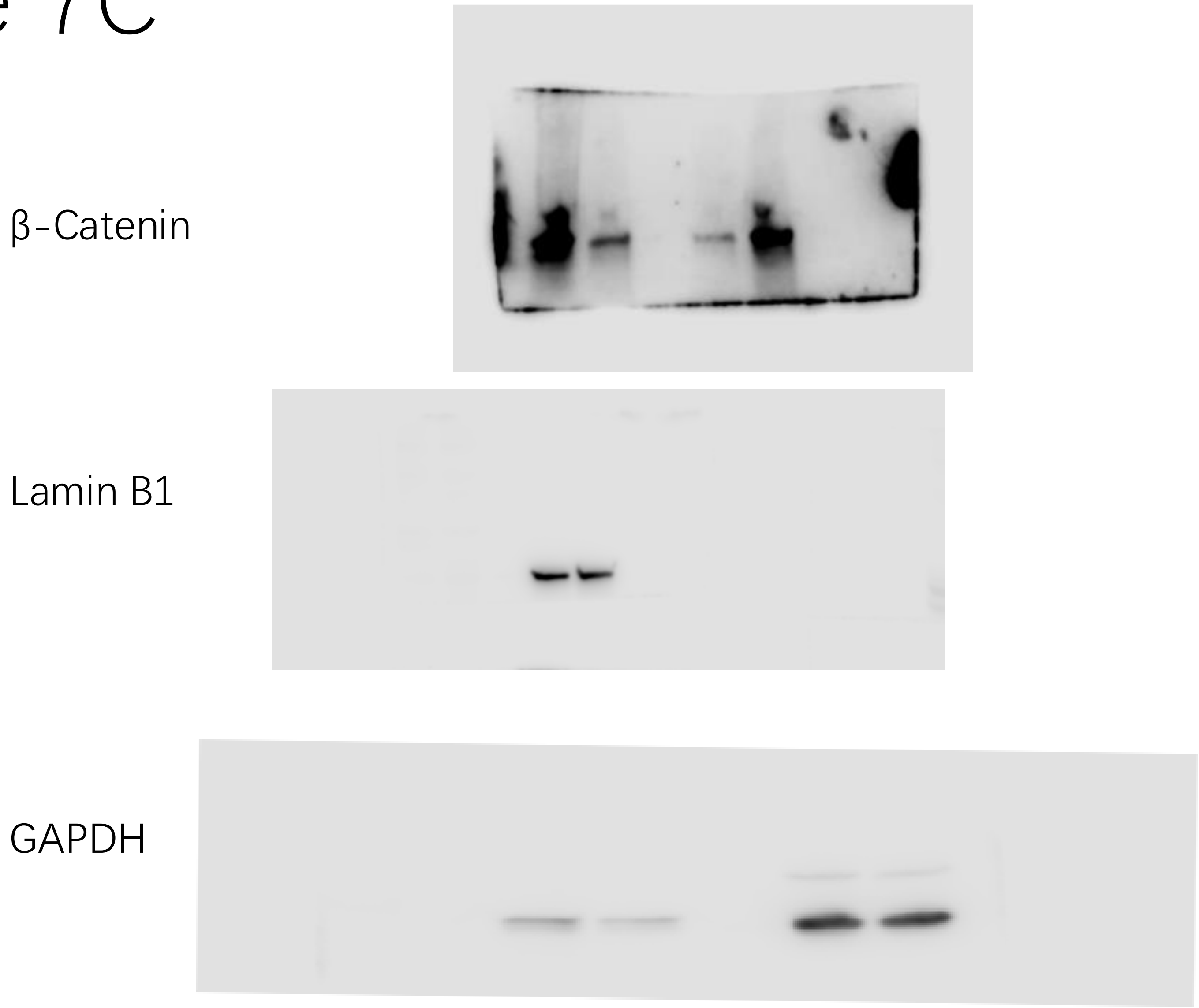

7E

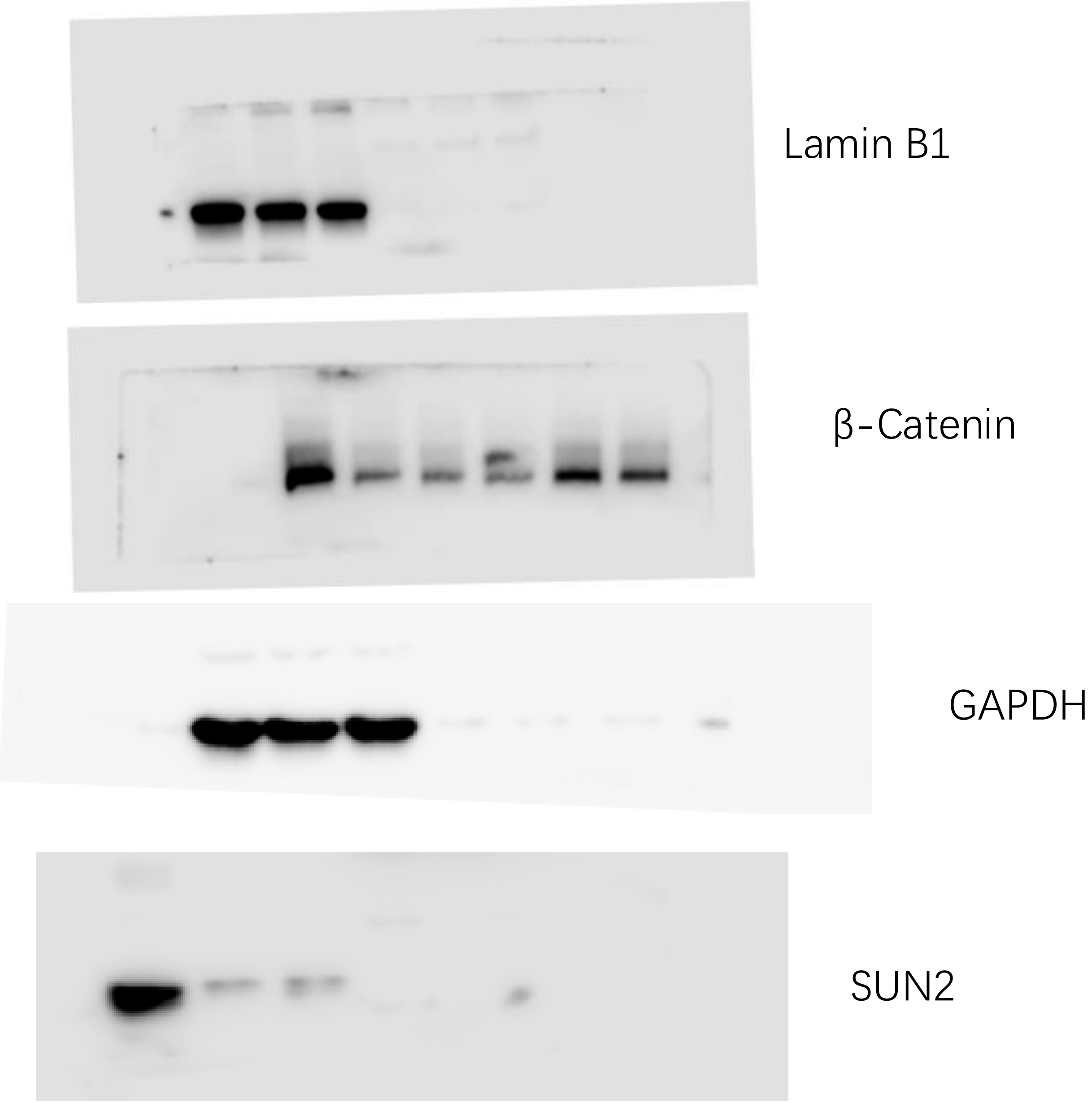

Figure 7G

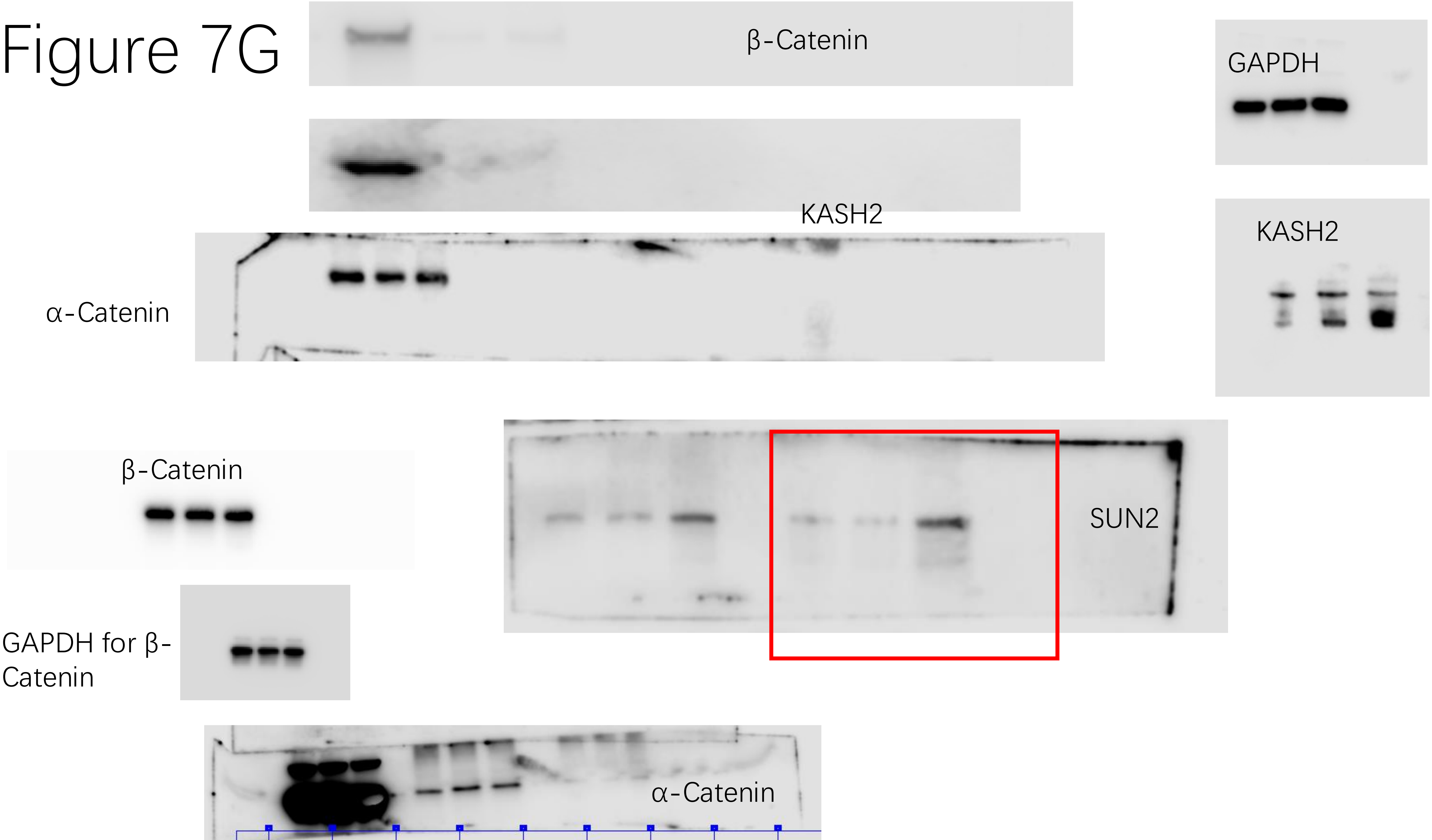
β-Catenin
GAPDH
KASH2
α-Catenin
KASH2
β-Catenin
SUN2
GAPDH for β-Catenin
α-Catenin

Figure 7I

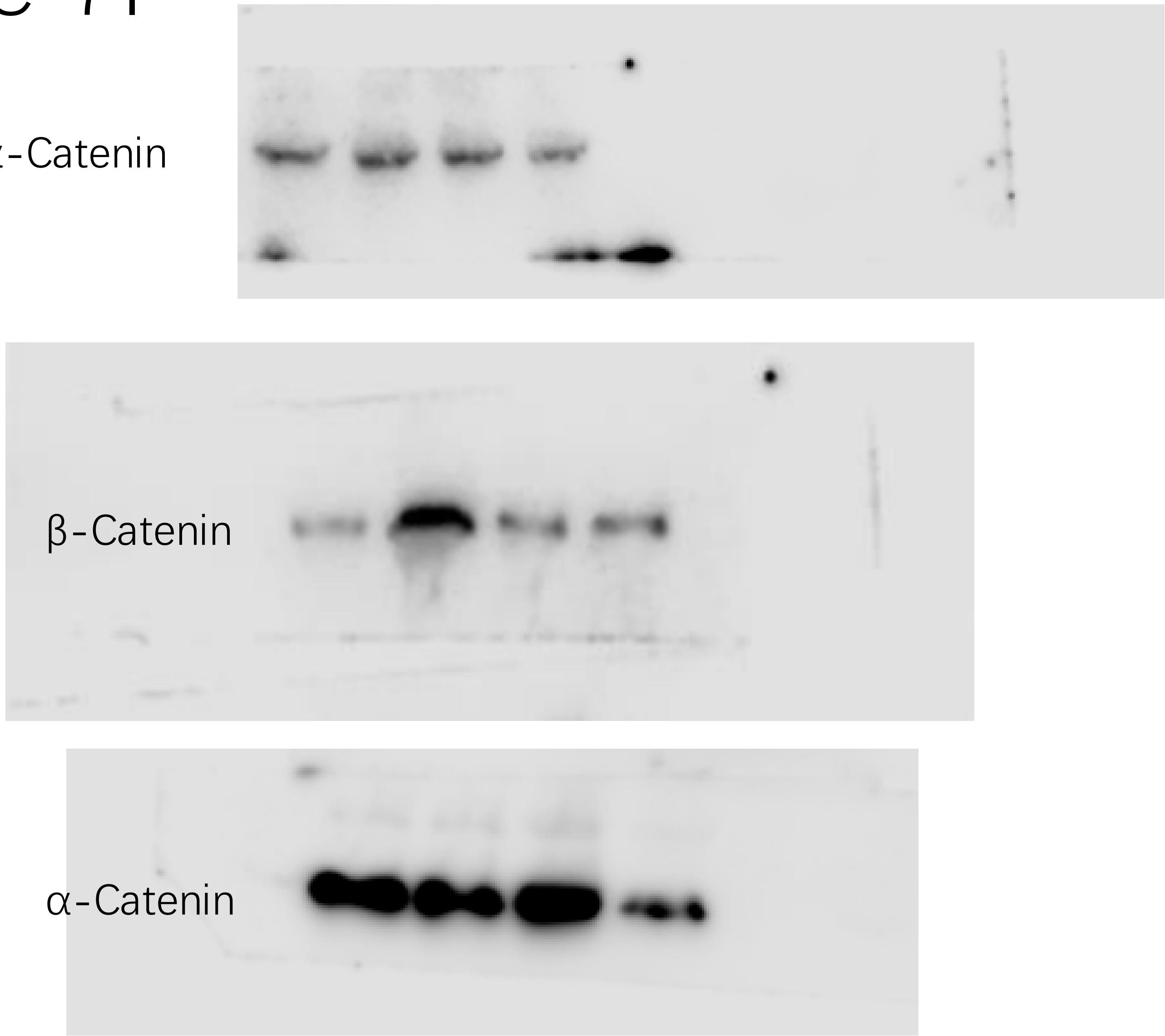

Figure 7P

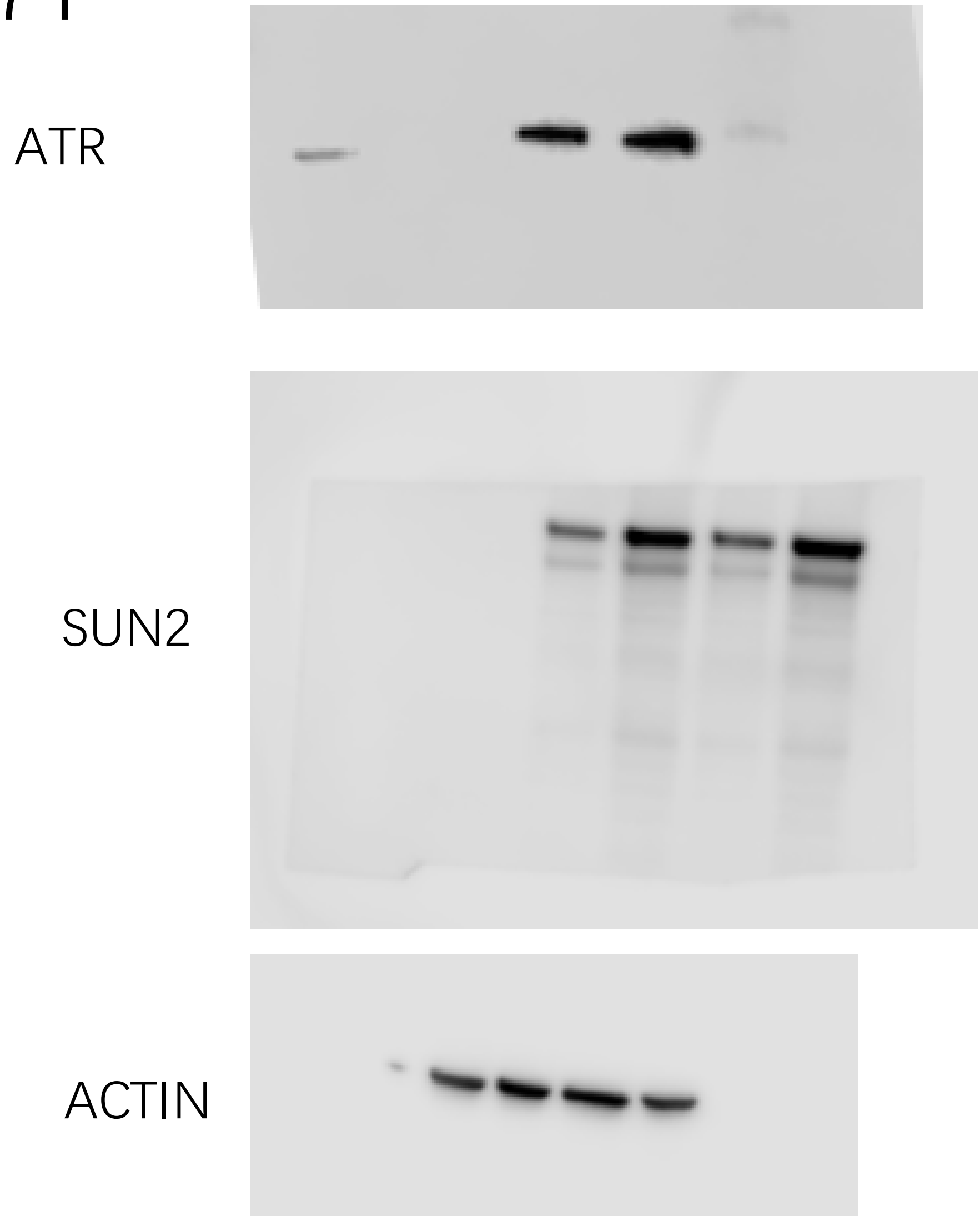

Figure 8D

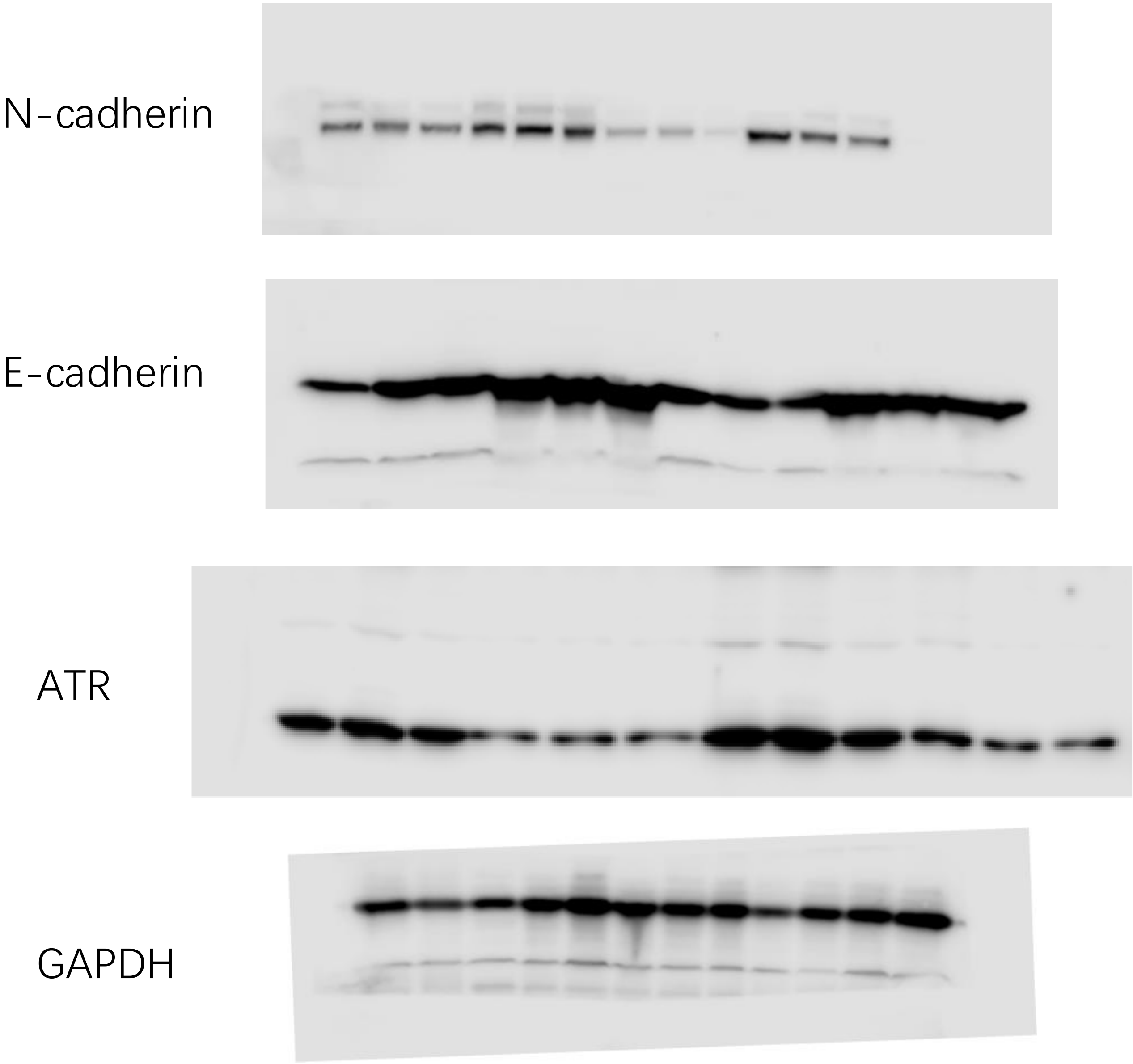

S1b

ATR

ACTIN

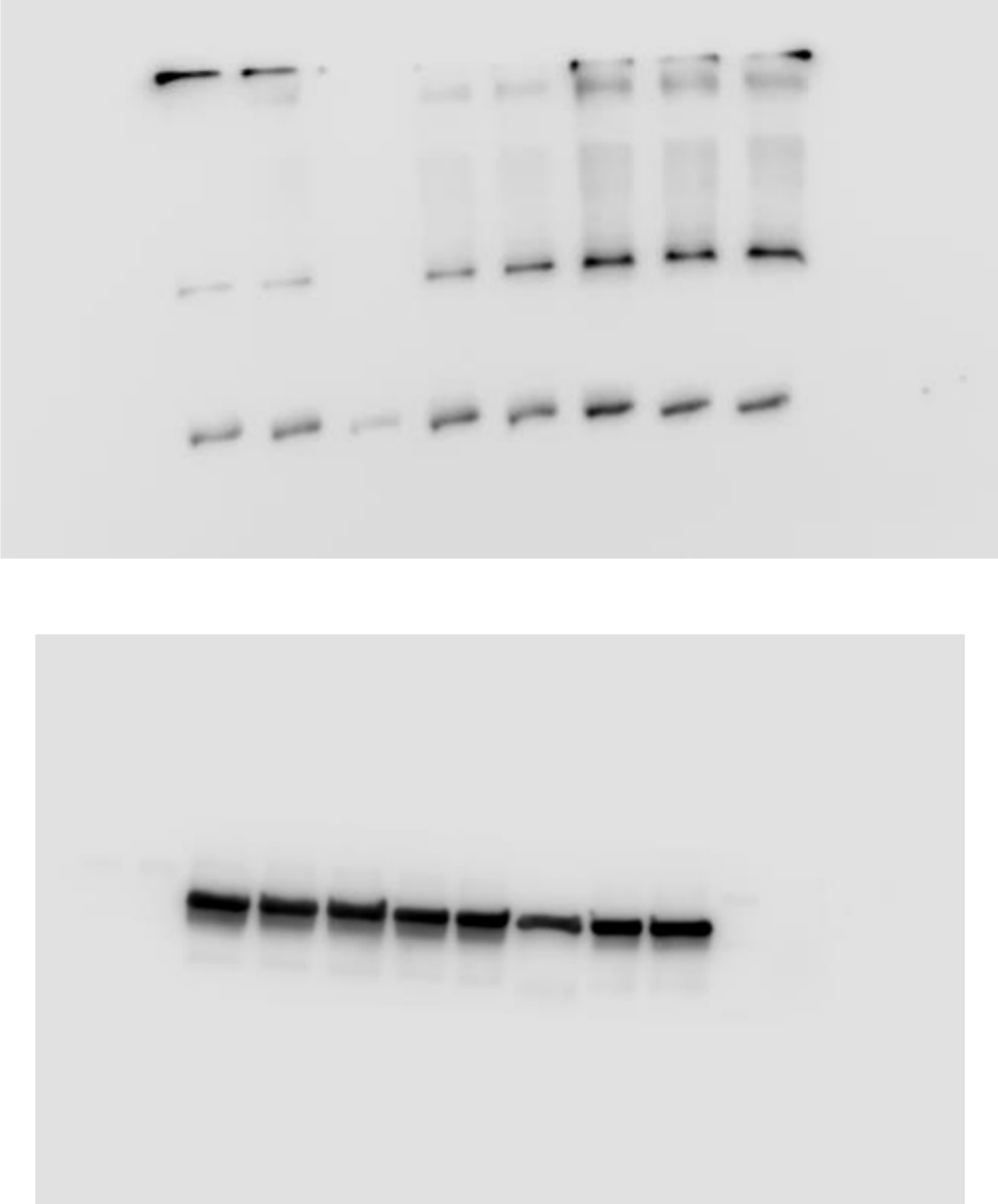

S1J

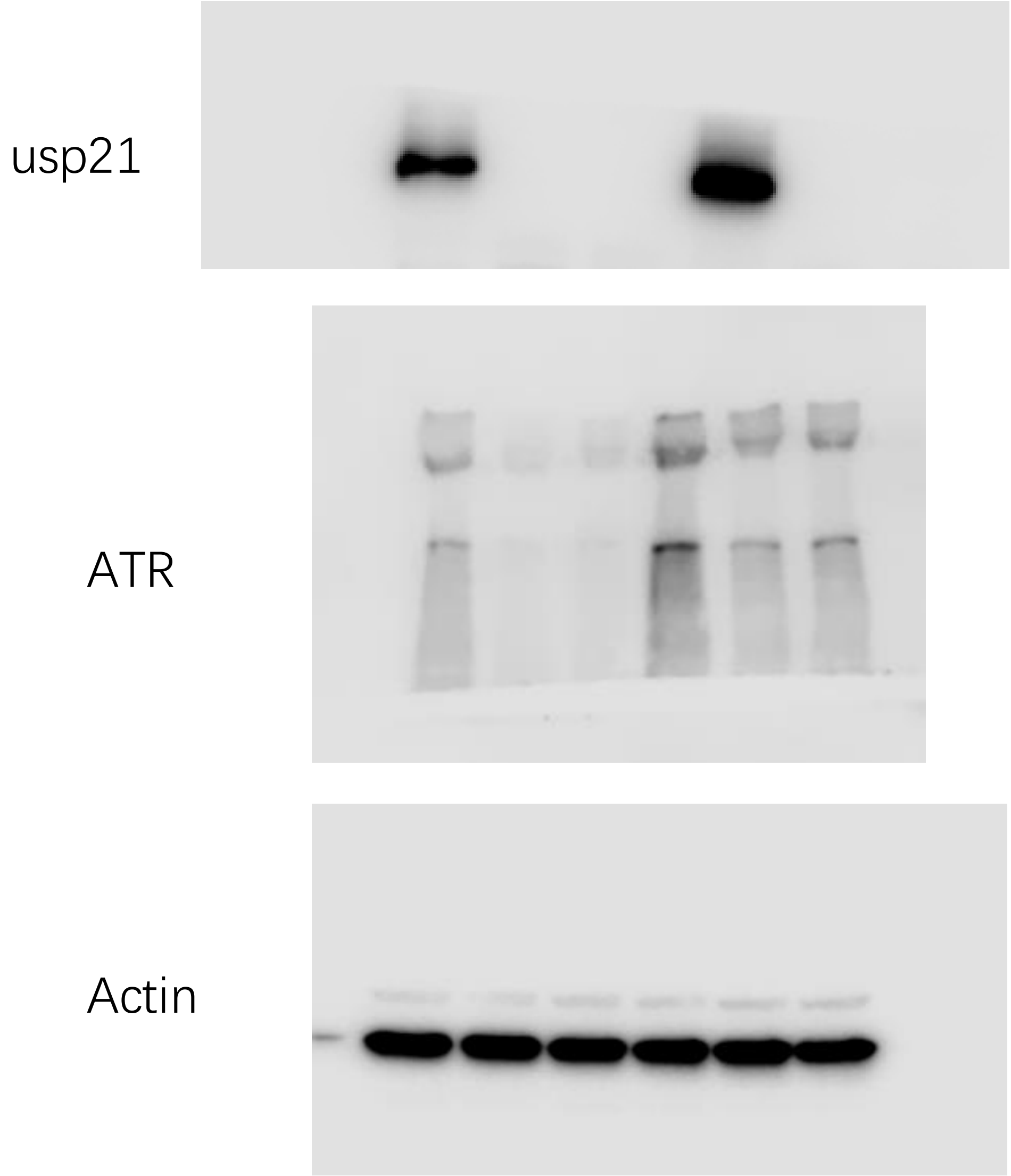

S1L

USP21

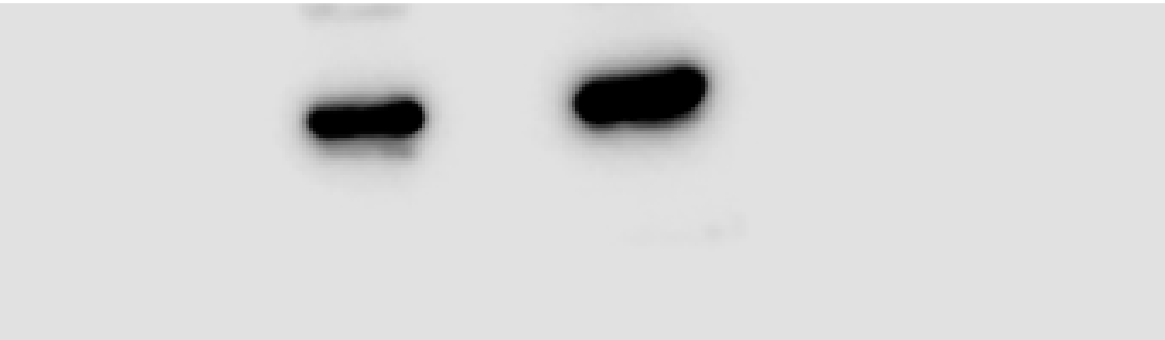

ATR

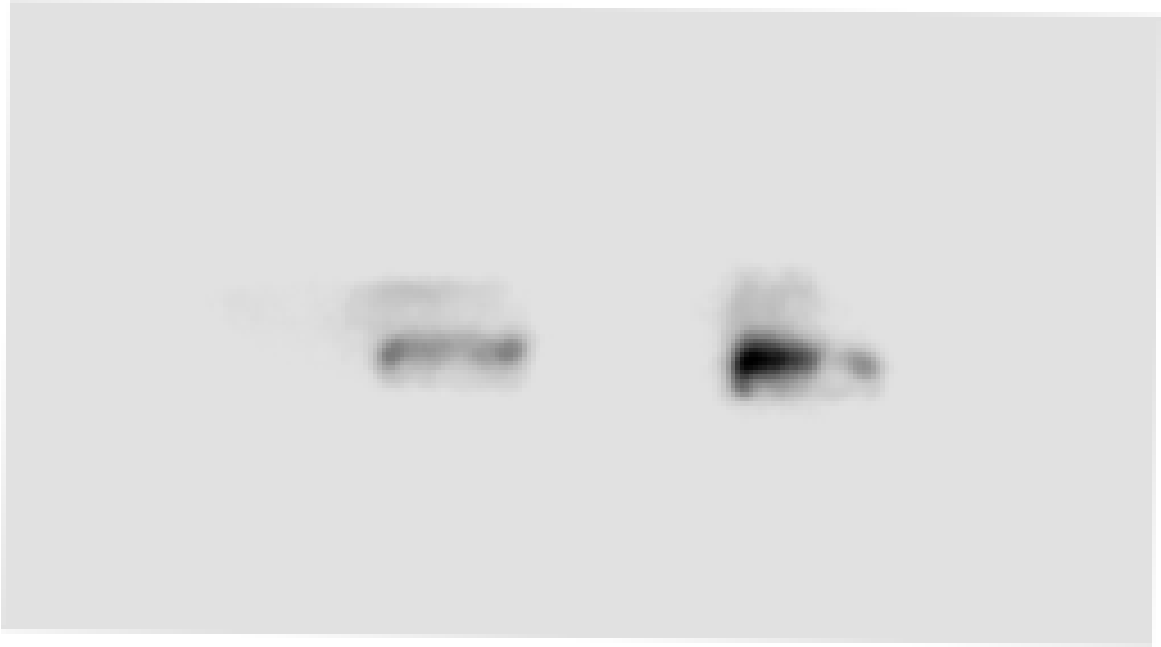

S1M

FLAG

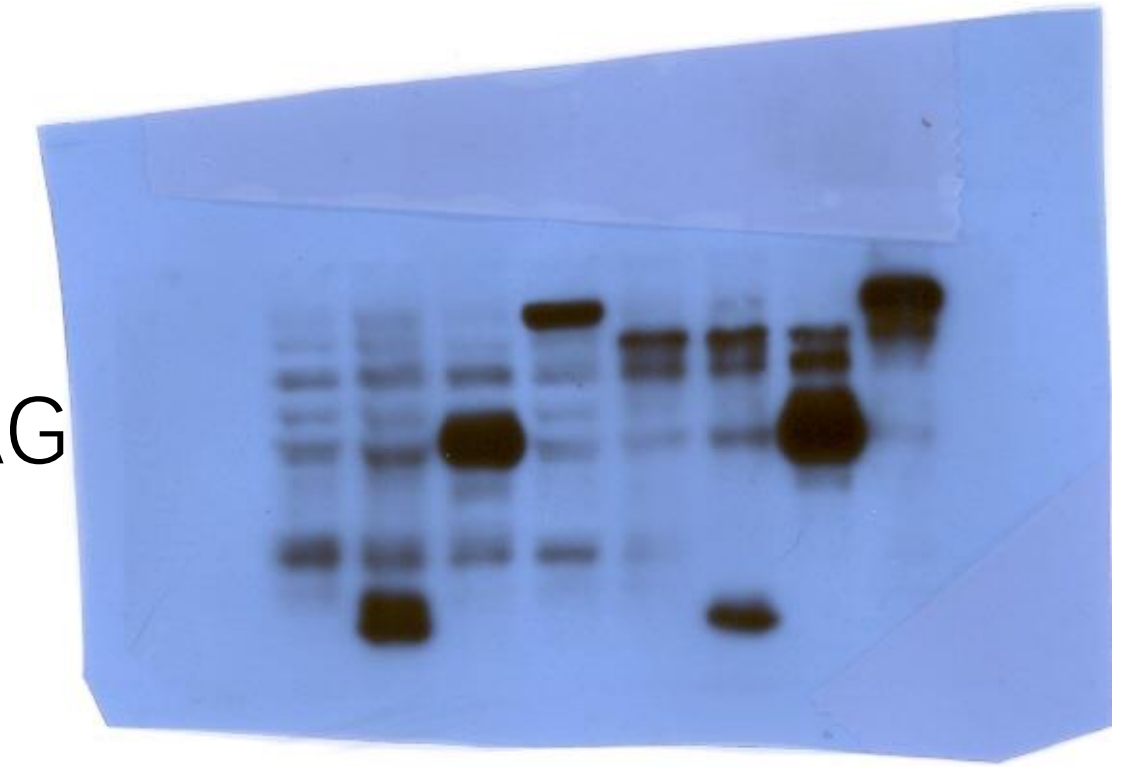

ATR

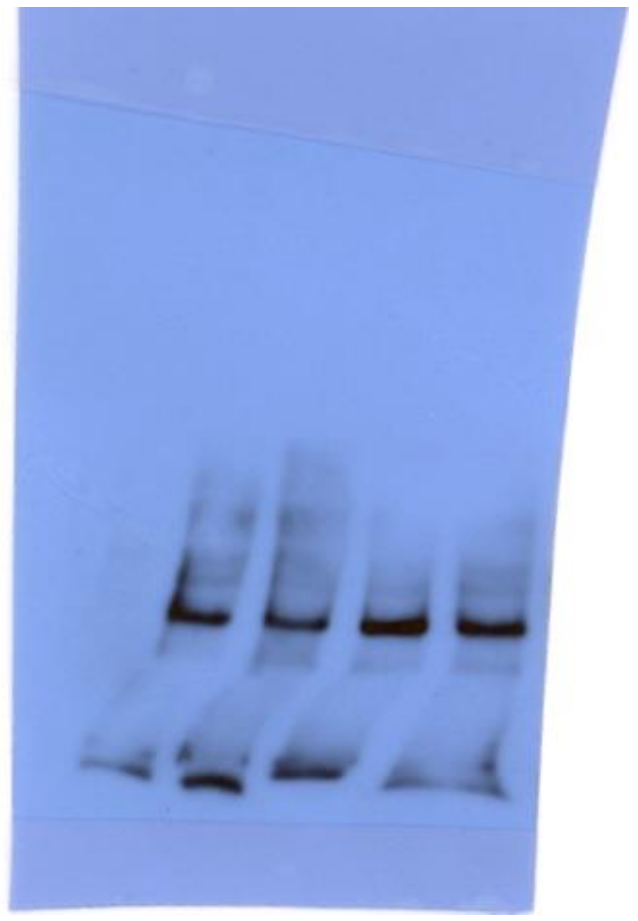

ATR

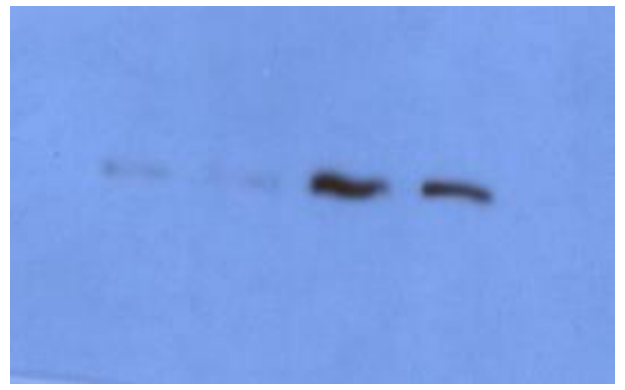

Actin

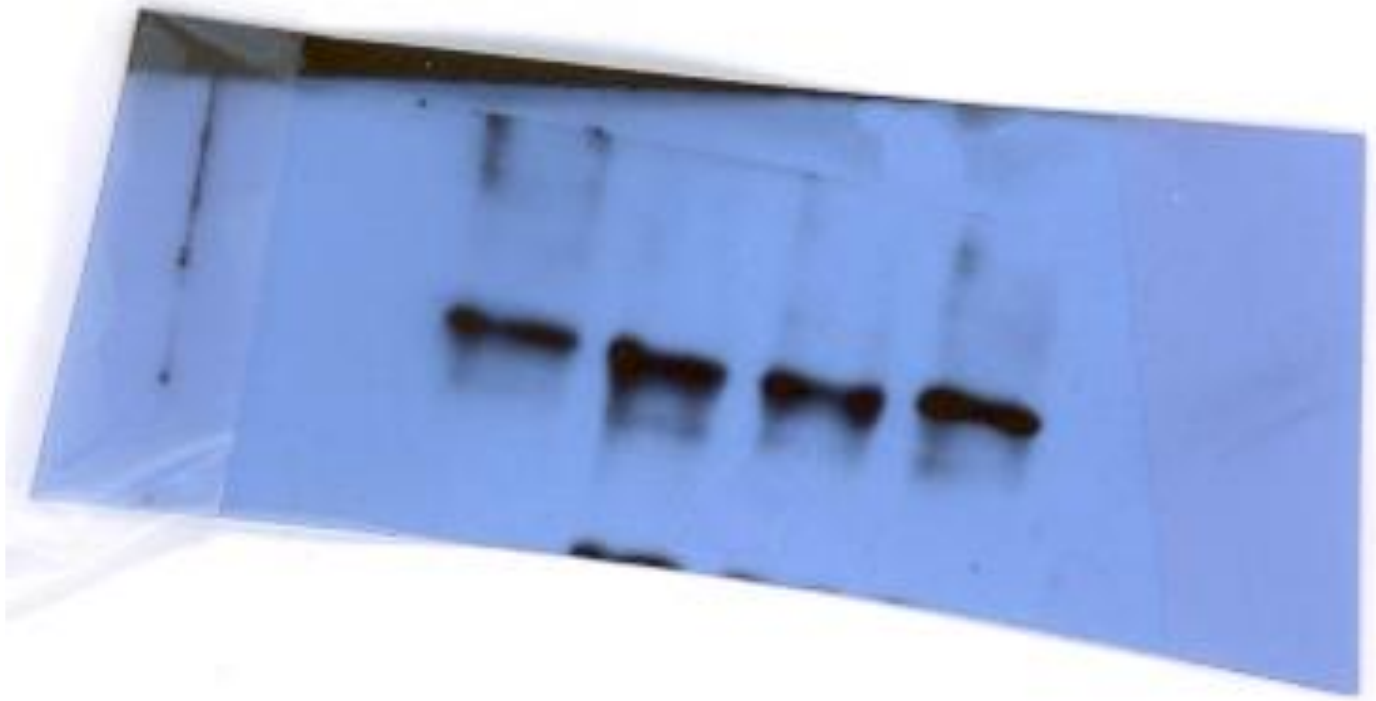

S1O

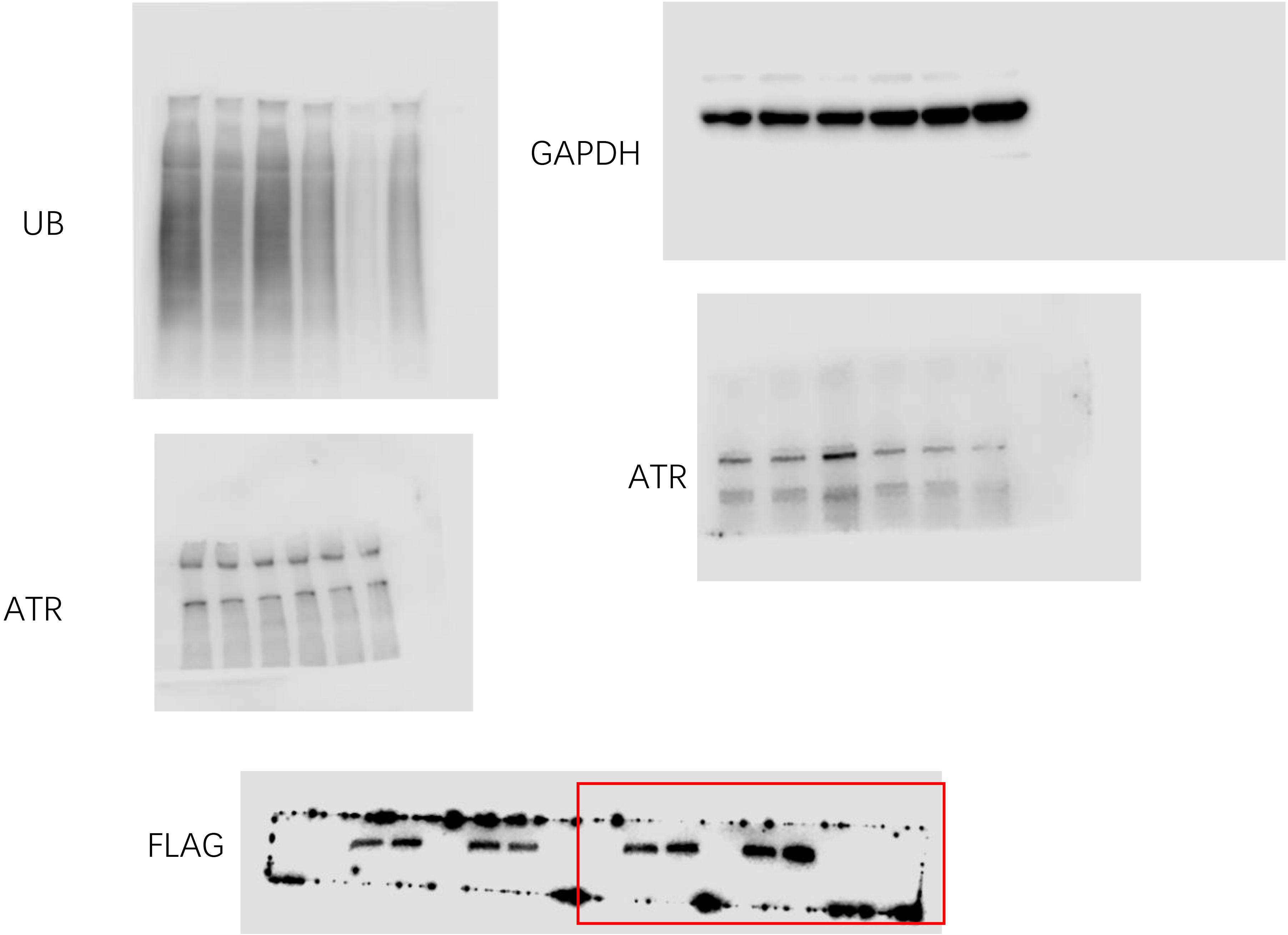

Figure S3A

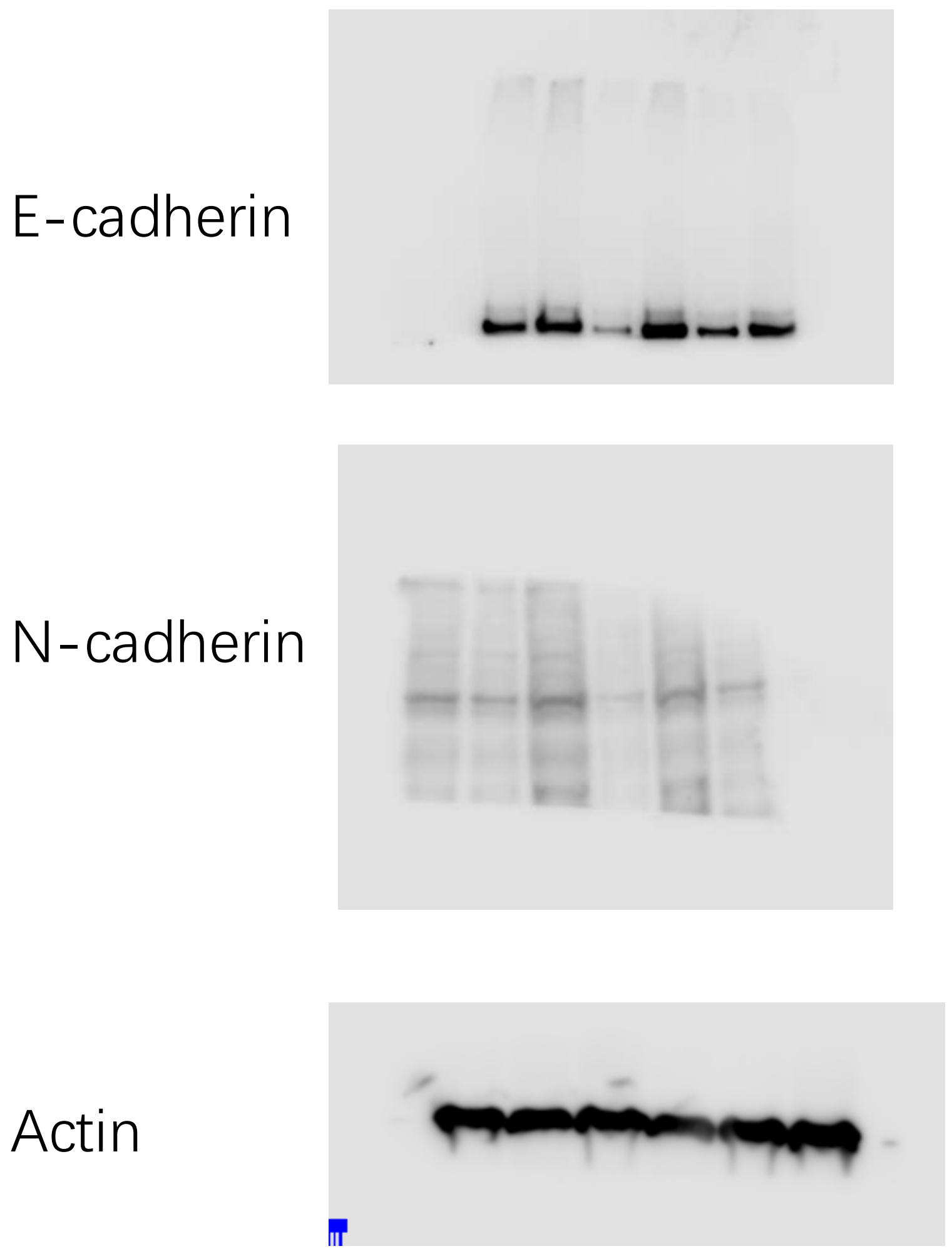

S3C

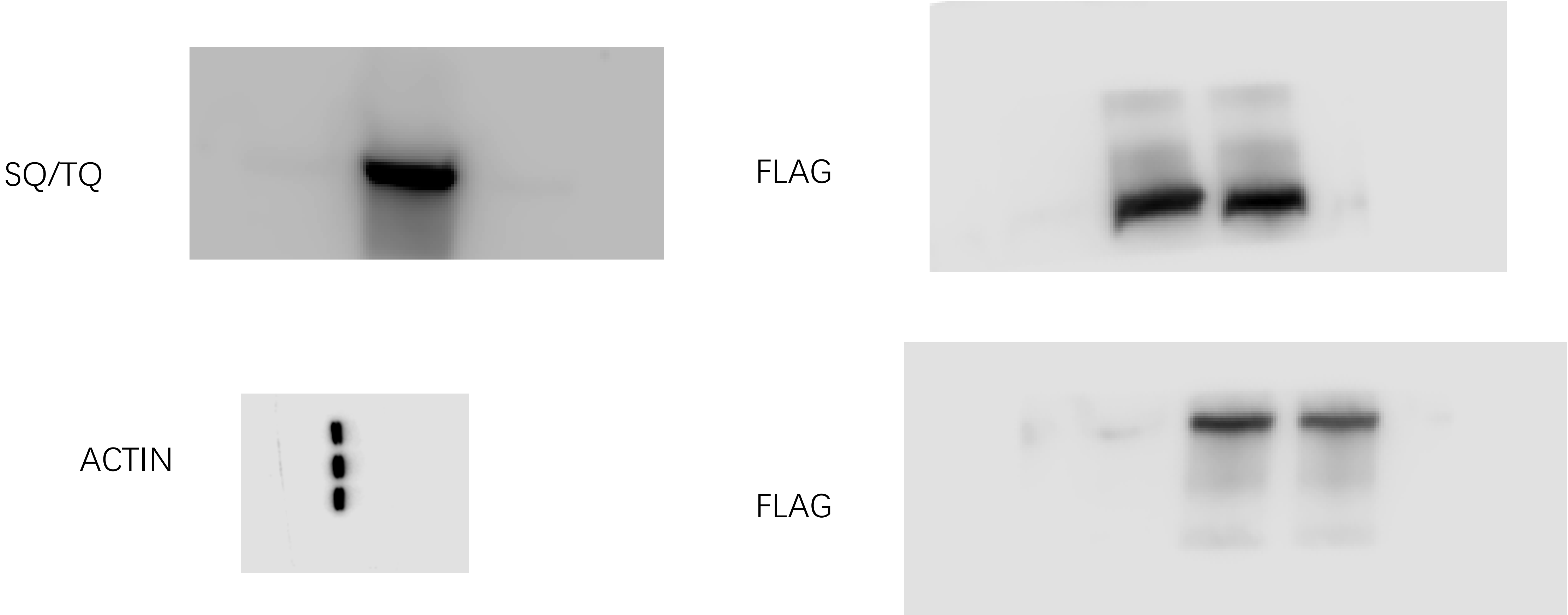

S3E

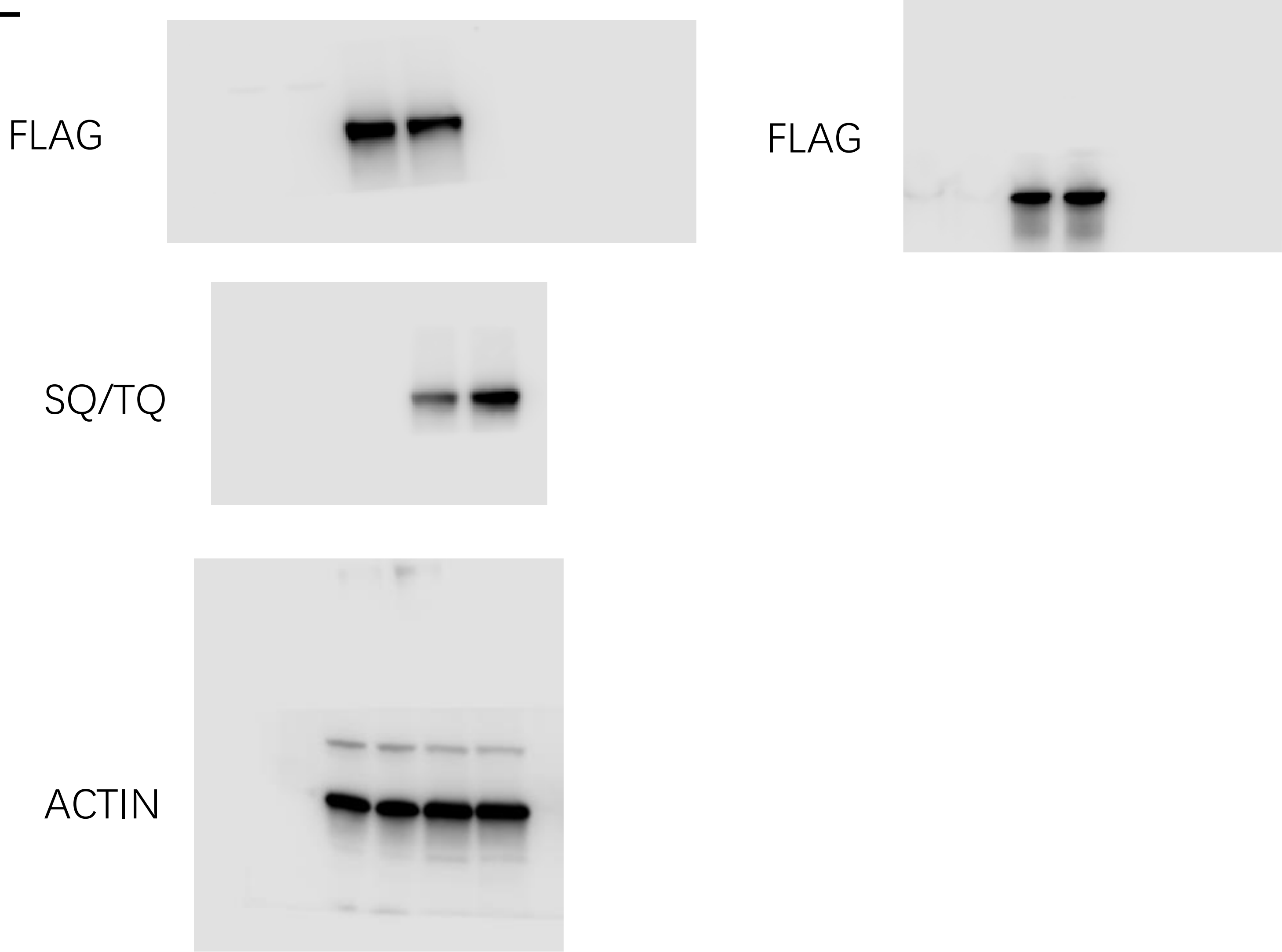

S3G

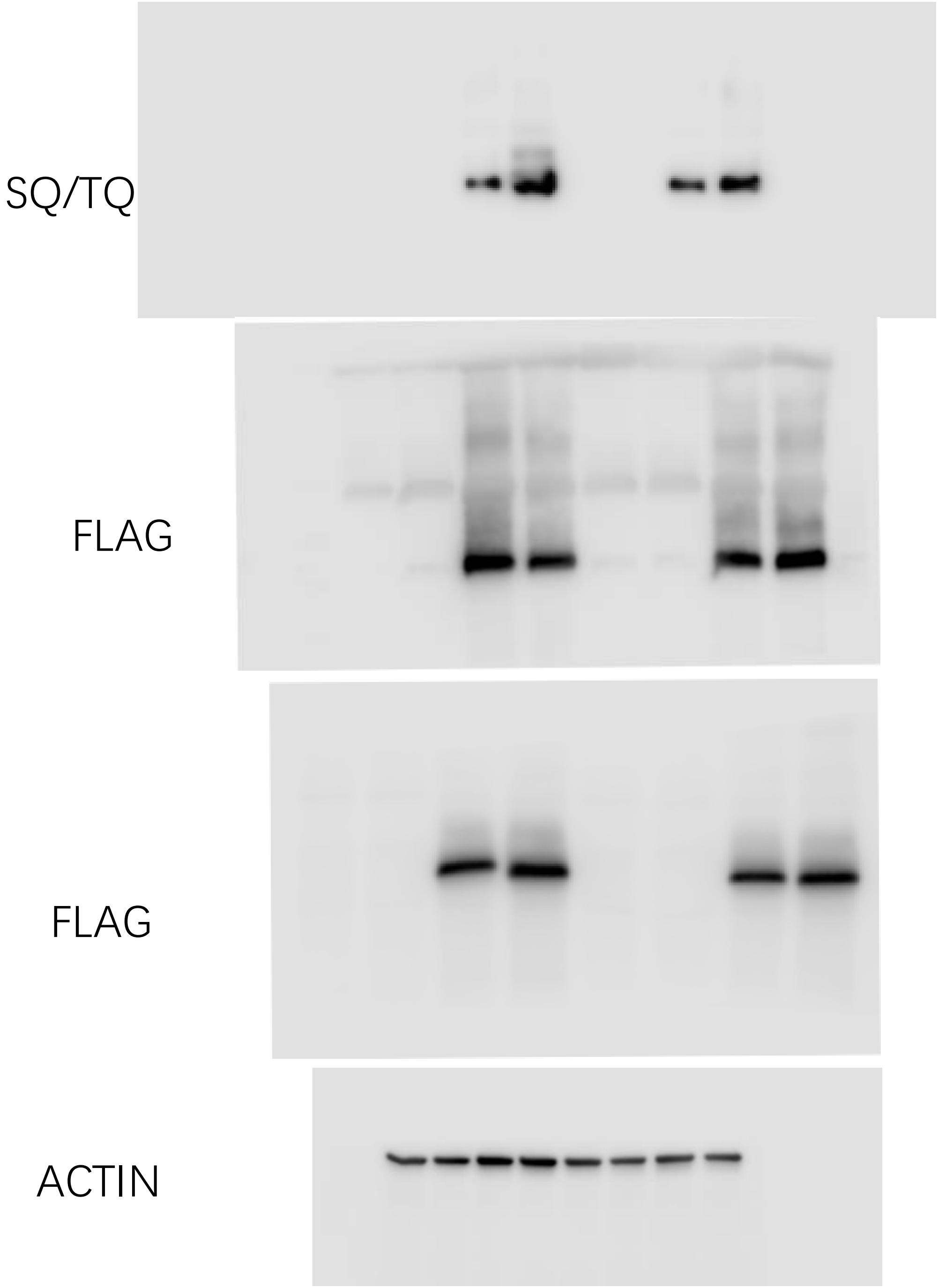

s3i

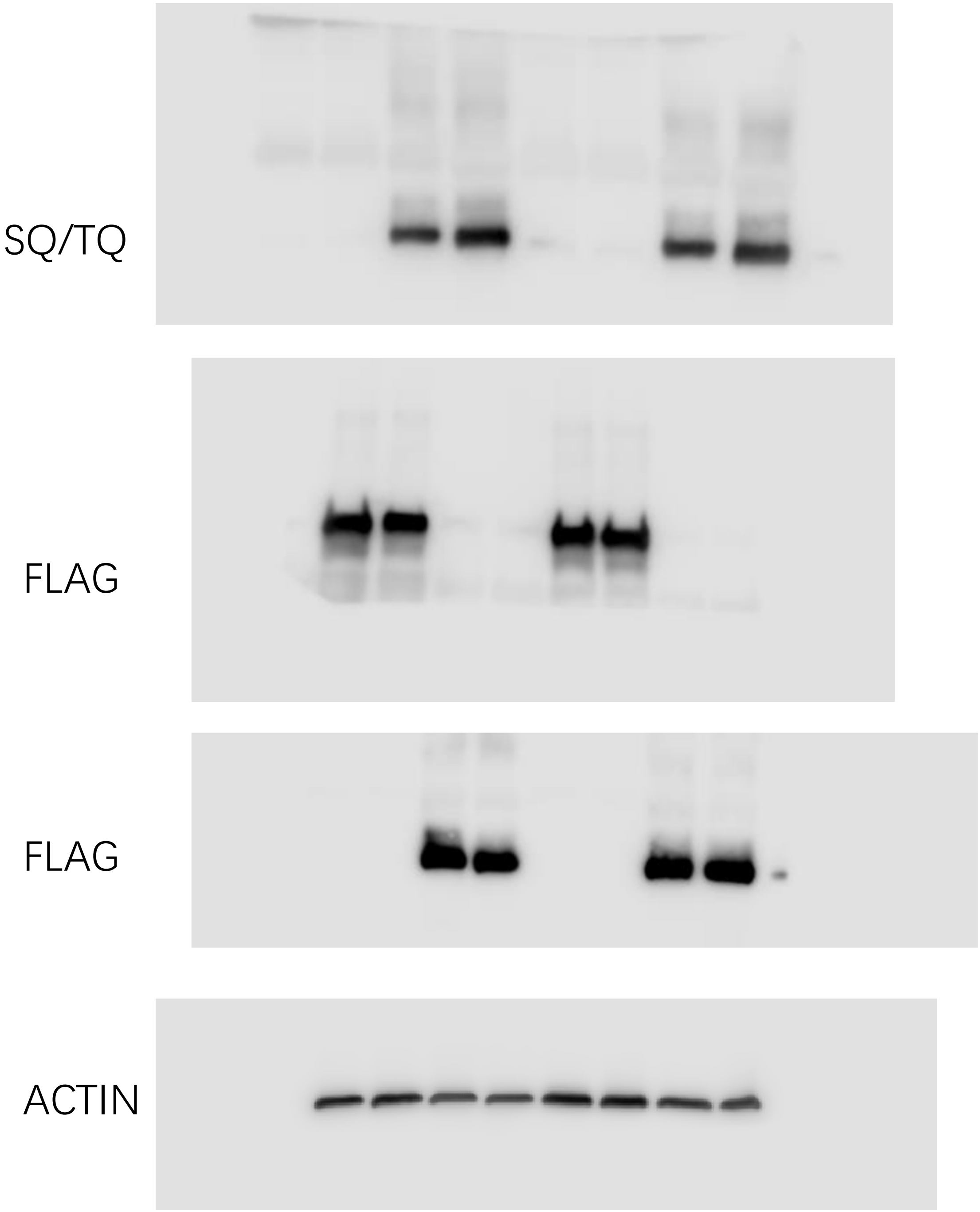

S3K

E-cadherin

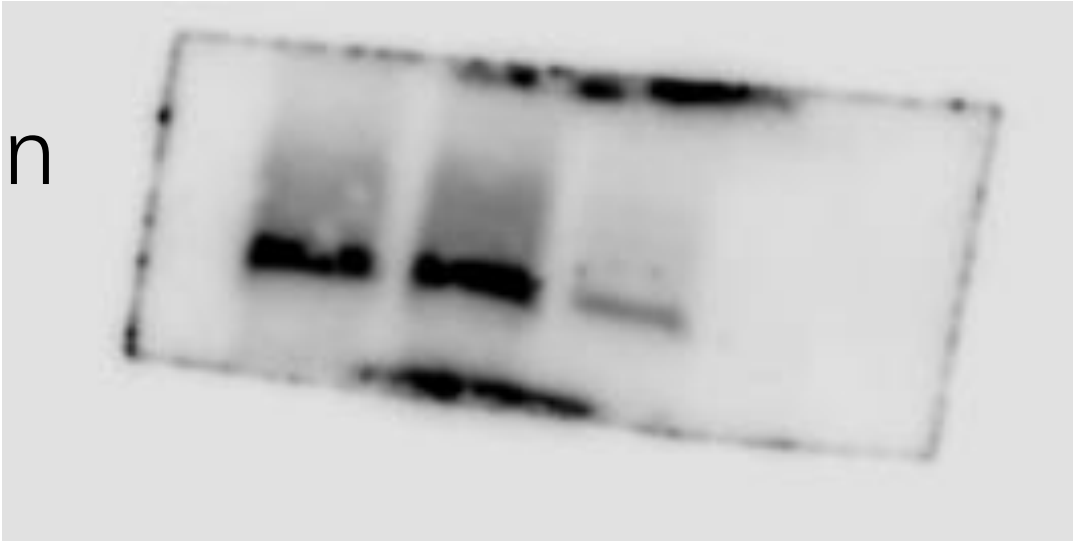

SUN2

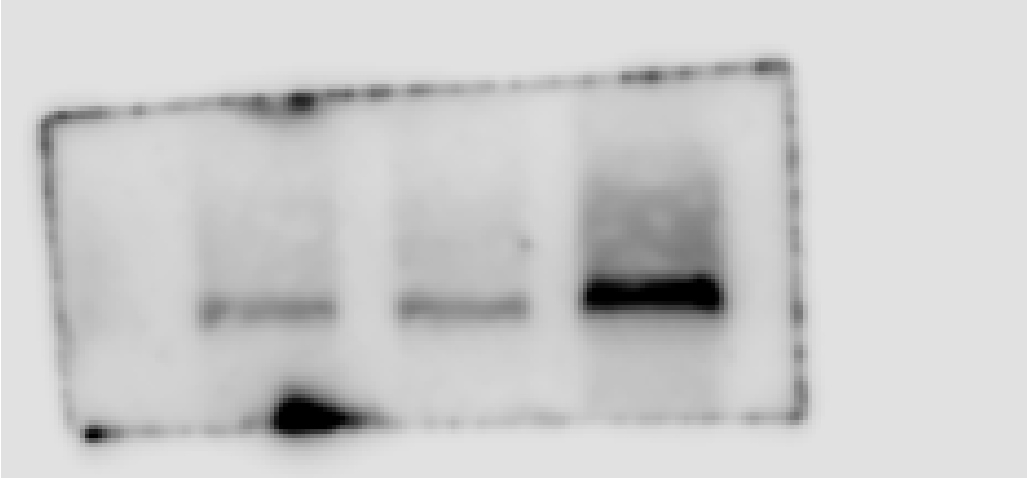

N-cadherin

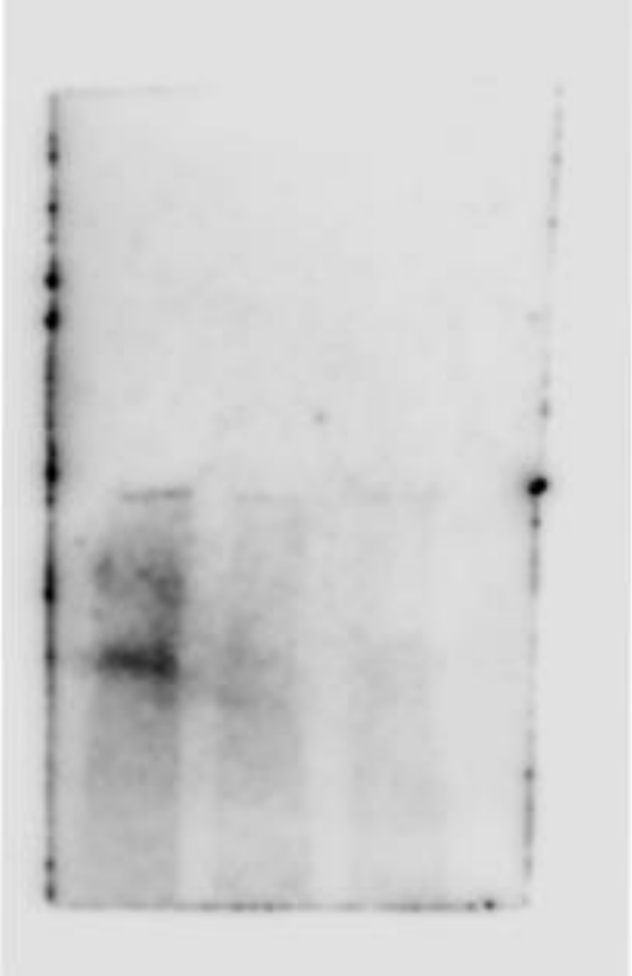

ACTIN

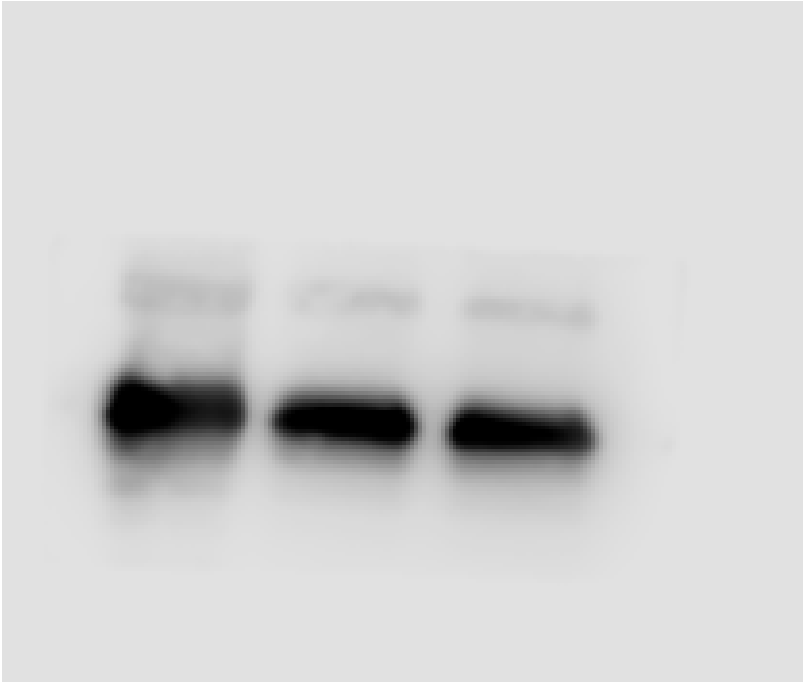

S3M

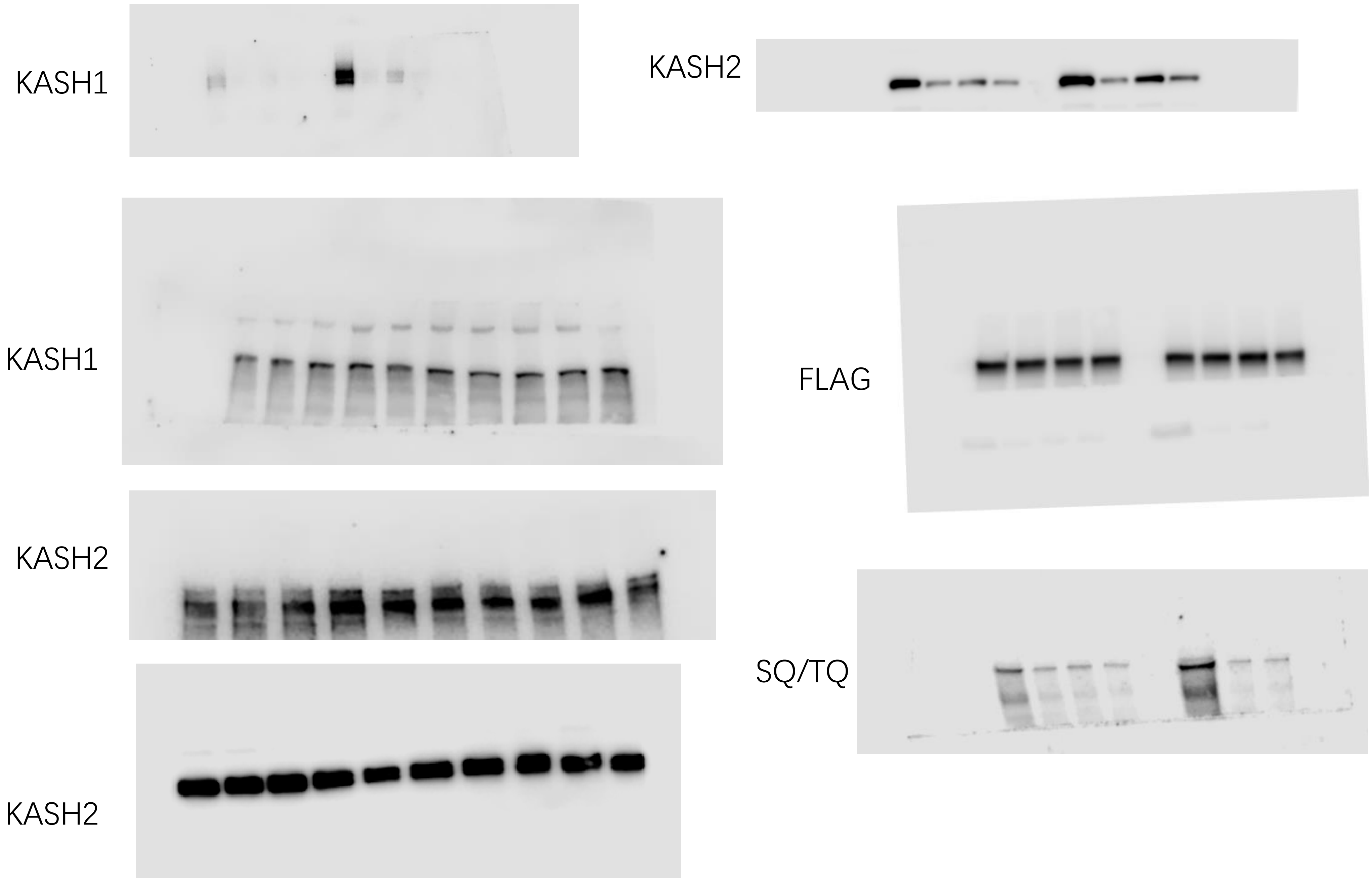

Supplement: Supplementary file 2 [file jci-136-192285-s090.pdf]
